# How Is U.S. Food-Insecurity Related to Dietary Quality? A Scoping Review to Inform Nutrition Security Across the Lifespan

**DOI:** 10.3390/nu18111680

**Published:** 2026-05-24

**Authors:** Analí Morales-Juárez, Jason B. Reed, Olivia Romanovich-Brown, Janet A. Tooze, Heather A. Eicher-Miller

**Affiliations:** 1 Department of Nutrition Science, Purdue University, West Lafayette, IN 47907, USA; moral146@purdue.edu (A.M.-J.); oromanov@purdue.edu (O.R.-B.); 2 Libraries and School of Information Studies, Purdue University, West Lafayette, IN 47907, USA; reed252@purdue.edu; 3 Department of Biostatistics and Data Science, Wake Forest University School of Medicine, Winston-Salem, NC 27101, USA; janet.tooze@wfusm.edu

**Keywords:** food-insecurity, diet quality, nutrient adequacy, nutrition security, life course approach

## Abstract

**Background/Objectives**: This review examined how different levels of U.S. food-security (FS) relate to dietary markers, informing the concept of nutrition security over the lifespan. **Methods**: The authors followed PRISMA-ScR guidelines. PubMed, CINAHL, Scopus, Embase, and CAB Abstracts were searched for eligible U.S.-based, English-language studies examining FS and dietary markers in free-living, disease-free populations, excluding COVID-19-era research. Two reviewers independently screened records in Covidence, with discrepancies resolved by a third reviewer. The percentage of studies evaluating >2 FS levels was determined. Dietary markers were classified into three domains: food and beverage (9 components), nutrient (16 components) and bioactive (2 components) markers. The percentages of studies with significant differences were estimated for each dietary domain. **Results**: Of 1069 records, 78 met full-text eligibility. Among these, 15% evaluated dietary markers across >2 FS levels. Among adults, differences by FS status were observed in 67% of assessed food and beverage components (6 out of 9), 50% of nutrient components (8 out of 16), and all evaluated bioactives (100%; 2 out of 2). Children exhibited differences in all assessed food and beverage components (100%; 9 out of 9) and 29% (2 out of 7) of nutrients by FS level. Adolescents had fewer dietary marker differences than children and adults. Findings among infants, pregnant women and older adults were limited, with no studies for lactating women. **Conclusions**: Low FS level is associated with poorer dietary markers across the lifespan compared with FS. Age-specific differences highlight the need for targeted interventions and nutrition security measures.

## 1. Introduction

Food-security, defined as consistent access to enough food for an active, healthy life, includes not only available resources for food, but also access to food to maintain health [1,2,3]. In 2023, food-insecurity, the situation of limited food access, impacted 13.5% of U.S. households [3] representing a significant public health problem because of the link to poor diet, nutrient intake, and health [4,5]. However, food-security alone may not fully capture the complex relationship between access to food and “an active healthy life”. Nutrition security is an emerging concept that emphasizes the quality of food that is available, encompassing consistent and equitable access to healthy, safe, and affordable foods that are necessary for optimal health and well-being [6,7,8,9,10].

Food quantity and quality are both essential for optimum development and growth of individuals in younger life stages and for maintenance and healthy aging of those in older life stages. Nutrition security, as defined by the U.S. Department of Agriculture (USDA), is focused on the quality of accessible foods, which may be indicated through evaluation of dietary intake and the overall dietary quality [7]. The Healthy Eating Index (HEI) is a measure of dietary quality that determines how closely a group of foods aligns to the recommendations in the Dietary Guidelines for Americans (DGA) [11], which describe evidence-based dietary patterns to promote health and prevent chronic disease [12]. While nutrition security does not directly equate to dietary quality, it similarly emphasizes healthful and nutritious foods to maintain health and prevent chronic disease, including the concepts of variety to promote nutrient adequacy and moderation to limit dietary components that may contribute to disease when consumed in excess [6,7,8,9]. Despite the growing recognition of nutrition security as a critical aspect of food access, the relationship of food-insecurity with dietary intake and quality across the lifespan is poorly understood [6,7,8,9]. Also, there is not a widely accepted and clearly developed measure to quantify nutrition security [6,7,8,9]. Determining the way in which food-insecurity is related to a multitude of dietary outcomes (dietary quality, food group and nutrient intake, bioactive dietary substances, etc.) in various age groups can deepen understanding of the potential ways diets may differ when resources are limited. In addition, nutrition security as a concept at the population level may be informed [13].

The level of food-security is another important aspect that may differentiate the relationship with dietary intake. Classification to 4 levels of food-security can be made on the basis of affirming queries about situations of increasingly severe limited access to food using the U.S. Household Food-Security Survey Module (HFSSM), the official measure used to quantify and monitor U.S. food-security [1]. These levels are food-secure (no issues with food access), marginally food-secure (anxiety about having enough food), low food-secure (reduced quality and variety), and very low food-secure (reduced intake and disrupted eating patterns) [1]. Knowledge of how these food-security levels relate to specific dietary and nutrient shortfalls is also relevant to developing interventions to address these nutrition security gaps and to better understand how nutrition security may be related to food-security.

A challenge to understanding the relationship of food-security with diet and nutrition is the variation in dietary and nutrient requirements for health that exists across the lifespan [14]. Furthermore, the ways that diets and nutrient intakes fall short of goals also vary by age and sex groups. For example, all U.S. girls aged 2–18 years had low intakes of total fruits, total vegetables, seafood & plant proteins according to the DGA [15], and potassium according to adequacy markers. However, food-insecurity in this age group was associated with even lower intakes of whole grains and calcium, choline, magnesium, vitamins A, D, E, and zinc [15]. In contrast, all U.S. men aged 19 years and older had low intakes of vegetables, fruits, and dairy as defined by the DGA [11] and calcium and choline based on adequacy markers [16]. Nevertheless, among those experiencing food-insecurity, intake was increased for added sugars [17] and further reduced for total protein foods, seafood and plant proteins [17], magnesium, potassium, vitamins B6, C, D, and zinc [16]. These diverse results show patterns of poor intake that are different among adolescent girls and men (vitamins A, B6, C, and E) yet some shortfalls are similar (calcium, choline, vitamin D, magnesium, potassium, and zinc). However, the extent of this potential variation or consistency in the relationship of dietary outcomes among the U.S. population and its subgroups is not currently known.

A scoping review compiling and summarizing the evidence of the relationship between food-security with diet is a first step to defining nutrition security with respect to healthful diets, throughout the lifespan. Therefore, the aims of this study were to (1) summarize and evaluate the evidence of the relationship of dietary quality (including a variety of outcomes of adequacy and moderation such as foods and beverages, nutrient, and bioactive compounds) to food-insecurity among the U.S. population and subgroups by age group and life stage (infants, children, adolescents, adults, older adults, pregnancy and lactation) and food-security level. The DGA [11] served as the goal or standard for dietary quality. Another objective was to (2) summarize and evaluate the evidence for the relationship of nutrient adequacy and nutrients recommended for moderate intake with food-insecurity also by age group and food-security level. The Dietary Reference Intake (DRI) [18,19,20,21,22,23] values for the Estimated Average Requirement (EAR) or Adequate Intake (AI) served as the appropriate standards for evaluating the nutrient adequacy of a group. Standardized reference intake values have not been established for most dietary bioactive compounds by the National Academies of Sciences, Engineering, and Medicine [24]. So, studies examining bioactive compounds were evaluated by comparing intakes or reported associates across food-security groups rather than against a defined adequacy threshold.

## 2. Materials and Methods

### 2.1. Protocol and Registration

The review protocol was completed before the review following the Preferred Reporting Items for Systematic reviews and Meta-Analyses extension for Scoping Reviews (PRISMA-ScR) guidelines [25]. The final protocol has been registered in the Open Science Framework and is available at 10.17605/OSF.IO/ZNPCM.

### 2.2. Research Question Framework

The PCC (Population, Concept, Context) framework was used to guide the development of the research question because it aligns with the objective of scoping reviews to map key concepts and address areas of uncertainty. In this review, the “Population” encompasses all U.S. age groups, including infants, children, adolescents, adults, and older adults. The “Concept” focuses on dietary quality and nutrient adequacy, assessed through measures aligned with the DGA and the DRI. The “Context” is defined as food-insecurity in U.S. populations, recognizing that differences in access, availability, and dietary intake may vary across levels of food-security. By organizing the research question using the PCC approach, the review ensures systematic identification and synthesis of evidence relevant to understanding how food-security relates to dietary outcomes across diverse U.S. population subgroups.

### 2.3. Eligibility Criteria

Studies were eligible for inclusion if they reported on U.S. populations of any age group, included participants who were generally healthy or mixed populations (with data extracted only for participants without diagnosed diseases), and incorporated a measure of food-insecurity. Studies conducted outside the U.S. were excluded unless U.S.-specific data could be extracted. For studies including mixed populations, only data pertaining to U.S. populations and relevant subgroups (e.g., food-security vs. food-insecurity) were extracted and included in the analysis. Studies that did not report disaggregated U.S.-specific information were excluded. Studies were required to report dietary outcomes, including any aspect of dietary quality or nutrient adequacy, collected using one or more 24 h dietary recalls or any other dietary assessment tool (e.g., Food Frequency Questionnaire (FFQ)). Eligible studies also employed methods to estimate mean intakes (including simple and adjusted means) or usual intake distributions, such as the National Cancer Institute (NCI) method, Iowa State University Foods Method, Multiple Source Method, or the Statistical Program to Assess Dietary Exposure. Only studies conducted in “free-living” conditions were considered to align with the review objective of examining the relationship between food-security level and habitual dietary intake in real-world settings. Experimental diet studies were excluded because controlled feeding conditions may not reflect typical food access, choice, and constraints associated with food-insecurity, thereby limiting their relevance to the study aims. However, baseline information was considered in randomized controlled trials. Quantitative and mixed-methods studies were included, but only quantitative data were extracted. There were no restrictions on publication date, but studies had to be published in English. Studies conducted during the COVID-19 pandemic period were excluded to minimize the influence of this unique disruption in food access and dietary behavior. This approach was intended to ensure that findings reflect typical conditions by excluding contexts with a similar large-scale food emergency.

### 2.4. Population, Exposure, Comparator, and Context

The review included U.S. populations ranging from infants to older adults. The DGA 2020–2025 defined healthy eating recommendations across the lifespan using specific age ranges to reflect differing nutritional needs. The included studies of this scoping review were categorized according to these life stage definitions, assigning each article to the age group that included the majority of participants (except for pregnancy and lactation), as these groupings provide the framework for life-stage-specific dietary guidance in the DGA. Therefore, infants and toddlers included individuals from birth to 23 months of age, a critical period in which early nutrition supports growth and development [11]. Children and early adolescents are defined as those aged 2 through 13 years, a stage when nutrient-dense foods are essential for continued growth and establishing healthy dietary patterns [11]. Adolescents, aged 14 through 18 years, experience rapid growth and hormonal changes, making adequate intakes of energy and key nutrients particularly important [11]. Adults included individuals aged 19 through 59 years, with dietary guidance focused on maintaining health and preventing chronic disease [11]. Finally, older adults, aged 60 years and more, have specific recommendations that account for age-related changes in metabolism, nutrient requirements, and functional status [11]. Pregnancy and lactation were categorized into separate groups. A few studies and age categorizations combined age groups such as children and adolescents considered ages 1–18 years, adolescents and adults considered ages 12–22 years, and adults and older adults ≥18 years, and multiple age groups <18–>60 years.

The exposure of interest was food-security level, defined as high, marginal, low, and very low food-security, reflecting the range from consistent access to sufficient, safe, and nutritious foods to increasingly constrained access and disrupted eating patterns [1,2,3]. In this review, we summarized food-security across all four levels, when available, to better capture potential gradients in dietary outcomes [1,2,3]. However, studies that operationalized food-security as a dichotomous variable (e.g., food-secure vs. food-insecure) were also included. Thus, high food-security or the combination of high and marginal food-security served as the comparator group.

### 2.5. Dietary Quality and Nutrient Adequacy

Primary outcomes included aspects, measures, or classifications of dietary quality and nutrient adequacy. Dietary quality was assessed using the HEI, including components to get enough of: total and whole fruits, total vegetables, dark green and orange vegetables and legumes, greens and beans, total and whole grains, dairy, milk, total protein foods, seafood and plant proteins, and oils, and components to not get too much of, namely, saturated fats, polyunsaturated and monounsaturated fatty acids, refined grains, sodium, added sugars, and calories from solid fats, alcohol, and added sugars (SoFAAS). Nutrient adequacy was evaluated using the DRIs, including the EAR, AI, Acceptable Macronutrient Distribution Range, and Estimated Energy Requirement. The search included macronutrients (protein, carbohydrates, total fat, polyunsaturated and monounsaturated fatty acids, and saturated fats) and micronutrients such as calcium, vitamins A, C, D, E, K, the B vitamins, copper, iodine, iron, magnesium, manganese, molybdenum, phosphorus, potassium, selenium, sodium, and zinc. These searched measures allowed capture of studies evaluating usual nutrient intake or mean intake adherence to recommended levels specific to age and sex groups and evaluation of dietary adequacy. However, within the articles there were additional dietary components (e.g., minimum dietary diversity, choline, lutein and zeaxanthin, and bioactive dietary substances) that were retained and included in the results.

### 2.6. Information Sources and Search Strategy

Electronic bibliographic databases including PubMed, CINAHL (via EBSCO), Scopus, EMBASE (via Elsevier), and CAB abstracts (via Web of Science) were systematically searched on 20 November 2024. An updated search was conducted on 29 December 2025, prior to final analysis to ensure inclusion of the most recent evidence. The search strategy was initially developed by two researchers with expertise in food-security and nutrient adequacy and subsequently reviewed and refined by a health sciences librarian with review project expertise for comprehensiveness and accuracy. The search string combined keywords and Medical Subject Headings (MeSH) as noted in Supplementary Figure S1. The four searches were combined using AND.

### 2.7. Study Selection and Data Extraction

All identified records were imported into EndNote (The EndNote Team, Philadelphia, PA, USA) and then Covidence (Systematic review software, Veritas Health Innovation, Melbourne, Australia, v2024-2025) for screening. Titles, abstracts and full-text articles were independently screened by two reviewers, with disagreements resolved through discussion and consultation with a third reviewer. Full-text articles were then assessed for eligibility and reasons for exclusion were documented. A standardized data extraction form was used to capture key study characteristics, including author, year, study aims, design, population demographics, data collection methods for both exposure and outcomes, statistical analyses, food-security measurement, dietary outcomes, and major findings. Two reviewers independently extracted data, compared results, and resolved discrepancies through discussion and consultation with a third reviewer. Corresponding authors were contacted for missing or unclear information when necessary.

### 2.8. Data Synthesis

Given the heterogeneity of dietary markers across population subgroups, a narrative synthesis was performed. Data were summarized primarily by age group in the tables and written results (Table S1 and Table 1, Table 2, Table 3 and Table 4). The investigators also identified studies that separated their results by food-security level to examine differences in associations across levels. Table S1 also included participant characteristics, food-security measurement, and dietary outcomes. Differences in dietary quality, nutrient adequacy, and DGA-recommended dietary components (e.g., adequacy and moderation) between food-secure and food-insecure populations were described (Table S1). Various operationalizations of food-security, including categorical, continuous, and individual item-level measures, and by level, related to dietary outcomes were described (Table S1). The synthesis also emphasized subpopulation differences to identify varying patterns in the relationships.

### 2.9. Dietary Harmonization and Percentage Estimation

Given the heterogeneity in how dietary markers were evaluated across studies (e.g., mean intake, usual intake, prevalence estimates, and percentages below the EAR or above the AI), measures were harmonized to enable comparison across studies and life stage groups. For this harmonization, dietary markers from the included studies were categorized into three conceptual domains: food and beverage components (nine components), nutrient components (16 components), and bioactive compounds (two components).

Food and beverage components were classified according to the categories defined in What We Eat in America (WWEIA) [26], the dietary intake component of the National Health and Nutrition Examination Survey (NHANES). The food and beverage components included fruits, vegetables, grains, dairy, protein foods, water/non-sugar beverages, added sugars/sugar-sweetened beverages, solid fats, and energy-dense/discretionary foods. For example, if a study reported dietary outcomes for milk, cheese, and yogurt, they were classified under the dairy category because they all belong to this WWEIA food category. The category “energy-dense/discretionary foods” was created to capture items such as snacks, sweets, and fast food, including those present in mixed dishes, while the remaining categories align with WWEIA definitions. All the food and beverage components identified in the scoping review were mapped into one of these nine WWEIA-aligned categories to ensure comprehensive coverage and conceptual consistency. These components reflected both adequacy measures (e.g., fruits, vegetables, grains, dairy, protein foods, water) and moderation measures (e.g., added sugars, solid fats, and energy-dense/discretionary foods) (Table 2), consistent with the DGA [11].

Nutrient components were classified according to the National Academies of Science, Engineering, and Medicine DRI’s [18,19,20,21,22,23] framework and included energy, macronutrients, and selected micronutrients. Although a broad range of nutrients was reported across studies, only those nutrients identified as being of public health relevance or emphasized in the DGA and that had consistent evaluation across the majority of age groups were retained in the harmonized nutrient domain. These 16 nutrient components included total energy, protein, total fat, saturated fat, carbohydrates, calcium, iron, potassium, sodium, magnesium, zinc, vitamins A, C, D, E, and folate (B9) (Table 3) [11]. Therefore, while all food and beverage components and food groups identified in the review were incorporated into the predefined WWEIA categories, not all reported nutrients were included in the final nutrient component domain percentages that were derived as described below (e.g., cholesterol, copper, docosahexaenoic acid, eicosapentaenoic acid, vitamins B6, B12, thiamin, niacin, riboflavin, K, nutrient-to-energy ratios, and phosphorus).

The bioactive compounds identified in the included studies were classified into two components: carotenoids and alpha-linolenic acid. These were reported as a separate domain due to their exploratory nature and lack of universally established reference intake standards [24] (Table 4).

Within each life stage group, the dietary markers evaluated within the three domains were recorded to determine coverage. For example, a study evaluating yogurt among adults would fulfill coverage of the dairy category so that once satisfied, other studies evaluating yogurt for adults would not add to that denotation. Percentages were also calculated to estimate how many dietary markers were covered in the three domains across age groups. This allowed for clear comparison and prevented counting the same information more than once or artificially increasing the totals. This approach provides a consistent, policy-relevant framework to compare results across studies and age groups. Additionally, for each age group, the number of statistically significant differences was counted within each dietary domain (food and beverage components, nutrients, and bioactive compounds). These counts were then expressed as percentages based on the total number of components measured in each domain. This ensured that only the dietary markers assessed in the studies were included in the calculation.

Table 5 presents the main concepts used in this review to define food-security, food-insecurity, dietary quality, nutrition security, and dietary markers across studies.

## 3. Results

**Figure 1:**
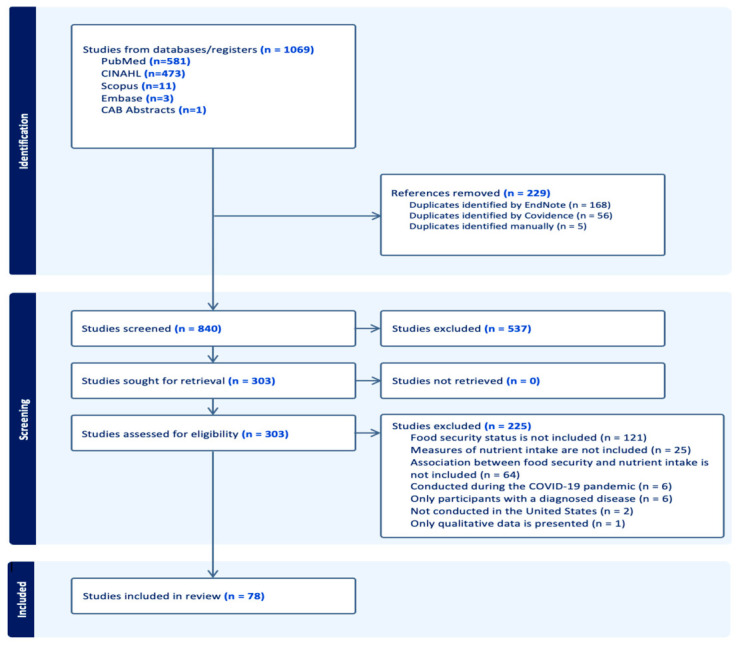
PRISMA-ScR flow diagram for the scoping review on diet quality, nutrients, and dietary components and food-security in the U.S. over the lifespan.

In total, 1069 records were identified through database searches conducted on 20 November 2024 and updated on 29 December 2025. After completing the title and abstract screening, 303 records remained. Subsequent full-text screening resulted in 78 studies included in the overall review. Figure 1 shows the overall study identification and screening process.

### 3.1. General Characteristics of the Studies

Across the 78 studies included in the review, most employed a cross-sectional design, encompassed a wide range of population groups from infants to older adults, and included both males and females. The majority of studies compared diet quality between food-secure and food-insecure groups (e.g., food-secure: high and marginal; food-insecure: low and very low) rather than examining the four levels of food-security status. Dietary intake was most commonly assessed using 24 h dietary recalls and food-security status was predominantly measured using the 18-item U.S. HFSSM and its various forms (e.g., the 10-item U.S. Adult HFSSM) (Table 1).

Dietary markers assessment varied across life stages, with infants, lactating women, pregnant women, and older adults having relatively limited coverage of food and beverage and nutrient components, whereas children, adolescents, and adults were more comprehensively assessed (Table 2, Table 3 and Table 4). Food components and nutrients like cholesterol, copper, docosahexaenoic acid, eicosapentaenoic acid, vitamins B6, B12, thiamin, riboflavin, niacin, and K, nutrient-to-energy ratios, and phosphorus were rarely evaluated, similar to bioactive compounds, particularly among younger and older populations. Comparisons between participants with food-security and food-insecurity revealed that statistically significant differences were more commonly in food and beverage components than in nutrients, and the proportion of significant differences varied by age group (Table 2, Table 3 and Table 4). Particularly, adults demonstrated the most comprehensive coverage of dietary markers, with a greater number of statistically significant differences observed across markers for food and beverage components, nutrients, and bioactive compounds compared with other age groups. Overall, these findings highlight differences and similarities in the number and type of food and beverage, nutrients and bioactive compounds assessed, as well as in the observed associations with food-security status across life stages.

Table S1 provides a summary of the study results for dietary markers that show significant differences between individuals by food-security status across U.S. age groups. Only twelve of the 78 included articles disaggregated dietary markers by the four levels of food-security (e.g., full, marginal, low, and very low). Of these, one focused on children [27], two on children and adolescents [28,29], four on adults [30,31,32,33], and five included adults and older adults [34,35,36,37,38]. The subsequent sections present a detailed narrative of these findings, both by food-security vs. food-insecurity and food-security level, describing the specific food and beverage components, nutrient inadequacies and bioactive compounds that showed statistically significant differences within each age group.

### 3.2. Infants

Only two studies were identified that included infants aged 0–23 months; both reported that food-insecurity was associated with lower diet diversity and poorer diet quality when compared to food-security [39,40] (Table S1). In one of the studies, to assess infant (aged 3–12 months) diet quality, the authors used the World Health Organization’s (WHO) Minimum Dietary Diversity (MDD) score. Using the MDD metric, all solids and liquids consumed in a day were classified into seven food groups: (1) grains, roots, and tubers; (2) legumes and nuts; (3) dairy products (milk, including formula, yogurt, cheese); (4) flesh foods (meat, fish, poultry, liver/organ meats); (5) eggs; (6) vitamin A rich fruits and vegetables; and (7) other fruits and vegetables. MDD was considered to be fulfilled if the infant consumed four or more of the seven food groups on average each day, and unfulfilled if fewer than four groups were consumed. Human milk is not included as a food group in this version of the WHO MDD metric. Regarding MDD, a higher proportion of infants with food-security met the MDD criteria compared with those facing food-insecurity (64.3% vs. 24.3%, *p* < 0.05) (Table S1) [39]. For mean percent daily intake of food groups, infants living with food-insecurity consumed more grains, roots, and tubers (96% vs. 80%, *p* < 0.05), and flesh foods (e.g., meat, fish, poultry, liver/organ meats; 61% vs. 16%) compared with their counterparts living with food-security (16% vs. 61%, *p* < 0.05) (Table S1) [39]. In another study, infants aged 12–23 months affected by food-insecurity had a HEI-Toddlers-2020 score that was 3.78 points lower than their peers with food-security (β = −3.78 ± 1.06; *p* < 0.05) with lower component scores observed for whole fruits (β = −0.56 ± 0.18; *p* < 0.05) and whole grains (β = −0.85 ± 0.35; *p* < 0.05) [40]. Nutrient components and bioactives were not evaluated. Overall, based on two studies, food-insecurity among infants aged 0–23 months was associated with lower diet diversity and poorer diet quality compared with food-security.

### 3.3. Children

Among children aged 2–13 years, 13 studies were identified [27,41,42,43,44,45,46,47,48,49,50,51,52], of which 11 reported that food-insecurity was associated with differences in overall diet quality and food and beverage consumption [27,41,42,43,44,45,47,48,50,51,52] (Table S1). Overall, diet quality, as measured by HEI scores, was modestly but consistently higher among children with food-security compared with their counterparts exposed to food-insecurity. Over a one-year period, girls aged 4–5 years from households that became food-secure had higher total HEI scores compared with girls from persistently food-insecure households (HEI score change = 9.1-points, 95% CI 3.0 to 15, *p* < 0.05) [45]. In another study, children aged 2–8 years with food-security scored higher for adequacy components including total fruits, whole fruits, total vegetables, dark green/orange vegetables and legumes, whole grains, milk, and oils compared with their peers facing food-insecurity [44]. In contrast, the same study showed that children in food-insecure situations had higher scores for adequacy components like total grains, meat and beans, and nutrients recommended for moderate intake like saturated fat, sodium, and SoFAAS components (meaning lower proportional intake) compared with children in food-secure situations (*p* < 0.05) [44]. However, the magnitude of these differences was small; variation across individual HEI components was approximately 1 point for milk and less than 1 point for all other components [44].

Differences were also identified when comparing foods and beverages, for instance, children aged 4–5 years from very low food-secure households consumed higher amounts of foods recommended in moderation like French fries [47], pizza, fried chicken, and hot or ready-made items compared with those from low or food-secure households (*p* < 0.05) [41]. Children aged 1–5 years from food-insecure households also had higher intakes of foods suggested for moderate consumption, including 100% fruit juice, other juices, sweetened beverages, sweet foods (*p* < 0.05) [42], added sugars [48] and soda [52], compared with their peers from food-secure households (*p* < 0.05). In addition, among children aged 9–10 years with food-insecurity, plain water intake was 3.58–4.57 fluid ounces [95% CI: 2.54, 6.63] while estimates for those with food-security were 6.12–7.54 fluid ounces [95% CI: 4.50, 10.48] [43]. Males 8–11 years who experienced food-insecurity were 2.5 times (OR = 2.5, 95% CI =1.1–5.8) more likely to have fewer USDA Food Guide recommended servings of dairy foods compared with peers who experienced food-security (*p* < 0.05) [51].

Regarding nutrient components, protein and energy intake differed by food-security status, for instance children aged 3–5 years facing food-insecurity had lower protein intake (β = −1.3) compared with food-secure peers (*p* < 0.05) [48]. Also, those aged 6–11 years with very low food-security showed greater consumption of calories recommended in moderation, including total energy (β (SE) = 377.1 (169.9)), and percentage of calories from added sugars (β (SE) = 6.8 (2.0)), compared with those with food-security (*p* < 0.05) [27]. Regarding micronutrient intakes, in males aged 8–11 years, the group experiencing food-insecurity had higher odds of consuming calcium below the EAR, with beta coefficients ranging from 1.3 to 4.0 compared with those experiencing food-security [51] (Table S1). Bioactives were not evaluated among children. Overall, these findings suggest that food-insecurity in children aged 2–13 years is associated with small but consistent reductions in diet quality, reflected in lower HEI scores, poorer nutrient adequacy, and a shift toward higher consumption of energy-dense, nutrient-poor foods.

### 3.4. Children and Adolescents

Children and adolescents aged 1–18 years included 11 studies [15,28,29,53,54,55,56,57,58,59,60]. Eight showed associations of food-insecurity with lower diet quality, reduced intake of fruits, vegetables, whole grains, dairy, and protein sources, and higher consumption of foods advised for limited consumption like added sugars and energy-dense foods [15,28,29,53,54,55,56,57] (Table S1). Regarding dietary quality, children and adolescents 1 to 18 years old exposed to food-insecurity had lower dietary quality including total HEI score [55], whole fruits (1.9 ± 0.09 vs. 2.2 ± 0.08), seafood and plant proteins (1.5 ± 0.06 vs. 1.7 ± 0.06) [53] and whole grains (for girls only, 2.5 ± 0.3 vs. 3.1 ± 0.1) compared with peers exposed to food-security [15].

For food and beverage intakes, whole fruit intake ranged from 0.16 to 1.97 servings/day in children and adolescents aged 5–17 years experiencing food-insecurity versus 0.29–2.27 those with food-security [53,56]. In addition, sugar-sweetened beverage and snack consumption, which are foods advised for limited consumption, were consumed in higher amounts in the group with food-insecurity compared with the group with food-security in multiple studies [15,28,56].

Food-insecurity increased the risk of micronutrient inadequacy. Both girls and boys aged 1–18 years with food-insecurity had higher inadequacy for vitamin E, B12, calcium, magnesium, choline, and zinc compared with their peers with food-security [15,57]. Similarly, girls aged 1–18 years experiencing food-insecurity rather than food-security had higher inadequacy of vitamins A and D [15] (Table S1). Bioactives were not evaluated. In summary, among children and adolescents aged 1–18 years, food-insecure conditions compared with food-secure conditions were consistently associated with poorer diet quality, characterized by lower HEI scores, reduced intake of nutrient-dense foods, and higher consumption of foods recommended for limited intake, alongside an increased risk of micronutrient inadequacy.

### 3.5. Adolescents

Only three studies were found among U.S. adolescents aged 14–18 years, and all three consistently reported that food-insecurity was associated with poorer diet quality and reduced nutrient adequacy [61,62,63] (Table S1). In one study, adolescents aged 12–19 years living with food-insecurity compared with food-security, had lower overall diet quality, with adjusted Life’s Essential 8 Diet Score (LE8 Diet score), a score developed by the American Heart Association (AHA), that includes food components for measuring cardiovascular health. Specifically, adolescents aged 15 years with food-insecurity had an LE8 Diet score that was 5.4 points lower [β = −5.4, 95% CI: −8.9, −1.9] (*p* < 0.05) [61] than adolescents with food-security, indicating poorer diet quality. Complementing this finding, another analysis using the HEI-2015 also showed that adolescents aged 12–19 years with food-insecurity scored lower on some dietary components including whole grains (Risk Difference (RD): 0.81; 95% CI: 0.70–0.94) and seafood and plant proteins (RD: 0.82; 95% CI: 0.71–0.95), ultimately resulting in a lower total HEI score (RD: 0.95; 95% CI: 0.93–0.98) than adolescents with food-security [61].

Extending beyond diet quality, differences in specific beverages were also observed. Adolescents aged 14 years experiencing food-insecurity consumed less water and other sugar-free beverages compared with those in food-secure situations (Median (25th, 75th percentiles: 16.0 oz. (8.6, 31.4) and 20 oz. (11.4, 45.7), respectively)) [62], suggesting differences in healthier beverage choices.

Nutrient adequacy scores further reflected disparities. Adolescents 12 to 17 years old with food-insecurity had lower magnesium and potassium intakes, contributing to reduced Total Nutrient Index (TNI) and Food Nutrient Index (FNI) scores compared with adolescents with food-security (*p* < 0.05 for all) [63]. Finally, micronutrient gaps were evident across patterns of egg consumption by food-security status. Adolescents with food-insecurity who were non-egg consumers had lower mean usual intake for lutein+ zeaxanthin, choline, selenium, vitamin D, vitamin B2, docosahexaenoic acid, and protein compared to food-secure adolescents who were egg consumers (*p* < 0.0002), but not compared to food-secure adolescents who were also non-egg consumers [63] (Table S1). In summary, although based on a small number of studies, findings suggest that food-insecurity in adolescents aged 14–18 years is associated with lower diet quality, differences in beverage consumption, and an increased risk of inadequate intake of key nutrients.

### 3.6. Adolescents and Adults

For this age group, only one study was identified, which indicated that adolescents and emerging adults aged 14–22 years living with food-insecurity consumed modestly fewer total fruits and vegetables (1.7 vs. 2.2), whole fruit (0.7 vs. 0.8), vegetables excluding potatoes (1.1 vs. 1.4), dark green vegetables (0.2 vs. 0.3), red/orange vegetables (0.2 vs. 0.3), and whole grains (0.9 vs. 1.1) than those living with food-security [64]. In contrast, intake of foods recommended in limited amounts like sugar-sweetened beverages was slightly higher (0.4 vs. 0.3) for those living in food-insecurity compared with food-security (*p* < 0.05) [61]. Also, food components recommended in moderation like added sugars (33.5 vs. 28.9 g/1000 kcal) and saturated fat (11.1% vs. 10.6% of total energy) were higher for those experiencing food-insecurity compared with food-security (*p* < 0.05) [64] (Table S1).

Nutrient density was also lower among the group experiencing food-insecurity compared with food-security, including potassium (1386 vs. 1505 mg/1000 kcal), vitamin D (94 vs. 104 mg/1000 kcal), calcium (424 vs. 452 mg/1000 kcal), and fiber (9.9 vs. 11.2 g/1000 kcal). Bioactive compounds were not evaluated. The single available study in adolescents and emerging adults aged 14–22 years indicates that food-insecurity is associated with lower dietary adequacy, higher intake of foods recommended in moderation and reduced nutrient adequacy.

### 3.7. Adults

Across U.S. adult populations aged 19–59 years, 19 studies [17,30,31,32,33,52,65,66,67,68,69,70,71,72,73,74,75,76,77] were found and in 17, food-insecurity was associated with poorer overall dietary intake [17,30,31,32,33,52,65,66,67,68,70,71,73,74,75,76,77] (Table S1). Consistent with studies showing that adults exposed to food-insecurity had lower HEI scores compared with their counterparts in food-secure conditions [17,31,33,65,66], one study among young adult college students aged ~19 years reported HEI scores across all four levels of food-security [32]. Participants with high food-security had a mean HEI score of 73.2 ± 20.7, those with marginal food-security scored 79.0 ± 18.6, and those with low food-security scored 71.0 ± 21.3 [32]. HEI scores were lowest among participants with very low food-security, with a mean of 60.1 ± 24.5, indicating poorer overall diet quality in this group [32].

In general, adults aged 19–59 years with food-insecurity had diets characterized by higher consumption of foods recommended in moderation, including ultra-processed foods [30], empty calories [31], added sugars [31,72,73,74], sugar-sweetened beverages [52,73,74], and recommended food components like dairy, compared with food-security [74]. Also, adults living in food-insecurity compared with food-security had lower intakes of total fruits and vegetables [72,77], fruits [73,74,75], vegetables [52,73,74], lean meat [76], fiber [69,74], and whole grains [67]. Differences in nutrient intake were also evident. Adults 25 to 50 years old reporting food-security versus insecurity had a lower proportion of their total calories from carbohydrates [76], while food-secure adults aged 52 years also had 32% lower odds of inadequate carbohydrate intake compared with food-insecure adults [67]. Adults aged 26–49 years with food-insecurity generally consumed fewer long-chain omega-3 fatty acids, including eicosapentaenoic acid, docosahexaenoic acid, and α-linolenic acid and had lower vitamin K, choline, and carotenoid intake, including α-carotene, β-carotene, β-cryptoxanthin, and lutein plus zeaxanthin, compared with their peers with food-security [69]. Adults aged 26–49 years in food-insecure conditions had higher prevalences of inadequacy for multiple vitamins, including A, C, D, E, and several B vitamins such as B6, B12, thiamin, riboflavin, as well as minerals like calcium, iron, magnesium, and zinc when compared to adults in food-secure conditions [69] (Table S1). Overall, among U.S. adults aged 19–59 years, evidence demonstrates a consistent relationship between food-insecurity and poorer diet quality, greater intake of energy-dense nutrient-poor foods, and increased risk of micronutrient inadequacy.

### 3.8. Adults and Older Adults

Studies in adults and older adults aged 19–60+ years numbered 17 [16,34,35,36,37,38,78,79,80,81,82,83,84,85,86,87,88] and 14 of them [16,34,35,36,37,38,78,79,80,82,84,85,86,88] reported that food-insecurity was associated with lower diet quality and poorer nutrient adequacy (Table S1) compared with food-security. Adults and older adults aged 18–75 years with very low food-security consumed a higher proportion of energy from ultra-processed foods, foods recommended in moderation (males: 57% vs. 53%; females: 54% vs. 50%) [37] and fewer fruits and vegetables (3.15 vs. 4.19 servings/day), and less total dietary fiber (12.75 vs. 17.16 g/day) [34,85] than their peers with food-security, respectively. Diet quality indices, including Nutri-Score, HEI, and the AHA score, were consistently lower among adults and older adults aged 19–60+ years experiencing food-insecurity, with reduced component scores for vegetables, fruits, dairy, whole grains, variety, fat, sodium, and hydration compared with their food-secure counterparts [38,78,79,84,88]. Consistent with prior evidence of poorer diet quality among adults and older adults aged 18–80 years experiencing food-insecurity rather than food-security, additional differences were observed across multiple diet quality indices. These indices included the HEI, Alternative Healthy Eating Index (aHEI), Unhealthy Plant-based Diet Index (uPDI), Mediterranean diet (MedDiet), Mediterranean-DASH Intervention for Neurodegenerative Delay (MIND) diet, and Plant-based Diet Index (PDI). Results from such an array of dietary quality metrics showed food-insecurity linked with lower adherence to healthful dietary patterns and lower intake of healthy plant and animal foods among this age group [35,86].

Among adults and older adults, those experiencing food-insecurity versus food-security had lower total HEI-2010 scores (41.3 ± 1.1 vs. 46.1 ± 1.7) [88]. In another study among adults and older adults aged 18–80 years, food-insecurity was highest in the aHEI lowest tertile (39.8%), intermediate in the middle tertile (27.1%), and lowest in the highest tertile (17.7%) compared with food-security, with differences across tertiles (*p* < 0.05) [86]. In another study, adults and older adults aged ≥50 years with low and very low food-security had higher uPDI scores, which included foods recommended in limited amounts like refined grains, sugar-sweetened beverages, and sweets (β = 0.51, 95% CI: −0.22, 1.24; β = 1.46, 95% CI: 0.45, 2.48; *p* < 0.05, respectively) compared with their peers with food-security [34]. In contrast, the MIND diet scores were lower among adults and older adults aged ≥50 years with low and very low food-security (β = −0.15, 95% CI: −0.37, 0.06; β = −0.24, 95% CI: −0.49, *p* < 0.05, respectively) compared with their counterparts with food-security [35]. Also, in the same study, these diet quality indices were assessed in relation to long-term food-insecurity (e.g., long-term food-insecurity was derived by averaging responses to two items from the 18-item HFSSM, assessed biennially between 2002 and 2012). The results showed that very low food-security was linked to lower PDI scores (β = −1.15; 95% CI: −2.37, 0.08), MedDiet (β = −1.33; 95% CI: −2.24, −0.42), and MIND diet scores (β = −0.58; 95% CI: −1.00, −0.17), and higher uPDI scores (β = 1.92; 95% CI: 0.15, 3.69; *p* < 0.05) in contrast with food-security [35]. This longitudinal analysis suggests that persistent food-insecurity has a more pronounced negative link to overall diet quality compared with cross-sectional associations reflective of food-insecurity over the year.

Food-insecurity was also associated with lower nutrient adequacy, with less of the group meeting recommended intakes for protein, carbohydrates, fiber, vitamins A, B6, B12, thiamin, riboflavin, niacin, folate, C, K, iron, magnesium, potassium, phosphorus, selenium, and zinc [16,79,82] (Table S1), yet bioactive compounds were not evaluated. Collectively, these findings indicate that food-insecurity among adults and older adults aged 19–60+ years is associated with higher consumption of ultra-processed foods and moderation components, lower intake of nutrient-dense foods, reduced adherence to dietary recommendations, and diminished nutrient adequacy across multiple dietary assessment measures compared with food-security.

### 3.9. Older Adults

In six out of six studies [89,90,91,92,93,94] among older adults aged ≥60 years, food-insecurity was linked to poorer dietary status (Table S1). Older adults aged ≥60 years with food-insufficiency had lower total HEI scores than older adults with food-sufficiency (β = −1.5 95% CI = −2.84 to −0.12, *p* < 0.05) [89]. A study showed that older adults aged ≥60 years living in limited food-secure environments had higher Nutritional Risk Scores compared with those living in food-secure environments (participants in a food assistance program with food-insecurity: 5.17; participants not in a food assistance program with food-insecurity: 4.15; participants in a food assistance program with food-security: 2.99; participants not in a food assistance program with food-security: 2.90) [90] (Table S1). Another study showed that participants experiencing food-insecurity had lower odds of adherence to the MedDiet (OR = 0.48; 95% CI: 0.31–0.67; *p* < 0.05) and lower HEI-2020 scores (OR = 0.61; 95% CI: 0.37–0.84; *p* < 0.05) compared with participants experiencing food-security [94].

Leung et al. [92] examined different levels of food-security and different diet quality indices. Older adults aged ≥60 years from households facing food-insecure situations had lower HEI-2015 scores (β = −1.90; 95% CI: −3.70, −0.09) when contrasted with a food-secure reference group [92]. For the aHEI, both marginal food-secure (β = −2.42; 95% CI: −3.76, −1.07) and food-insecure situations (β = −1.47; 95% CI: −2.51, −0.44) were associated with lower scores compared with food-secure situations [92]. Similarly, lower MedDiet scores were observed among older adults with marginal food-security (β = −0.62; 95% CI: −1.11, −0.12) and food-insecurity (β = −0.54; 95% CI: −1.06, −0.01) [92] compared with older adults with full food-security.

Regarding nutrients, older adults aged 60–90 years experiencing food-insecurity had lower intakes of calories, protein, carbohydrates, niacin, vitamin B6, vitamin B12, magnesium, iron, zinc, and saturated fat (nutrients recommended for moderation), compared with those experiencing food-security [91]. Yet bioactive compounds were not evaluated in this age group. These findings indicate that food-insecurity among older adults aged ≥60 years is associated with poorer diet quality, higher nutritional risk, and lower adherence to healthy dietary patterns compared with food-security.

### 3.10. Pregnant Women

Across six studies [95,96,97,98,99,100] in pregnant women aged 18–49 years, four [95,96,99,100] reported that food-insecurity was associated with poorer diet quality and less optimal nutrient intake compared with food-security (Table S1). Disparities were reflected in overall diet quality, with lower third-trimester HEI-2015 scores observed among pregnant food-insecure compared with food-secure women aged 18–49 years [99].

Pregnant women aged 24–35 years in food-insecure situations had higher intakes of foods recommended for limited intake including total added sugars, added sugars from sugar-sweetened beverages, and a higher percentage of energy from fat compared with pregnant women in food-secure situations [100]. Pregnant women between 20 and 30+ years old exposed to food-insecurity consumed fewer fruits and vegetables [100] compared with their peers exposed to food-security. Pregnant women aged 22–33 years with very low food-security, contrasted with food-security, reported low vegetable intake, while those with marginal and low food-security consumed more red and processed meat and foods recommended to be limited [96].

Regarding micronutrient intake, results were very limited. Food-insecure pregnant women ≥20 years old had 2.3 times greater odds of having a calcium component score greater than the median intake of calcium scores compared with food-secure pregnant women (Table S1). Of note, iron and folate were also evaluated in one study [95] but no associations with food-insecurity were identified. Bioactives were not evaluated. Collectively, these findings indicate that food-insecurity is negatively related to both overall dietary qualities, while nutrient adequacy in relation to food-security status during pregnancy remains underexplored.

### 3.11. Multiple Age Groups (Household Level)

Findings from one study indicated that food-insecurity in the past month was associated with reduced nutrient intake across multiple age groups and life stages (aged <18 and >60 years), based on household-level dietary marker assessments [101] (Table S1). Specifically, food-insecurity was linked to lower protein intake (β = −0.041 ± 0.015, *p* < 0.05) and a reduced protein-to-energy ratio (β = −0.046 ± 0.015, *p* < 0.05), as well as lower iron intake (β = −0.052 ± 0.022, *p* < 0.05) and a reduced iron-to-energy ratio (β = −0.054 ± 0.022, *p* < 0.05) compared with food-security [101]. Food and beverage and bioactive components were not evaluated.

## 4. Discussion

This review summarized and evaluated evidence on the relationship between food-security, diet quality, and nutrient adequacy across U.S. populations, using a lifespan approach with the DGA and DRI values as standards and with attention to food-security level. The broad age representation of these studies demonstrates growing interest in understanding nutrition security across the life course. By systematically mapping the literature across infancy, childhood, adolescence, adulthood, and older age, this scoping review shows how food-insecurity is linked with deficits in multiple dimensions of dietary markers at different life stages. Notably, substantial gaps for infants, pregnant women, lactating women (no studies), and older age groups limit the ability to build a complete understanding of nutrition-related vulnerabilities across the lifespan. For food and beverage components, participants experiencing food-insecurity consistently exhibited lower intakes of key foods such as fruits, vegetables, grains, dairy, and protein foods in nearly all age groups, including children, adolescents, adults, and older adults, compared to those experiencing food-security. Food-insecure groups also had lower intakes of nutrients including magnesium, zinc, calcium, and vitamin D compared with their counterparts with food-security (only results for calcium applied to pregnant women and results for calcium and vitamin D excluded older adults). Conversely, consumption of added sugars and sugar-sweetened beverages was consistently higher among food-insecure compared with food-secure groups in nearly all age groups (children, adolescents (only when combined with other age groups), adults, older adults, and pregnant women).

While these patterns were broadly consistent across age groups for food and beverage components and select nutrients, more granular age-specific differences were also observed. In children (2–13 y), food-insecurity was associated with lower intake of water/other non-sugar beverages and protein, alongside higher consumption of solid fats and energy-dense foods. Adolescents (14–18 y) with food-insecurity showed lower intake of lutein and zeaxanthin compared with their food-secure peers. Among adults (19–59 y), food-insecurity was linked to lower intake of key micronutrients, including potassium, vitamins A and C, and folate. In adults (19–59 y), food-insecurity was also associated with broader reductions across multiple carotenoids, including α-carotene, β-carotene, β-cryptoxanthin, and lutein and zeaxanthin. In older adults (≥60 y) with food-insecurity, differences encompassed both macro- and micronutrients, with lower intakes of total energy, saturated fat, carbohydrates, protein, and iron compared with those experiencing food-security. Iron and folate intakes were evaluated in only one study among pregnant women, and no associations with food-insecurity were identified.

Across dietary markers, associations of food-insecurity with food and beverage components were reported more consistently than associations with nutrient components. One possible explanation is that several studies (*n* = 24) used NHANES cycles where the samples overlapped and primarily evaluated food group and beverage components, which may have contributed to the replication of similar findings across analyses. Among these 24 studies, only six included analyses of nutrient components [16,51,63,69,82,91], and only two evaluated both food groups and micronutrients [15,95]. Another possible explanation, which should be interpreted cautiously, is that reduced dietary diversity, commonly reported in food-insecure situations [1], may contribute to more consistent detection of disparities at the food group and overall diet quality level than at the individual nutrient level. Limited access to a narrower range of foods may influence overall dietary patterns more uniformly, whereas nutrient-level variation may be partially attenuated by food fortification, mixed food compositions, and compensatory intake across different foods within a day or across dietary assessment periods. Overall, these patterns suggest that the current evidence base, particularly studies using nationally representative U.S. data, may provide insights into overall food and beverage patterns that are less comparable with nutrient-specific disparities associated with food-insecurity.

Specifically, while both children (2–13 y) and adults (19–59 y) had greater coverage of food and beverage components and nutrients compared with other age groups, those experiencing food-insecurity showed distinct patterns by age group. In children, food-insecurity was associated with deficits in diet variety and growth-related nutrients, reflecting disparities in two domains: food and beverage and nutrient components. Bioactive compounds were not evaluated in the available studies for children. In contrast, in adults, food-insecurity was associated with greater reliance on nutrient-poor foods and lower adherence to dietary recommendations. Also, in adults (19–59 y) experiencing food-insecurity, disparities extended across all three domains: food and beverage, nutrients, and bioactive components. Collectively, these findings suggest that food-insecurity is associated with widespread differences in overall diet quality and nutrient intake across the lifespan, rather than being linked to shortfalls in only a few specific foods or nutrients.

A major finding is the noticeable lack of research dedicated to infants, with only two studies examining the dietary relationship to food-insecurity in this population [39,40]. This is a critical oversight, as the first year of life is characterized by rapid physical growth and neurocognitive development, making adequate nutrition essential for long-term health outcomes [102,103]. Optimal nutrition in the first 1000 days can have lifelong effects on the health and well-being of mothers and children [103]. Although the U.S. has made advancements through federal nutrition programs (e.g., the USDA’s Special Supplemental Nutrition Program for Women, Infants, and Children (WIC)) and national initiatives such as the White House Conference on Nutrition, Hunger, and Health [102], research specifically examining infants living in households with food-insecurity remains scarce. WIC participation is associated with reduced household food-insecurity and improved maternal and child health outcomes, including lower rates of preterm birth, reduced infant mortality, and better early childhood diet quality development [104]. However, the program remains underutilized, with only about half of eligible individuals participating and many not fully redeeming benefits [104]. Structural and administrative barriers, including challenges with certification appointments, identifying eligible foods, and stigma, may limit both uptake and benefit redemption development [104]. This gap and the findings of this review highlight the need for greater investment in early-life nutrition research to improve understanding of disparities in infant diet and food access [102]. From a policy perspective, strengthening early-life nutrition protection could include improving WIC accessibility and usability of nutrient-dense complementary foods, and expanding targeted outreach to families with food-insecurity. Also, reducing WIC administrative burdens through strategies such as remote certification, digital tools, and clearer in-store labeling may support program use. Incorporating food-security indicators into early childhood surveillance systems may further support the identification of vulnerable populations and the development of equitable, evidence-driven interventions to promote optimal infant growth and development [104].

This review also highlights important age-specific disparities among youth, where the most meaningful findings contextualized dietary differences against established dietary guidelines and nutrient requirements. In children (2–13 y) and adolescents (14–18 y), food-insecurity was associated with non-adherence to the DGA [11], reflected by lower diet quality scores (e.g., HEI components) and reduced intake of recommended foods, such as whole fruits, total vegetables, whole grains, dairy, and protein sources [11]. Youth living with food-insecurity also showed higher consumption of components that should be limited, including added sugar, saturated fat, and sodium, often reaching levels associated with public health concern [11]. Furthermore, the increased risk of micronutrient inadequacy (e.g., for vitamins A, B12, E, and D, calcium, choline, magnesium, and zinc), for food-insecure youth highlights critical nutrient gaps during growth and development, particularly for nutrients essential to bone and brain health [11]. The dietary and nutrient differences observed by food-security status translate into failure to meet basic nutritional requirements [11], underscoring the severity of the problem.

When examining results exclusively among adolescents, rather than combining adolescents with younger children, fewer dietary markers differed by food-security status. This age group exhibits the poorest overall dietary quality throughout the lifespan, a pervasive finding despite other factors besides food-security such as sex, income level, race, ethnicity, and others [11]. The Scientific Report of the 2025 DGA Advisory Committee identified shortfalls in several nutrients of public health concern among U.S. adolescents, including vitamins A, B6, B12, C, E, folate, phosphorus, choline, and potassium [105], consistent with other reports [15,63]. Adolescents are also less likely to use dietary supplements to help meet nutrient recommendations compared with other life stages [106]. Collectively, these findings suggest that the few dietary differences by food-security status are overshadowed by the very poor dietary and nutrient intake among adolescents that may be related to life stage behaviors or environments.

While the first 1000 days set the foundation for growth and neurodevelopment, the subsequent years of childhood and adolescence represent another period of profound biological, cognitive, and behavioral change, and the establishment of lifelong dietary habits [11,103,107]. Optimal nutrition during this extended developmental window influences immune function, metabolic health, cognitive performance, and the risk of non-communicable chronic diseases (e.g., obesity, diabetes, hypertension, coronary disease, stroke, cancer) that may emerge in advanced ages across the life course [11,103,108,109]. These findings have direct relevance for food assistance programs aimed at youth, suggesting that food-insecurity may limit the effectiveness of dietary guidance when structural barriers are not addressed. The consistent association between food-insecurity and lower diet quality underscores the need to strengthen school meal programs (e.g., National School Breakfast and Lunch Program) by improving nutritional standards and ensuring consistent year-round access, including during weekends and school breaks [109]. In addition, integrating food-security screening into pediatric care and linking families to nutrition assistance programs could help identify at-risk youth earlier and support dietary adequacy during critical developmental stages [110].

Among adults and older adults (19–60+ y), food-insecurity was associated with poorer overall diet quality, as reflected by lower overall HEI scores. It was also linked to higher intakes of food components recommended for consumption in moderation such as saturated fat, sodium, added sugars, and energy-dense foods, which were similarly observed across children and adolescents. Dietary coverage was the most comprehensive among adults (19–59 y) with all food and beverage, nutrient, and bioactive components evaluated. Also, adults (19–59 y) demonstrated a greater number of differences across dietary markers by food-security level compared with infants, children, adolescents, older adults, pregnant and lactating women. Although there were more studies in this age group, this pattern could also possibly reflect true age-related variations in dietary vulnerability as stated in a previous systematic review [5]. Consequently, these findings may indicate either that the adverse dietary effects of food-insecurity are genuinely more pervasive in adults [5], or, alternatively, that nutritional compromise among younger populations is underestimated due to the narrower scope of dietary outcomes assessed in current studies.

Adults (19–59 y) experiencing food-insecurity also showed reduced adequacy of a wide range of nutrients, including fiber, protein, and 13 vitamins and minerals, vitamins A, niacin, riboflavin, thiamin, B6, folate, B12, C, K, phosphorus, magnesium, iron, and zinc compared with their counterparts experiencing food-security. These under-consumed nutrients and the excess consumption of saturated fat, sodium, and added sugars among food-insecure adults have repercussions for metabolic and immune function and heighten the risk of major chronic diseases [107,108,109,110]. In alignment, populations experiencing food-insecurity compared with food-security, had an elevated risk of chronic conditions, including hypertension, coronary heart disease, stroke, diabetes, and cancer, which are leading causes of death in the U.S. [108,111,112,113]. The consistency of these associations underscores the potential role of food-insecurity as a risk factor undermining chronic disease prevention strategies, particularly within nutrition-focused healthcare and community-based interventions [108,111,112,113]. Given that adults experiencing food-insecurity demonstrated reduced adequacy of multiple essential nutrients alongside higher consumption of saturated fat, sodium, and added sugars, policy efforts could focus on improving the nutritional impact of Supplemental Nutrition Assistance Program (SNAP) benefits. This may include incentivizing the purchase of nutrient-dense foods (e.g., fruits, vegetables, and other nutrient-rich options) and extending nutrition education among this life stage group [109]. In addition, strengthening requirements for SNAP-authorized retailers to stock and promote healthier food options, maintaining benefit levels that reflect the cost of a nutritious diet, and sustaining program adaptations such as online purchasing, may further enhance access to healthier foods [109]. Integrating SNAP with nutrition-focused healthcare and community-based interventions could also support chronic disease prevention efforts [109].

Among pregnant women (18–49 y), only six studies were completed [95,96,97,98,99,100] and four of them [95,96,99,100] consistently reported poorer diet quality associated with food-insecurity, including higher consumption of added sugars. Pregnant women exhibited dietary vulnerabilities similar to those observed in adults (19–59 y) when living in food-insecure situations, including lower fruit and vegetable intakes and higher added sugar consumption. There is a near-complete absence of data on lactating women, a life stage characterized by heightened maternal nutrient requirements that are important both for women and developing infants [114]. Such gaps limit the understanding of how food-insecurity may increase nutritional risk during lactation, potentially compromising maternal nutrient status, postpartum recovery, future health, and the quality of breast milk provided to infants [114,115]. Given that nutritional demands increase substantially during pregnancy and lactation, even modest dietary inadequacies in food-insecure contexts may have disproportionate consequences for maternal and fetal or infant health, reinforcing the need for early identification and targeted support during this life stage [114,115]. Among pregnant women (18–49 y), the limited but consistent evidence of poorer diet quality associated with food-insecurity highlights the need to reinforce prenatal nutrition support systems. This includes strengthening WIC’s prenatal component by prioritizing assessment of folate and iron, access to protein-rich foods, expanding nutrition counseling during prenatal care visits [104], and improving coordination between healthcare providers and food assistance programs to address nutritional risk during pregnancy. Despite the absence of data on lactating women, the heightened nutrient demands for both mother and infant [114] and potential risks to development and future health amplify the importance of postpartum WIC support to extend access to nutrient-dense foods that support breast milk quality and maternal recovery [104].

Similarly, despite the elevated nutritional risk in older adults (≥60 y) [116], only six studies [89,90,91,92,93,94] focused exclusively on this population group, with most data derived from mixed adult/older adult populations. The scarcity of age-specific data in older adults (≥60 y) hampers efforts to identify targeted dietary interventions to mitigate risks such as sarcopenia, osteoporosis, and other nutrition-related chronic conditions [116,117]. Policy actions may help address the identified dietary gaps by strengthening programs such as the Meals on Wheels through the Older Americans Act Nutrition by improving access to nutrient-dense foods, particularly protein- and micronutrient-rich options that support muscle maintenance and metabolic health [109]. In addition, integrating food-security screening into routine geriatric and primary care could help identify older adults at risk of sarcopenia, osteoporosis, and other nutrition-related conditions, enabling earlier dietary intervention and support.

This review is strengthened by its comprehensive scope, covering all age groups and a range of dietary intake measures, including nutrient intake and diet quality (diet quality, food group diversity) assessments with attention to the various levels of food-security. Using established standards, such as the DGA and DRI, provides consistent benchmarks for evaluating diet quality and nutrient adequacy. By highlighting these age-specific patterns within a unified framework, this review offers a detailed understanding of how food-insecurity links to diet. The distinct age-specific differences in dietary markers identified in this scoping review suggest that social and behavioral factors may also influence how dietary assistance programs support dietary quality across the lifespan. The Scientific Report of the 2025 DGA Advisory Committee emphasized that nutrition interventions may be more effective when tailored to the cultural, social, and everyday needs of the populations they serve [105]. For example, in this review, adolescents showed fewer dietary differences by food-security status, despite overall poor dietary quality and widespread nutrient inadequacies, suggesting that broader social and behavioral influences may shape dietary intake in this age group regardless of food-security level. Adolescents may positively respond to nutrition programs designed around their unique food environments, peer influences, and increasing independence in food choices. School- or community-based programs that incorporate peer engagement, culturally relevant foods, and practical nutrition skills may better support dietary quality and program participation among adolescents. More broadly, tailoring nutrition assistance strategies to the lived experiences and developmental needs of different population groups may help strengthen the effectiveness of food and nutrition programs across the lifespan [105].

The predominance of cross-sectional designs (approximately 90%) in the reviewed studies limits causal interpretation and conclusions regarding the directional relationship between food-security and dietary quality. Additionally, several findings were derived from cross-sectional studies using overlapping dataset samples, which may have limited the independence of the evidence across studies and relatively few studies evaluated nutrient components using nationally representative survey data. Longitudinal and experimental evidence is needed to clarify how food-security shapes dietary quality and nutrient intake over time [118]. The limited longitudinal data show that persistent food-insecurity is associated with deterioration of diet quality and increased disease risk over time [118]. Even short-term fluctuations in food-insecurity that correspond to short-term dips in diet quality underscore the need for dynamic measures to evaluate these potentially changing relationships [119]. Furthermore, only a small fraction of studies (12 out of 78) examined multiple levels of food-security with only ≤5 studies in an age group. Several studies dichotomized food-security status, often to increase statistical power or due to limited sample sizes within categories. While this approach may enhance analytic feasibility, it limits the ability to draw summaries on dose–response relationships, capture potentially nuanced differences in dietary quality, and inform more targeted interventions across the full spectrum of food-security status and life stages. Without attention to gradients of food-security, policies risk treating food-insecurity as a binary condition, potentially overlooking populations at marginal risk who may already experience meaningful dietary compromise. The exclusion of studies conducted during the COVID-19 pandemic should also be considered when interpreting these findings, as this period potentially entailed specific and unique changes in the food environment and in food access that are not represented here. However, it is important to note that food-insecurity prevalence in the United States decreased in 2020 compared with prior years [3], likely due to expanded federal assistance programs and emergency policy responses.

Another limitation of the literature reviewed is that dietary assessment methods differ in their capacity to capture dietary intake, with recalls providing detailed short-term intake data and FFQs providing participant estimates of usual intake that rely more heavily on participant recall and averaging that introduces error [120]. Applying energy adjustment can improve FFQ-based estimates for certain nutrients but despite this, their estimates include varying degrees of measurement error and bias which affect their accuracy [120]. Self-reported dietary assessment methods are all subject to systematic misreporting bias including the tendency to underreport energy-dense, nutrient-poor foods and overreport healthier foods [120,121,122]. This differential misreporting may lead to measurement error in estimates of dietary intake and attenuate or distort observed associations between food-insecurity and dietary outcomes [120,121,122]. However, estimates derived from repeated administration of the Automated Self-Administered 24 Hour Dietary Recall and 4-day food records have higher capability to estimate intake for select nutrients or dietary components and perform better than FFQs [120]. Applications of statistical approaches such as the NCI method can further reduce within-person variation and improve the estimation of usual intake distributions, thereby strengthening the accuracy and interpretability of dietary intake estimates [121].

Assessment diversity was also present in the food-security assessment methods used across studies, which may have contributed to variability in findings. Although several studies used the USDA 18-item HFSSM with a 12-month reference period, others used abbreviated tools such as the 6-item or single-item screeners, as well as shorter reference periods such as 30 days [1]. Shorter assessment periods may identify fewer experiences of food-insecurity because they capture only more immediate or acute experiences, whereas 12-month measures are more likely to capture intermittent or episodic food-insecurity occurring throughout the year. Differences in instrument sensitivity, classification thresholds, and recall periods may therefore have influenced the detection and magnitude of associations between food-insecurity and dietary outcomes across studies. The potential mismatch between the timeframes used to assess food-insecurity and dietary intake may additionally limit the results included in this review. Food-insecurity is commonly measured over a 12-month period (or sometimes 30 days) [1], while dietary assessment methods are generally designed to capture intake over shorter reference periods depending on the instrument used, as described earlier [120]. This discrepancy may introduce temporal misalignment, limiting the ability to capture concurrent relationships between food-insecurity status and reported dietary intake.

Reliance on published literature may also lead to publication bias, overrepresenting certain populations or outcomes. Another important limitation is that several studies combined multiple age groups, for example, children with adolescents or adults with older adults, rather than analyzing them separately. This aggregation reduces the ability to detect meaningful age-specific differences in dietary intake and food-insecurity, which are central to the goals of this review. Nutritional requirements, eating behaviors, physiological needs, and vulnerability to food-insecurity vary substantially across the life course. Therefore, pooling age groups can mask distinct patterns of nutrient inadequacy and diet quality that are unique to each developmental stage.

## 5. Future Directions

A major need for future research is the coverage of underrepresented groups, including infants, pregnant and lactating women, and older adults. Particular attention to mechanisms such as the relationship or impact of food-insecurity on breast milk quality and nutrient composition, as well as nutrient absorption and utilization in older adults, would also help to fill gaps in the current literature. Also, the stratification of analyses by narrower age categories could better support the development of life-stage-specific measures of nutrition security. A second major need is for investigations to use more than two levels of food-security to make meaningful progress toward understanding dietary disparities, along with their application to developing a nutrition security measure that appropriately reflects these potential variations over the life course. With regard to dietary endpoints, the inconsistent assessment of nutrients including cholesterol, copper, docosahexaenoic acid, eicosapentaenoic acid, vitamins B6, B12, thiamin, riboflavin, niacin, K, nutrient-to-energy ratios, and phosphorus and investigation of bioactives across age groups limits a full characterization of the burden of nutrient inadequacy associated with food-insecurity. This raises the need for more comprehensive nutrient evaluation in future dietary studies.

While the evidence of this scoping review does not, on its own, establish a basis for developing a comprehensive, life-course-sensitive nutrition security metric, it provides essential insights that can guide and strengthen future efforts in that direction [6,7,8,9,10]. Developing such a metric may require rigorous, mixed-methods research capable of capturing the multidimensional nature of nutritional adequacy across diverse population groups [123]. Existing exploratory approaches, often relying on proxy indicators such as inadequate intake of select nutrients or food components, are insufficient to characterize the broader spectrum of dietary quality needed to support healthy growth, development, physiological maintenance, and disease prevention [6,7,8,9,10]. Substantial conceptual refinement and empirical validation are also needed before a robust measurement framework for nutrition security can be adopted for research, surveillance, or policy use [123].

Incorporating comprehensive and valid nutrition security metrics that capture dietary quality, nutrient adequacy, and physiological needs across the life course into existing safety-net programs, public health monitoring, and clinical settings would improve identification of vulnerable populations and support more targeted, evidence-based interventions. Importantly, such assessment is included in a key large-scale national surveillance system, the NHANES, essential for standardized, population-level assessment of diet quality and nutrient adequacy across life stages with the capacity for monitoring nutrition security trends over time.

## 6. Conclusions

In conclusion, this review demonstrates a consistent relationship between food-insecurity and poorer diet quality, reduced nutrient adequacy, and increased consumption of energy-dense, nutrient-poor foods across the U.S. population. Specifically, disparities were observed in intakes of fruits, vegetables, grains, dairy, and protein foods, as well as nutrients such as magnesium, zinc, calcium, and vitamin D (excluding older adults for the last two nutrients) among children, adolescents, adults and older adults experiencing food-insecurity compared with their food-secure counterparts. In contrast, higher intakes of added sugars and sugar-sweetened beverages were also reported among participants facing food-insecurity relative to those with food-security. In addition to the disparities mentioned above, unique differences associated with food-insecurity were also observed for specific age groups, such as lower intake of water/non-sugar beverages and protein intake, and higher intake of solid fats and energy-dense foods in food-insecure compared with secure children (1–13 y). Lower intakes of potassium, vitamins A and C, and folate were found in food-insecure compared with secure adults (19–59 y) and lower intakes of total energy, saturated fat, carbohydrates, protein, and iron in food-insecure compared with secure older adults (≥60 y). Food-insecurity during pregnancy was not broadly assessed in relation to nutrients, including iron and folate intake, limiting the capacity to identify potential disparities. For bioactives, lower intake of lutein and zeaxanthin among food-insecure compared with secure adolescents was noted, while in adults (19–59 y) food-insecurity was associated with lower intake across multiple carotenoids, including α-carotene, β-carotene, β-cryptoxanthin, and lutein and zeaxanthin.

However, the current evidence base remains structurally incomplete with limited scientific evidence for infants, pregnant and lactating women, and older adults, as well as a lack of studies examining multiple levels of food-security. More broadly, nutrient-level outcomes in relation to food-security remain underexplored in studies using nationally representative U.S. data. Although some nutrients have been assessed more frequently than others in city- and state-level studies across age groups, there is overall limited evaluation of a wide range of nutrients across age groups. These include, cholesterol, copper, docosahexaenoic acid, eicosapentaenoic acid, vitamins B6, B12, thiamin, riboflavin, niacin, and K, nutrient-to-energy ratios, and phosphorus across age groups. Addressing these knowledge gaps is critical for informing assistance programs, clinical screening practices, guiding targeted interventions and policies to protect and improve nutritional health across the lifespan. Adults (19–59 y), who had the most comprehensive dietary assessment coverage, exhibited the most consistent disparities between food-secure and food-insecure groups, highlighting a critical need for targeted interventions in this population.

The findings of this review underscore the need for coordinated, life-stage-specific strategies to address food-insecurity and improve dietary quality across the population. Strengthening and better utilizing existing federal nutrition programs, such as WIC, school meal programs, SNAP, and the Meals on Wheels, alongside integrating routine food- and nutrition-security screening into healthcare and community settings, may enhance early identification of at-risk groups and support timely, tailored interventions. Furthermore, advancing the development and integration of comprehensive nutrition security metrics into national surveillance systems is critical to capture dietary adequacy across the life course and guide evidence-based policy. Together, these efforts can support a shift from food-security to nutrition security, ensuring that access to food is accompanied by access to diets that promote optimal health and development.

## Figures and Tables

**Table 1:** General characteristics of studies included in the scoping review on diet quality, nutrients and dietary components and food-security in the U.S. over the lifespan.

Characteristics	Frequency (*n*)	Percentage (%)
Study design ^a^		
Cross-sectional study	71	90
Cohort	2	2
Longitudinal	3	4
Randomized controlled trials	3	4
Sub population group ^b^		
Infants (0–23 months)	2	3
Children (2–13 y)	13	16
Children and adolescents (1–18 y)	11	14
Adolescents (14–18 y)	3	4
Adolescents and adults (14–59 y)	1	1
Adults (19–59 y)	19	24
Adults and older adults (19–60+ y)	17	21
Older adults (≥60 y)	6	8
Pregnant women (18–49 y)	6	8
Multiple age groups (<18–60+ y)	1	1
Sex		
Female	9	12
Female and Male	69	88
Dietary assessment method		
24 h Dietary recall	40	51
ASA24 ^c^	4	5
Food Frequency Questionnaire (FFQ)	15	19
Food Diaries/Food Records	2	3
Other	17	22
Food-security assessment method		
18-item U.S. Household Food-Security Survey Module	37	48
6-item U.S. Household Food-Security Survey Module	15	19
10-item U.S. Adult Household Food-Security Survey Module	2	3
Other	17	22
Food-security levels		
Two levels (e.g., food-security, food-insecurity)	66	85
More than two levels (e.g., high, marginal, low, very low)	12	15

^a^ Although the total number of articles included was 78, the sum of study designs is 79 because one article reported both cross-sectional and longitudinal analyses. ^b^ The sum of study designs is 79 because one article reported separate results for two subpopulation groups including children and adults. ^c^ The Automated Self-Administered 24 h Dietary Recall (ASA24™), an internet-based 24 h dietary recall, with optional staff assistance.

**Table 2:** Harmonized food and beverages identified in studies included in the scoping review on diet quality, nutrients and dietary components and food-security in the U.S. over the lifespan.

Foods and Beverages (9 Constructs)	Infants (0–23 Months)	Children (2–13 y)	Children and Adolescents (1–18 y)	Adolescents (14–18 y)	Adolescents and Adults (14–59 y)	Adults (19–59 y)	Adults and Older Adults (19–60+ y)	Older Adults (≥60 y)	Pregnant Women (18–49 y)	Multiple Age Groups (<18–60+ y)
Adequacy										
Fruits	✓ *	✓ *	✓ *	✓	✓ *	✓ *	✓ *	✓ *	✓ *	
Vegetables	✓	✓ *	✓ *	✓	✓ *	✓ *	✓ *	✓ *	✓ *	
Grains	✓ *	✓ *	✓ *	✓ *	✓ *	✓ *	✓ *	✓ *	✓	
Dairy	✓	✓ *	✓ *	✓	✓	✓ *	✓ *	✓	✓	
Protein foods	✓ *	✓ *	✓ *	✓ *		✓ *	✓	✓ *	✓ *	
Water/non-sugar beverages		✓ *	✓	✓		✓	✓ *		✓	
Moderation										
Added sugars/sugar-sweetened beverages		✓ *	✓ *	✓	✓ *	✓ *		✓ *	✓ *	
Solid fats		✓ *	✓			✓				
Energy dense/discretionary foods		✓ *	✓ *			✓				
Percentage of dietary markers assessed (%) ^a^	56	100	100	78	56	100	67	67	78	0
Among assessed constructs, % with significant differences ^b^	60	100	78	29	80	67	83	83	57	0
Overall diet quality		✓ *	✓ *	✓ *		✓ *	✓ *	✓ *	✓	

✓ Indicates that the dietary marker was assessed. * Indicates a statistically significant difference between groups with food-security and food-insecurity. ^a^ Percentage of food and beverage components assessed (%): calculated as the number of food and beverage components reported in the included studies (indicated by ✓) divided by the total number of constructs (*n* = 9), multiplied by 100 for a given age group. This represents the proportion of food and beverage components that were evaluated in the studies. ^b^ Among assessed constructs, % with significant differences: calculated as the number of food and beverage components that showed statistically significant differences by food-security level (indicated by *), divided by the total number of constructs assessed (indicated by ✓), multiplied by 100. Only constructs measured in the studies were included in the denominator.

**Table 3:** Harmonized nutrient markers identified in studies included in the scoping review on diet quality, nutrients, and dietary components and food-security in the U.S. over the lifespan.

Nutrients (16 Constructs)	Infants (0–23 Months)	Children (2–13 y)	Children and Adolescents (1–18 y)	Adolescents (14–18 y)	Adolescents and Adults (14–59 y)	Adults (19–59 y)	Adults and Older Adults (19–60+ y)	Older Adults (≥60 y)	Pregnant Women (18–49 y)	Multiple Age Groups (<18–60+ y)
Energy										
Total energy			✓			✓	✓ *	✓ *		
Macronutrients										
Total fat		✓	✓			✓	✓	✓		
Saturated fat		✓	✓	✓	✓ *	✓	✓	✓ *		
Carbohydrate			✓			✓	✓ *	✓ *		
Protein		✓ *	✓	✓		✓	✓ *	✓ *		✓ *
Minerals										
Calcium		✓ *	✓ *	✓	✓ *	✓ *	✓	✓	✓ *	✓
Iron			✓	✓	✓	✓	✓ *	✓ *	✓	✓ *
Potassium		✓	✓	✓	✓ *	✓ *	✓ *			✓
Sodium			✓	✓	✓	✓	✓ *	✓		
Magnesium			✓ *	✓		✓ *	✓ *	✓ *		
Zinc			✓ *			✓ *	✓ *	✓ *		
Vitamins										
Vitamin A			✓ *	✓		✓ *	✓ *	✓		
Vitamin C		✓	✓	✓		✓ *	✓ *	✓		
Vitamin D		✓	✓ *	✓ *	✓ *	✓ *	✓ *			
Vitamin E			✓ *	✓		✓	✓	✓		✓
Folate (Vitamin B9)			✓			✓ *	✓	✓	✓	
Percentage of nutrient markers assessed (%) ^a^	0	44	100	69	38	100	100	88	19	31
Among assessed constructs, % with significant differences ^b^	0	29	38	9	67	50	69	50	67	40

✓ Indicates that the nutrient marker was assessed. * Indicates a statistically significant difference between groups with food-security and food-insecurity. ^a^ Percentage of nutrient markers assessed (%): calculated as the number of nutrient markers reported in the included studies (indicated by ✓) divided by the total number of constructs (*n* = 16), multiplied by 100 for a given age group. This represents the proportion of nutrient markers that were evaluated in the studies. ^b^ Among assessed constructs, % with significant differences: calculated as the number of nutrient markers that showed statistically significant differences by food-security level (indicated by *), divided by the total number of constructs assessed (indicated by ✓), multiplied by 100. Only constructs measured in the studies were included in the denominator.

**Table 4:** Harmonized bioactive markers identified in studies included in the scoping review on diet quality, nutrients, and dietary components and food-security in the U.S. over the lifespan.

Bioactive (2 Constructs)	Infants (0–23 Months)	Children (2–13 y)	Children and Adolescents (1–18 y)	Adolescents (14–18 y)	Adolescents and Adults (14–59 y)	Adults (19–59 y)	Adults and Older Adults (19–60+ y)	Older Adults (≥60 y)	Pregnant Women (18–49 y)	Multiple Age Groups (<18–60+ y)
Carotenoids										
α-carotene						✓ *				
β-carotene						✓ *				
β-cryptoxanthin						✓ *				
lutein and zeaxanthin				✓ *		✓ *				
α-linolenic acid						✓ *				
Percentage of bioactive markers (%) ^a^	0	0	0	50	0	100	0	0	0	0
Among assessed constructs, % with significant differences ^b^	0	0	0	100	0	100	0	0	0	0

✓ Indicates that the bioactive marker was assessed. * Indicates a statistically significant difference between groups with food-security and food-insecurity. ^a^ Percentage of bioactive markers assessed (%): calculated as the number of bioactive markers reported in the included studies (indicated by ✓) divided by the total number of constructs (*n* = 2), multiplied by 100 for a given age group. This represents the proportion of bioactive markers that were evaluated in the studies. ^b^ Among assessed constructs, % with significant differences: calculated as the number of bioactive markers that showed statistically significant differences by food-security level (indicated by *), divided by the total number of constructs assessed (indicated by ✓), multiplied by 100. Only constructs measured in the studies were included in the denominator.

**Table 5:** Main concepts in the scoping review on diet quality, nutrients and dietary components and food-security in the U.S. over the lifespan.

Concept	Definition
Food-security	Consistent access to enough food for an active, healthy life
Food-insecurity	A situation of limited food access, linked to poor diet, nutrient intake, and health
Dietary quality	Refers to how well dietary intake aligns with established dietary recommendations, reflecting the overall adequacy, balance, and healthfulness of the diet. It is commonly assessed using the Healthy Eating Index (HEI), which evaluates the extent to which dietary patterns conform to the Dietary Guidelines for Americans (DGA) aimed at promoting health and preventing chronic disease
Nutrition security	Consistent and equitable access to healthy, safe, and affordable foods that are necessary for optimal health and well-being, emphasizing the quality of food that is available,
Dietary markers	Specific indicators used to characterize specific aspects of the diet, grouped into three conceptual domains to facilitate analysis and comparison: (1) food and beverage components, (2) nutrient components, and (3) bioactive compounds
Food and beverage components	Groups of foods and drinks used to describe dietary intake. Items were grouped into 9 components using the What We Eat in America (WWEIA) system from the National Health and Nutrition Examination Survey (NHANES), including (1) fruits, (2) vegetables, (3) grains, (4) dairy, (5) protein foods, (6) water and non-sugary drinks, (7) sugary drinks and added sugars, (8) solid fats, and (9) energy-dense or discretionary foods
Nutrient components	Nutrients in foods that the body needs for energy, growth, and health classified using the Dietary Reference Intakes (DRIs). Only 16 nutrients were prioritized for public health and consistently reported in the studies across age groups: (1) total energy, (2) protein, (3) total fat, (4) saturated fat, (5) carbohydrates, (6) calcium, (7) iron, (8) potassium, (9) sodium, (10) magnesium, (11) zinc, and vitamins (12) A, (13) C, (14) D, (15) E, and (16) folate.
Bioactive components	Substances in foods that can affect health, but are not considered nutrients like vitamins or minerals, including two components: (1) carotenoids and (2) alpha-linolenic acid. These components were treated as a separate group because they are still being studied and do not have widely established intake recommendations

## Data Availability

The original contributions presented in this study are included in the article/Supplementary Material. Further inquiries can be directed to the corresponding author.

## Supplementary Materials

The following supporting information can be downloaded at: https://www.mdpi.com/article/10.3390/nu18111680/s1, Supplementary Figure S1: Full search strategy for the scoping review on dietary quality, nutrients, and dietary components and food-security in the U.S. over the lifespan, Table S1: Summary of findings from included studies in the scoping review evaluating diet quality, nutrients and dietary components and food-security in the U.S. over the lifespan.

## Abbreviations

The following abbreviations are used in this manuscript:

USDA	U.S. Department of Agriculture
HEI	Healthy Eating Index
DGA	Dietary Guidelines for Americans
HFSSM	U.S. Household Food-security Survey Module
DRI	Dietary Reference Intake
EAR	Estimated Average Requirement
AI	Adequate Intake
PRISMA-ScR	Preferred Reporting Items for Systematic reviews and Meta-Analyses extension for Scoping Reviews
PCC	Population, Concept, Context
FFQ	Food Frequency Questionnaire
NCI	National Cancer Institute
SoFAAS	Solid fats, alcohol, and added sugars
MeSH	Medical Subject Headings
WWEIA	What We Eat in America
NHANES	National Health and Nutrition Examination Survey
WHO	World Health Organization’s
MDD	Minimum Dietary Diversity
LE8	Life’s Essential 8 Diet Score
AHA	American Heart Association
TNI	Total Nutrient Index
FNI	Food Nutrient Index
aHEI	Alternative Healthy Eating Index
uPDI	Unhealthy plant-based diet index
MedDiet	Mediterranean diet
MIND	Mediterranean-DASH Intervention for Neurodegenerative Delay
PDI	Plant-based diet index
WIC	USDA’s Special Supplemental Nutrition Program for Women, Infants, and Children
SNAP	Supplemental Nutrition Assistance Program
