# Supplementary material for: How Is U.S. Food-Insecurity Related to Dietary Quality? A Scoping Review to Inform Nutrition Security Across the Lifespan
Source: Nutrients. 2026 May 24;18(11):1680. doi: 10.3390/nu18111680 (PMC13258701; doi:10.3390/nu18111680)
Supplement: Supplementary file 1 [file nutrients-18-01680-s001.zip › Supplementary Figure S1.pdf]

Population	#1	infant OR children OR adolescent OR adult OR elderly OR aged
	#2	"United States"
Concept	#3	"diet quality" OR "nutrient adequacy" OR "dietary intake" OR "dietary behavior" OR "dietary selection" OR "Healthy Eating Index" OR "total usual nutrient intake" OR "inadequate intake" OR "adequate intake" OR "total intake" OR "food consumption patterns" OR "usual nutrient intake" OR "dietary measures" OR "Estimated Average Requirement" OR "diet adequacy" OR "dietary reference intakes" OR "Dietary Guidelines for Americans" OR "nutrient intake" OR "food group intake" OR "underconsumed nutrients" OR "dietary quality" OR "micronutrient intake" OR "recommended dietary allowances"[MeSH Terms]
Context	#4	"food insecure" OR "food secure" OR "food sufficiency" OR "low-income population" OR "food purchasing" OR "SNAP participants" OR "Supplemental Nutrition Assistance Program" OR "nutrition assistance program" OR "emergency food assistance" OR "food pantry" OR "food stamps" OR "food insecurity"[MeSH Terms] OR "food security"[MeSH Terms] OR "food deserts"[MeSH Terms] OR "social determinants of health"[MeSH Terms] OR "poverty ratio" OR "income level" OR "Child and Adult Care Food Program" OR "The Food and Nutrition Service Office of Food Safety" OR "National School Lunch Program" OR "School Breakfast Program" OR "Special Milk Program" OR "Team Nutrition" OR "Commodity Supplemental Food Program" OR "Food Distribution Program on Indian Reservations" OR "The Emergency Food Assistance Program" OR "Senior Farmers' Market Nutrition Program" OR "Older Americans Act Nutrition Programs" OR "Special Supplemental Nutrition Program for Women, Infants, and Children" OR "Farmers' Market Nutrition Program" OR "USDA's Food and Nutrition Service"

Figure S1. Full search strategy for the scoping review on dietary quality, nutrients, and dietary components and food-security in the U.S. over the lifespan.
