# Supplementary material for: How Is U.S. Food-Insecurity Related to Dietary Quality? A Scoping Review to Inform Nutrition Security Across the Lifespan
Source: Nutrients. 2026 May 24;18(11):1680. doi: 10.3390/nu18111680 (PMC13258701; doi:10.3390/nu18111680)
Supplement: Supplementary file 1 [file nutrients-18-01680-s001.zip › Supplementary Table S1.pdf]

Supplementary Material

Table S1. Summary of findings from included studies in the scoping review evaluating diet quality, nutrients and dietary components and food-security in the U.S. over the lifespan

Author, year, location (state)	Study design, participant characteristics	Food-security assessment method	Dietary assessment method	Dietary marker measured	Significant main findings: Is food-security associated to dietary intake?	Definition/Description
			**Infants**			
Campbell, 2020, O'ahu (Hawai'i)	Cross-sectional study, n = 70, Female, Male, 3-6, 6-12 months	The infant food-security index, a multidimensional Index developed from cohort data in New Zealand, was adapted to estimate the degree of infant food-security [a]	The global metric MDD [b] score from the World Health Organization's. The application Mobile food record mFR was used to collect the dietary intake. Caregivers were instructed to take before and after images of all foods and beverages the participant consumed over a 4-day collection period (Thursday–Sunday)	<u>Estimated mean percentage of MDD [b]</u> Using the MDD [b] metric, solids and liquids consumed in a day were categorized into seven food groups: 1) grains, roots, and tubers; 2) legumes and nuts; 3) dairy products (milk, including formula, yogurt, cheese); 4) flesh foods (meat, fish, poultry, liver/organ meats); 5) eggs; 6) vitamin A-rich fruits and vegetables; and 7) other fruits and vegetables	Yes <u>Estimated mean percentage of MDD [b], $P< 0.05$</u> <u>Met MDD [b], n (%)</u> FI [c]: 9 (24.3), FS [d]: 9 (64.3) <u>Did not meet MDD [b] n (%)</u> FI [c]: 28 (75.7), FS [d]: 5 (35.7) <u>Food components of the MDD</u> Grains, roots, and tubers: FI [c]: 96%, FS [d]: 80%, flesh foods: FI [c]: 61%, FS [d]: 16%	[a] The adapted infant food-security index is a weighted score based on two U.S. Household Food-security Survey Module (HFSSM) items: use of coping strategies for food-security and infant breastfeeding status at 3 months. Points were awarded for breastfeeding to 3 months and for never running out of money for food or utilities. The scale ranged 0–18, with higher values indicating greater food-security; [b] Minimum Dietary Diversity (MDD); [c] Food insecurity (FI); [d] Food security (FS); [e] National Health and Nutrition Examination Survey (NHANES) is a continuous, cross-sectional series of surveys of nationally representative samples of the resident, civilian, non-institutionalized US population; [f] Healthy Eating Index (HEI) measures adherence to the Dietary Guidelines for Americans based on adequacy and moderation components, with higher scores indicating better diet quality; [g] Adjusted for sociodemographic, food secure as the reference category; [h] Standard error (SE)
Sanjeevi, 2025, NHANES [e] from the United States	Cross sectional study, n = 708, Female, Male, 12-23 months	18-item U.S. Household Food-security Survey Module (HFSSM)	At least one 24-hour dietary recall with a trained interviewer and a second recall conducted for most participants by telephone 3-10 days later	<u>Estimated mean HEI [f]–Toddlers–2020</u> Total fruits, whole fruits, total vegetables, greens and beans, whole grains, dairy, total protein foods, seafood and plant proteins, fatty acids, refined grains, sodium, added sugars, and saturated fats, total score	Yes <u>Multivariate [g] analysis of the association between FS [d] and the HEI [f] total score and food components, $\beta \pm SE$ [h], $P<0.05$</u> FI [c]: HEI [f]-Toddlers-2020: -3.78 ±1.06, whole fruits: -0.56±0.18, whole grains: -0.85 ±0.35	

Author, year, location (state)	Study design, participant characteristics	Food-security assessment method	Dietary assessment method	Dietary marker measured	Significant main findings: Is food-security associated to dietary intake?	Definition/Description
			**Children**			
Au, 2019, 130 Diverse United States Communities	Cross sectional study, n = 5138, Female, Male, 9 y	A validated 2-item screener derived from the 18-item U.S. Household Food-security Survey Module (HFSSM)	DSQ [a] a FFQ screener that assesses intake over the past 30 days, developed by the NCI [b]	Estimated mean intake of dietary markers<br>Fruit/vegetable/legume no fried potatoes, whole grains, fiber, dairy, calcium, total added sugar, sugar for sugar-sweetened beverages, energy-dense foods of minimal nutritional value	Yes<br>Estimated mean intake of dietary markers ± SD [c]<br>Fruit/vegetable/legume: FS [d]: 2.5 ± 0.9 FI [e]: 2.4 ± 0.9, fiber, FS [d]: 15.6 ± 3.8 FI [e]: 15.4 ± 4.1, total added sugar, FS [d]: 72.7 ± 27.7 FI [e]: 80.4 ± 35.2, sugar from sugar-sweetened beverages , FS [d]: 25.6 ± 16.5 FI [e]: 30.9 ± 21.6, energy-dense foods of minimal nutritional value, FS [d]: 1.9 ± 1.7 FI [f]: 2.0 ± 2.0<br><br>Multivariate [f] analysis of the association between FS [d], and dietary markers, β (95% CI), P<0.05<br>FI [e]: sugar from sugar-sweetened beverages, β=1.44 (0.35, 2.54)	[a] The Dietary Screener Questionnaire (DSQ), a 26-item food frequency questionnaire developed by the [b] National Cancer Institute (NCI) was administered. The DSQ assesses intake on selected foods consumed over the past 30 d in number of times per day, week, or month. NCI-generated scoring algorithms were used to convert reported frequencies to estimated quantities of select food groups and nutrients, based on age- and sex-specific 24-h dietary recall portion size data from National Health and Nutrition Examination Survey (NHANES); [c] Standard Deviation (SD); [d] Food security (FS); [e] Food insecurity (FI); [f] Adjusted for sociodemographic, food secure as the reference category<br>.
Bauer, 2012, On or near the Pine Ridge Reservation (South Dakota)	Cross sectional study, child-parent, dyad n = 432, Female, Male, 4-6 y	6-item Short Form U.S. Household Food-security Survey Module (HFSSM)	Food Frequency Questionnaire (FFQ), parents reported the frequency of their child's consumption of specific foods and beverages over the past month	Estimated mean intake of food groups<br>Fruit, vegetables, french fries or fried potatoes, pizza, fried chicken, sugar-sweetened beverages, sweet and salty snacks, skim or 1% milk, whole or 2% milk, chocolate milk, fast food, hot or ready-made foods from a convenience store or gas station	Yes<br>Estimated mean intake of food groups, P<0.05<br>Pizza, times/week: very low FS [d]:1.68, low FS [d]:1.32, FS [d]: 1.12, fried chicken, times/week: very low FS [d]:1.16, low FS [d]:1.03, FS [d]: 0.78, hot or ready-made foods, times/week: very low FS [d]:1.30, low FS: 0.96, FS [d]: 0.64	

Author, year, location (state)	Study design, participant characteristics	Food-security assessment method	Dietary assessment method	Dietary marker measured	Significant main findings: Is food-security associated to dietary intake?	Definition/Description
			Children			
Chaparro, 2022, Los Angeles County (California)	Cross sectional study, n = 9929, Female, Male, 2.5 y	6-item Short Form U.S. Household Food-security Survey Module (HFSSM)	Caregivers were asked questions about their children's consumption of various food groups [a]	Estimated mean intake of food groups<br>Fruit, 100% fruit juice, vegetables, milk, other juice, other sweetened drinks, sweet foods, fast food	Yes<br>Estimated mean intake of food groups, Mean ± SD [b], P<0.05<br>100% fruit juice: FI [c]: 1.34 ± 1.10, FS [d]: 1.22 ± 1.03, other juice: FI [c]: 0.51 ± 0.80, FS [d]: 0.41 ± 0.73, other sweetened drinks: FI [c]: 0.21 ± 0.55, FS [d]: 0.15 ± 0.46, sweet foods: FI [c]: 0.86 ± 0.82, FS [d]: 0.76 ± 0.76	[a] The questions had a validation against three 24-h dietary recalls and demonstrated fair to substantial agreement; [b] Standard Deviation (SD); [c] Food insecurity (FI); [d] Food security (FS); [e] In each intervention school, a cafeteria tap water dispenser with disposable cups and two reusable bottle-filling stations were installed; [f] For the Child Food-security Assessment (CFSA), students were asked how often in the previous 12 months did they experience the following: 1. We can't get the food we want because there is not enough money. 2. I worry about how hard it is for my parents to get enough food for us. 3. I worry about not having enough to eat. 4. I feel hungry, because there is not enough to eat. 5. I get really tired, because there is not enough to eat; [g] Healthy Eating Index (HEI) measures adherence to the Dietary Guidelines for Americans based on adequacy and moderation components, with higher scores indicating better diet quality; [h] Standard error (SE)
Gerstenfeld, 2024, San Francisco Bay Area, (California)	Cluster-randomized controlled trial [e], n=1056, Female, Male, 10 y	Food-insecurity was quantified using five of the nine statements from the CFSA [f]	24-hour dietary recalls were conducted at baseline and 7 months	Estimated mean plain water consumption<br>Plain water (fluid ounces)	Yes<br>Estimated mean plain water consumption (95% CI)<br>Baseline plain water intake: high FI [c]: 3.58-4.57 fluid ounces (95% CI: 2.54, 6.63), FS [d]: 6.12-7.54 fluid ounces (95% CI: 4.50, 10.48)	
Hingle 2023, Alaska, American Samoa, Commonwealth of the Northern Mariana Islands, Guam, Hawaii, the Federated States of Micronesia, the Republic of the Marshall Islands, and the Republic of Palau (Alaska and Hawai)	Cross sectional study, n = 3099, Female, Male, 2-8 y	One-item survey question adapted from the 18-item U.S. Household Food-security Survey Module (HFSSM): "In the past 12 months, how often does your money for food run out before the end of the month?"	Two dietary food records on randomly assigned nonconsecutive days following portion size training with calibrated utensils.	Estimated mean HEI [g] – 2005<br>Total score, total fruits, whole fruits, total vegetables, dark green/orange veg/ legumes, total grains, whole grains, milk, meat and beans, oils, saturated fat, sodium, calories from solid fats, alcoholic beverages, and added sugars (SoFAAS)	Yes<br>Estimated mean HEI [g] – 2005 ± SE [h], P<0.05<br>Total fruits: FI [c]: 2.3 ± 0.2, FS [d]:2.8 ± 0.2, whole fruits: FI [c]: 2.6 ± 0.2, FS [d]:3.0 ± 0.2, total vegetables: FI [c]: 1.5 ± 0.1, FS [d]:1.9 ± 0.1, dark green/orange veg/legumes: FI [c]: 0.5 ± 0.1, FS [d]: 0.8 ± 0.1, total grains: FI [c]: 4.7 ± 0.04, FS [d]: 4.6 ± 0.03, whole grains: FI [c]: 0.6 ± 0.1, FS [d]: 0.9 ± 0.2, milk: FI [c]: 4.3 ± 0.5, FS [d]: 5.4 ± 0.4, meat and beans: FI [c]: 8.9 ± 0.1, FS [d]: 8.6 ± 0.2, oils: FI [c]: 5.3 ± 0.2, FS [d]: 5.9 ± 0.1, saturated fat: FI [c]: 6.4 ± 0.2, FS [d]: 5.8 ± 0.2, sodium: FI [c]: 5.7 ± 0.2, FS [d]: 5.2 ± 0.1, SoFAAS: FI [c]: 15.6 ± 0.3, FS [d]: 15.0 ± 0.3	

Author, year, location (state)	Study design, participant characteristics	Food-security assessment method	Dietary assessment method	Dietary marker measured	Significant main findings: Is food-security associated to dietary intake?	Definition/Description
**Children**						
Jansen 2017, (Michigan)	Longitudinal study from a randomized controlled trial, n = 501, Female, Male, 4-5 y at baseline	18-item U.S. Household Food-security Survey Module (HFSSM)	Three 24-hour dietary recall on two weekdays and one weekend day were averaged	Estimated mean HEI [a] – 2010<br>HEI [a] – 2010 total score	Yes<br>Multivariate [b] analysis of the association between FS [c] and the HEI [a] – 2010 total score (95% CI), P<0.05<br>Girls who became FS [c] during the study, on average had a 9.1 points higher HEI [a] total score (95% CI: 3.1, 15.0) compared with those who remained persistently FI [d]	[a] Healthy Eating Index (HEI) measures adherence to the Dietary Guidelines for Americans based on adequacy and moderation components, with higher scores indicating better diet quality; [b] Adjusted for sociodemographic, food secure as the reference category; [c] Food security (FS); [d] Food insecurity (FI); [e] Continuing Survey of Food Intakes by individuals (CSFII): A nationally representative sample of noninstitutionalized people living in US households, provides individual estimates of food and nutrient intakes from two nonconsecutive days of 24-hour dietary recall; [f] Child Food-security Assessment (CFSA); [g] Standard Deviation (SD)
Knol 2004, CSFII [e] from the United States	Cross sectional study, n =1,242, 2-3 y, n =1,506, 4-8 y, Female, Male	Food sufficiency status was based on the household respondent's answer to the close-ended CSFII [e] question, "Which of the following statements best describes the foods eaten in the household?	Two 24-hour dietary recall were averaged	Estimated mean HEI [a]<br>HEI total score	No	
Landry, 2019, Los Angeles (California)	Cross sectional study, n = 218, Female, Male, 8-9 y	An adaption of the 5-item CFSA [f]	Block Kids Food Screener, 41-food items, last week version	Estimated mean intake of food groups<br>Meat, poultry, and fish, dairy, whole grains, fruit, vegetables, French fries, legumes, sugar sweetened beverages	Yes<br>Estimated mean intake of food groups ± SD [g]<br>French fries, frequency, FS [c]: 1.95 ± 1.29, FI [d]: 2.45 ± 1.37	
McCarthy 2023, (North Carolina)	Cross sectional study, n = 24 parent-child dyads, Female, Male, 3-5 y	2-item Hunger Vital Sign screen (HFI) assessed household food-insecurity	Three 24-hour dietary recalls on two weekdays and one weekend were averaged	Estimated mean HEI [a] –2015<br>Total fruits, whole fruits, total vegetables, greens and beans, whole grains, total dairy, total protein foods, seafood and plant proteins, fatty acids, refined grains, sodium, added sugars, saturated fat, total score	Yes<br>Multivariate [b] analysis of the association between FS [c] and the HEI [a] – 2015 total score and food components, β, P<0.05<br>Children, FI [d]: total protein: β= -1.3, added sugars: β=4.1	

Author, year, location (state)	Study design, participant characteristics	Food-security assessment method	Dietary assessment method	Dietary marker measured	Significant main findings: Is food-security associated to dietary intake?	Definition/Description
			**Children**			
Sharkey, 2012, Hidalgo County, located in the Lower Rio Grande (Texas)	Cross sectional study, n = 50, Female, Male, 6-11 y	Nine-item child food-security measure developed by Connell and colleagues	Three 24-hour dietary recalls on two weekdays and one weekend were averaged	Estimated mean intake of dietary markers<br>Energy, protein, fiber, calcium, vitamins C, D, potassium, sodium, fat (% kilocalories), added sugar (% kilocalories)	Yes<br>Multivariate [a] analysis of the association between FS [b] and dietary markers, β ± SE [c], P<0.05<br>Energy: very low FS [b]: 377.16 ± 169.91 kcal, calcium: marginal FS [b]: 270.57 ± 109.28 mg, very low FS [b]: 187.29 ± 139.25 mg, added sugar: very low FS [b]: 6.82 ± 2.05 % of kilocalories	[a] Adjusted for sociodemographic, food secure as the reference category; [b] Food security (FS); [c] Standard error (SE); [d] Children's Dietary Questionnaire (CDQ). Parents report children's intake of diet items over the past 7 days on a 7-point scale reflecting frequency of intake ranging from 'did not have' to '8+ times'; [b] The Dietary Screener Questionnaire (DSQ), a 26-item food frequency questionnaire developed by the [c] National Cancer Institute (NCI) was administered. The DSQ assesses intake on selected foods consumed over the past 30 d in number of times per day, week, or month. NCI-generated scoring algorithms were used to convert reported frequencies to estimated quantities of select food groups and nutrients, based on age- and sex-specific 24- h dietary recall portion size data from National Health and Nutrition Examination Survey (NHANES); [d] Standard Deviation (SD); [f] Food insecurity (FI)
Trapp, 2015, Greater Hartford area (Connecticut)	Cross sectional study, n = 222, Female, Male, 2-4 y	18-item U.S. Household Food-security Survey Module (HFSSM)	The CDQ [d] is a 29-item parent-reported instrument (intake of previous 24 hours and past 7 days)	Estimated mean intake of food groups<br>Fruit and vegetables, fat from dairy, sweetened beverages, noncore foods	No	

Author, year, location (state)	Study design, participant characteristics	Food-security assessment method	Dietary assessment method	Dietary marker measured	Significant main findings: Is food-security associated to dietary intake?	Definition/Description
**Children and Adolescents**						
Bahanan, 2021, NHANES [a] from the United States	Cross sectional study, n = 4822, Female, Male, 5-17 y	18-item U.S. Household Food-security Survey Module (HFSSM)	Day-1 24-hour dietary recall	Estimated mean HEI [b] – 2015<br>Total fruits, whole fruits, total vegetables, greens and beans, whole grains, dairy, total protein foods, seafood and plant proteins, fatty acids, refined grains, sodium, saturated fats, added sugars, total score	Yes<br>Estimated mean HEI [b] ± SD [c]<br>Whole fruits: FS [d]: 2.27 ± 0.08, FI [e]: 1.97 ± 0.09, seafood and plant proteins: FS [d]: 1.75 ± 0.06, FI [e]: 1.56 ± 0.06	[a] National Health and Nutrition Examination Survey (NHANES) is a continuous, cross-sectional series of surveys of nationally representative samples of the resident, civilian, non-institutionalized US population; [b] Healthy Eating Index (HEI) measures adherence to the Dietary Guidelines for Americans based on adequacy and moderation components, with higher scores indicating better diet quality; [c] Standard Deviation (SD); [d] Food security (FS); [e] Food insecurity (FI); ; [f] Standard Error (SE)
Burke, 2019, (South Carolina)	Cross sectional study, n = 171 households, 9-15 y	18-item U.S. Household Food-security Survey Module (HFSSM)	One interviewer administered 24-hour dietary recall in isolation from caregiver	Estimated mean HEI [e] – 2005<br>HEI [e] – 2005 total score	No	
Eicher-Miller, 2020, NHANES [a] from the United States	Cross sectional study, n = 8123, Female, Male, 6-11, 12-17 y	18-item U.S. Household Food-security Survey Module (HFSSM)	Day-1 24-hour dietary recall	Estimated mean percentage of food intake<br>Milk/dairy, protein, mixed dish, grain, sweet/snack, fruit, vegetable, beverage, water, fat/oil, condiment/sauce, sugars<br>Estimated mean percentage of energy from foods	Yes<br>Estimated mean percentage of food intake ± SE [f]<br>6-11y: mixed dish, FS [d]: 8.0 ± 0.2, FI [c]: 9.3 ± 0.6, snack/sweet, FS [d]: 16.1 ± 0.3, FI [e]: 14.4 ± 0.6, beverage, FS [d]: 11.4 ± 0.2, FI [e]: 12.7 ± 0.6, 12-17 y: beverage, FS [d]: 12.7 ± 0.2, FI [e]: 15.4 ± 0.6, low FS [d]: 15.5 ± 0.5, very low FS [d]: 14.4 ± 0.7, water, FS [d]: 10.3 ± 0.3, FI [e]: 8.7 ± 0.5, low FS [d]: 8.6 ± 0.6, very low FS [d]: 8.8 ± 0.5,	

Author, year, location (state)	Study design, participant characteristics	Food-security assessment method	Dietary assessment method	Dietary marker measured	Significant main findings: Is food-security associated to dietary intake?	Definition/Description
**Children and Adolescents**						
Eicher-Miller, 2020, NHANES [d] from the United States *Continued*					Estimated mean percentage of energy from foods ± SE [b] 6-11 y: snack/sweet, FS [b]: 21.0 ± 0.5, FI [c]: 18.3 ± 0.9, low FS [b]: 18.1 ± 0.9, very low FS [b]: 20.1 ± 1.8, 12-17 y: beverage, FS [b]: 11.2 ± 0.3, FI [c]: 13.0 ± 0.7	
Forrestal, 2021, SNMCS-I [a] form the United States	Cross sectional study, n = 1843, Female, Male, < 9, 9-13, > 13 y	18-item U.S. Household Food-security Survey Module (HFSSM)	24-hour dietary recall	Estimated mean percentage HEI [b]– 2010 Total fruits, whole fruits, total vegetables, greens and beans, whole grains, dairy, total protein foods, seafood and plant proteins, fatty acids, refined grains, sodium, empty calories, total score	Yes Estimated Mean percentage HEI [b] – 2005 (95% CI) Dairy, FS [c]: 80.3 (73.9, 87.5) FI [d]: 68.2 (60.4, 76.1), fatty acids, FS [c]: 33.5 (27.1, 40.3) FI [d] : 45.2 (38.5, 52.5)	[a] The first School Nutrition and Meal Cost Study (SNMCS-I), a comprehensive, national data set collected following the implementation of Healthy, Hu ger-Free Kids Act (HHFKA)-mandated changes. SNMCS-I links student-reported dietary intakes at school with school-reported menus to identify the contributions of school meals to students' diets; [b] Healthy Eating Index (HEI) measures adherence to the Dietary Guidelines for Americans based on adequacy and moderation components, with higher scores indicating better diet quality; [c] Food security (FS); [d] Food insecurity (FI);

Author, year, location (state)	Study design, participant characteristics	Food-security assessment method	Dietary assessment method	Dietary marker measured	Significant main findings: Is food-security associated to dietary intake?	Definition/Description
**Children and Adolescents**						
Jun, 2021, NHANES [a] from the United States	Cross sectional study, n = 9147, Female, Male, 1-18 y	18-item U.S. Household Food-security Survey Module (HFSSM)	Two 24-hour dietary recalls and the NCI [b] method was used to estimate nutrient usual intake	Estimated mean usual intakes of dietary markers<br>Energy, carbohydrate, added sugars, total fat, saturated fat, protein, fiber<br>Estimated mean HEI [c]–2015<br>Total fruits, whole fruits, total vegetables, greens and beans, whole grains, dairy, total protein foods, seafood and plant proteins, fatty acids, refined grains, sodium, added sugars, saturated fat, total score<br>Estimated prevalence of usual intakes <EAR[d] or >the AI [e], UL[f] and CDRR [g]<br>Vitamins A,C,D,E; folate, calcium, iron, magnesium, zinc, choline, potassium, folic acid, sodium	Yes<br>Estimated mean HEI [c]-2015 ± SE [h]<br>Whole grains, girls 2-18 y: FI [i]: 2.5 ± 0.3, FS [j]: 3.1 ± 0.1<br><br>Estimated prevalence of usual intakes <EAR [d] or >the AI [e], UL [f] and CDRR [g] ± SE [h]<br>Differences among only girls 2-18 y: vit A, FI [i]: 32.9 ± 3.5, FS [j]: 24.0 ± 2.0, vit D, FI [i]: 97.8 ± 1.1, FS [j]: 94.8 ± 0.6<br>Differences among girls and boys 2-18 y: vit E, girls: FI [i]: 80.9 ± 3.9, FS [j]: 72.8 ± 1.4, boys: FI [i]: 74.3 ± 4.4, FS [j]: 60.6 ± 1.4, calcium, girls: FI [i]: 64.0 ± 4.2, FS [j]: 52.0 ± 1.4, boys: FI [i]: 42.0 ± 4.5, FS [j]: 32.0 ± 1.7, magnesium, girls: FI [i]: 50.3 ± 3.0, FS [j]: 37.4 ± 0.9, boys: FI [i]: 34.9 ± 2.3, FS [j]: 27.4 ± 1.1, choline, girls: FI [i]: 11.7 ± 2.6, FS [j]: 18.1 ± 1.1, boys: FI[i]: 19.4 ± 2.2, FS [j]: 28.1 ± 1.2, zinc, girls: FI [i]: 7.9 ± 1.3, FS [j]: 14.0 ± 0.7, boys: FI [i]: 11.5 ± 1.7, FS [j]: 17.3 ± 0.8	[a] National Health and Nutrition Examination Survey (NHANES) is a continuous, cross-sectional series of surveys of nationally representative samples of the resident, civilian, non-institutionalized US population; [b] National Cancer Institute (NCI) method; [c] Healthy Eating Index (HEI) measures adherence to the Dietary Guidelines for Americans based on adequacy and moderation components, with higher scores indicating better diet quality; [d] Estimated Average Requirement (EAR); [e] Adequate Intake (AI); [f] Tolerable Upper Intake level (UL); [g] Chronic Disease Risk Reduction intake (CDRR); [h] Standard error (SE); [i] Food insecure (FI); [j] Food secure (FS)

Author, year, location (state)	Study design, participant characteristics	Food-security assessment method	Dietary assessment method	Dietary marker measured	Significant main findings: Is food-security associated to dietary intake?	Definition/Description
**Children and Adolescents**						
Lee, 2024, (Minnesota)	Secondary analysis of 3 randomized controlled trials, n = 404, Female, Male, 7-12 y	6-item Short Form U.S. Household Food-security Survey Module (HFSSM)	An average of two or three 24-hour dietary recalls at three times points	<u>Estimated Mean HEI [a] – 2015</u><br>Total HEI [a]– 2015 HOME plus [b], SNAPSHOT [c], NU-HOME [d], Combined [e]	Yes<br><u>Estimated Mean HEI [a] – 2015 total score</u><br>Baseline: total HEI [a]– 2015: FS [f]: 51.92 ± 11.87 FI [g]: 47.52 ± 11.87<br><u>Multivariate [h] analysis of the association between FS [f] and HEI [a] – 2015 total score, Means (95% CI), P<0.05</u><br>Combined [e], T0 to T1 [i]: 4.50 (0.99 to 8.01) T0 to T2 [j]: -1.39 (-5.64 to 2.87) SNAPSHOT [f], T0 to T1 [i]: 8.72 (2.40 to 15.04)	[a] Healthy Eating Index (HEI) measures adherence to the Dietary Guidelines for Americans based on adequacy and moderation components, with higher scores indicating better diet quality; [b] HOME Plus: A 10-month family-based program promoting nutritious family meals and reduced screen time for children; [c] SNAPSHOT: A 9-month school nurse-led weight management program for children with overweight;
Lee, 2019, Metropolitan Minnesosta (Minnesosta)	Cross sectional study, n = 218, Female, Male, 8-12 y	6-item Short Form U.S. Household Food-security Survey Module (HFSSM)	24-hour dietary recall interviews collected on weekdays and weekend days during summer months	<u>Estimated mean intake of dietary markers</u><br>HEI [a]– 2015, energy intake, vegetables, whole fruits, 100% fruit/vegetable, sugar-sweetened beverages	Yes<br><u>Multivariate [h] analysis of the association between FS [f] and dietary markers, Means (95% CI), P<0.05</u><br>Energy intake: FS [f]: 1703.87 (1606.87–1800.88), FI [g]: 1377.75 (1214.89–1540.62), whole fruits: FS [f]: 0.29 (0.23–0.36), FI [g]: 0.16 (0.05–0.27), sugar-sweetened beverages: FS [f]: 0.43 (0.31–0.55), FI [g]: 0.73 (0.53–0.93)	[d] NUHOME: An adaptation of HOME Plus; [e] Combined data: combination of HOME plus, SNAPSHOT, and NU-HOME; [f] Food security (FS); [g] Food insecurity (FI); [h] Adjusted for sociodemographic, food secure as the reference category; [i] The time points between baseline before randomization (T0) and postintervention (T1); [j] The time points between baseline before randomization (T0) and follow-up (T2)
Potochnick, 2019, Bronx (Illinois), Chicago (Illinois), Miami (Florida,) San Diego (California)	Cross sectional study, n = 1362, Female, Male, 8-16 y	18-item U.S. Household Food-security Survey Module (HFSSM)	Not reported	<u>Estimated Mean HEI [a] – 2010</u><br>Total HEI [a] – 2010	No	

Author, year, location (state)	Study design, participant characteristics	Food-security assessment method	Dietary assessment method	Dietary marker measured	Significant main findings: Is food-security associated to dietary intake?	Definition/Description
**Children and Adolescents**						
Rossen, 2016, NHANES [a] from the United States	Cross sectional study, n = 5136, Female, Male, 2-15 y	18-item U.S. Household Food-security Survey Module (HFSSM)	At least one 24-hour dietary recall with a trained interviewer and a second recall conducted for most participants by telephone 3-10 days later. The NCI [b] method was used to estimate nutrient usual intake	Estimated mean intake of food groups<br>Fruit juice, whole fruit, vegetables excluding potatoes, starchy vegetables, whole grains, refined grains, solid fats, added sugar, Solid Fats and Added Sugars (SoFAS), kilocalories, protein, carbohydrate, fiber, caloric density (kcal/food item), sodium, dietary variety (# of foods)	No	[a] National Health and Nutrition Examination Survey (NHANES) is a continuous, cross-sectional series of surveys of nationally representative samples of the resident, civilian, non-institutionalized US population; [b] National Cancer Institute; [c] The adult respondent answered the child-level survey questions. Twenty-four of the 26 items from the 2009-2010 National Health and Nutrition Examination Survey Multifactor Diet Screener (DSQ) were used to assess intake of select dietary factors in children during the previous 30 days. The DSQ has been cognitively tested and many of the items included have undergone validity testing; [d] Adjusted for sociodemographic, food secure as the reference category; [e] Food security (FS)
Soldavini, 2021, Cherokee Nation (Oklahoma), Chickasaw Nation (Oklahoma), (Connecticut), (Delaware), (Michigan), (Missouri),	Cross sectional study, n = 11,873, Female, Male, 3-4, 5-8, 9-12, 13-17 y	18-item U.S. Household Food-security Survey Module (HFSSM)	Twenty-four of the 26 items from the 2009-2010 DSQ [c] from the NHANES [a]	Estimated mean intake of food groups<br>Fruits and vegetables, fruits and vegetables excluding fried potatoes, whole grains, added sugars, added sugar excluding cereal, added sugar from sugar sweetened beverages, dairy products	Yes<br>Multivariate [d] analysis of the association between FS [e] and mean intake of food groups, Means (95% CI)<br>Fruits and vegetables (c equivalents/d), 3-4 y: high FS [e]: 2.61 (2.24 to 2.98) marginal FS [e]: 1.82 (1.20 to 2.43) low FS [e]: 1.86 (1.38 to 2.34) very-low FS [e]: 1.62 (0.98 to 2.26), 5-8 y: high FS [e]: 2.99 (2.86 to 3.12) marginal FS [e]: 2.79 (2.63 to 2.95) low FS [e]: 2.56 (2.41 to 2.71) very-low FS [e]: 2.26 (2.05 to 2.47), 9-12 y: high FS [e]: 2.89 (2.73 to 3.05) low FS [e]: 2.48 (2.33 to 2.63) very-low FS [e]: 2.10 (1.89 to 2.30),	

Author, year, location (state)	Study design, participant characteristics	Food-security assessment method	Dietary assessment method	Dietary marker measured	Significant main findings: Is food-security associated to dietary intake?	Definition/Description
**Children and Adolescents**						
Soldavini, 2021, (Nevada), (Oregon), (Texas), (Washington) *Continued*					13-17 y: high FS [e]: 3.17 (2.99 to 3.35) marginal FS [e]: 2.93 (2.74 to 3.12) low FS [e]: 2.61 (2.47 to 2.75) very-low FS [e]: 2.24 (2.05 to 2.43), fruits and vegetables excluding fried potatoes (c equivalents/d), 3-4 y: high FS [e]: 2.51 (2.15 to 2.86) marginal FS [e]: 1.76 (1.18 to 2.34) low FS [e]: 1.80 (1.35 to 2.26) very-low FS [e]: 1.58 (0.97 to 2.20), 5-8 y: high FS [e]: 2.88 (2.75 to 3.00) marginal FS [e]: 2.68 (2.52 to 2.83) low FS [e]: 2.46 (2.31 to 2.60) very-low FS [e]: 2.15 (1.94 to 2.35), 9-12 y: high FS [e]: 2.77 (2.61 to 2.92) low FS [e]: 2.37 (2.22 to 2.51) very-low FS [e]: 1.98 (1.78 to 2.18), 13-17 y: high FS [e]: 3.02 (2.84 to 3.19) marginal FS [e]: 2.77 (2.58 to 2.96) low FS [e]: 2.47 (2.33 to 2.60), very-low FS [e]: 2.07 (1.89 to 2.25), whole grains (oz equivalents/d), 5-8 y: high FS [e]: 1.40 (1.19 to 1.61) low FS [e]: 1.24 (1.05 to 1.44),	

Author, year, location (state)	Study design, participant characteristics	Food-security assessment method	Dietary assessment method	Dietary marker measured	Significant main findings: Is food-security associated to dietary intake?	Definition/Description
**Children and Adolescents**						
Soldavini, 2021, Cherokee Nation (Oklahoma), Chickasaw Nation (Oklahoma), (Connecticut), (Delaware), (Michigan), (Missouri), (Nevada), (Oregon), (Texas), (Washington) *Continued*					9-12 y: high FS [e]: 1.46 (1.23 to 1.69) marginal FS [e]: 1.88 (1.47 to 2.28), 13-17 y: high FS [e]: 1.92 (1.58 to 2.25) very-low FS [e]: 1.49 (1.03 to 1.96), added sugar, 3-4y: high FS [e]: 14.59 (12.00 to 17.19) low FS [e]: 11.74 (9.25 to 14.24), 5-8 y: high FS [e]: 14.70 (13.87 to 15.53) marginal FS [e]: 15.67 (14.65 to 16.69), 9-12 y: high FS [e]: 18.35 (17.39 to 19.31) very-low FS [e]: 16.60 (15.17 to 18.03), 13-17 y: high FS [e]: 22.07 (20.86 to 23.27) very low FS [e]: 19.68 (18.08 to 21.28), added sugar excluding cereal (tsp/d), 3-4 y: high FS [e]: 14.18 (11.76 to 16.60) low FS [e]: 11.65 (9.29 to 14.01), 5-8 y: high FS [e]: 14.12 (13.36 to 14.88) marginal FS [e]: 15.06 (14.12 to 16.00), 9-12 y: high FS [e]: 17.35 (16.53 to 18.17) very-low FS [e]: 15.87 (14.49 to 17.25) 13-17 y: high FS [e]: 20.53 (19.48 to 21.57) very-low FS [e]: 18.36 (16.97 to 19.75), added sugar from sugar-sweetened beverages (tsp/d), 3-4 y: high FS [e]: 5.40 (2.88 to 7.93)	

Author, year, location (state)	Study design, participant characteristics	Food-security assessment method	Dietary assessment method	Dietary marker measured	Significant main findings: Is food-security associated to dietary intake?	Definition/Description
**Children and Adolescents**						
Soldavini, 2021, Cherokee Nation (Oklahoma), Chickasaw Nation (Oklahoma), (Connecticut), (Delaware), (Michigan), (Missouri), (Nevada), (Oregon), (Texas), (Washington) *Continued*					low FS [e]: 3.56 (1.27 to 5.85, 5-8 y: high FS [e]: 4.86 (4.15 to 5.57) marginal FS [e]: 5.90 (4.89 to 6.90), dairy products (c equivalents/d), 3-4 y: high FS [e]: 2.16 (1.80 to 2.51) very-low FS [e]: 1.48 (1.00 to 1.96), 5-8 y: high FS [e]: 2.10 (1.97 to 2.23) low FS [e]: 1.88 (1.75 to 2.01) very-low FS [e]: 1.61 (1.43 to 1.78), 9-12 y: high FS [e]: 2.21 (2.08 to 2.35) low FS [e]: 1.95 (1.82 to 2.07) very-low FS [e]: 1.69 (1.53 to 1.85), 13-17 y: high FS [e]: 2.58 (2.42 to 2.73) low FS [e]: 2.25 (2.11 to 2.40) very-low FS [e]: 1.95 (1.74 to 2.16)<br>Multivariate [c] analysis of the association between FS [e] and mean intake of food groups, β (95% CI)<br>Fruits and vegetables (c equivalents/d), 3-4 y: marginal FS [e]: -0.80 (-1.36 to -0.24) low FS [e]: -0.76 (-1.21 to -0.30) very-low FS [e]: -0.99 (-1.59 to -0.39) 5-8 y: marginal FS [e]: -0.20 (-0.36 to -0.05) low FS [e]: -0.43 (-0.56 to -0.30) very-low FS [e]: -0.73 (-0.93 to -0.53), 9-12 y: low FS [e]: -0.41 (-0.57 to -0.25) very-low FS [e]: -0.79 (-1.01 to -0.58) 13-17 y: marginal FS [e]: -0.24 (-0.45 to -0.03) low FS [e]: -0.56 (-0.74 to -0.38)	

Author, year, location (state)	Study design, participant characteristics	Food-security assessment method	Dietary assessment method	Dietary marker measured	Significant main findings: Is food-security associated to dietary intake?	Definition/Description
**Children and Adolescents**						
Soldavini, 2021, Cherokee Nation (Oklahoma), Chickasaw Nation (Oklahoma), (Connecticut), (Delaware), (Michigan), (Missouri), (Nevada), (Oregon), (Texas), (Washington) *Continued*					very-low FS [e]: -0.93 (-1.14 to -0.71), fruits and vegetables excluding fried potatoes (c equivalents/d), 3-4 y: marginal FS [e]: -0.74 (-1.27 to -0.22) low FS [e]: -0.71 (-1.13 to -0.28) very-low FS [e]: -0.92 (-1.50 to -0.34), 5-8 y: marginal FS [e]: -0.20 (-0.35 to -0.05) low FS [e]: -0.42 (-0.54 to -0.30) very-low FS [e]: -0.73 (-0.92 to -0.53), 9-12 y: low FS [e]: -0.40 (-0.56 to -0.24) very-low FS [e]: -0.79 (-1.00 to -0.58), 13-17 y: marginal FS [e]: -0.25 (-0.45 to -0.05) low FS [e]: -0.55 (-0.72 to -0.38) very-low FS [e]: -0.94 (-1.14 to -0.74), whole grains (oz equivalents/d), 5-8 y: low FS [e]: -0.16 (-0.31 to -0.00), 9-12 y: marginal FS [e]: 0.42 (0.01 to 0.83), 13-17 y: very-low FS [e]: -0.42 (-0.83 to -0.01), added sugar (tsp/d): 3-4 y: low FS [e]: -2.85 (-4.50 to -1.20), 5-8 y: marginal FS [e]: .97 (0.03 to 1.91), 9-12 y: very-low FS [e]: -1.75 (-3.25 to -0.25), 13-17 y: very-low FS [e]: -2.39 (-4.03 to -0.75), added sugar excluding cereal (tsp/d), 3-4 y: low FS [e]: -2.53 (-4.06 to -1.01), 5-8 y: marginal FS [e]: 0.94 (0.10 to 1.77), 9-12 y: very-low FS [e]: -1.48 (-2.90 to -0.06),	

Author, year, location (state)	Study design, participant characteristics	Food-security assessment method	Dietary assessment method	Dietary marker measured	Significant main findings: Is food-security associated to dietary intake?	Definition/Description
**Children and Adolescents**						
Soldavini, 2021, Cherokee Nation (Oklahoma), Chickasaw Nation (Oklahoma), (Connecticut), (Delaware), (Michigan), (Missouri), (Nevada), (Oregon), (Texas), (Washington) *Continued*					13-17 y: very-low FS [e]: -2.17 (-3.56 to -0.78), added sugar from sugar-sweetened beverages (tsp/d), 3-4 y: -1.84 (-3.15 to -0.54), 5-8 y: marginal FS [e]: 1.04 (0.09 to 1.99), dairy (c equivalents/d), 3-4 y: very-low FS [e]: -0.68 (-1.07 to -0.29), 5-8 y: low FS [e]: -0.22 (-0.32 to -0.12) very-low FS [e]: -0.49 (-0.65 to -0.34), 9-12 y: low FS [e]: -0.26 (-0.38 to -0.15) very-low FS [e]: -0.52 (-0.68 to -0.36), 13-17 y: low FS [e]: -0.32 (-0.46 to -0.18) very-low FS [e]: -0.63 (-0.82 to -0.44)	
Trude, 2024, Baltimore City (Maryland)	Cross sectional study, n = 451, Female, Male, 9-15 y	18-item U.S. Household Food-security Survey Module (HFSSM)	Block Kids 2004 Food Frequency Questionnaire (BKFFQ) [a]	Estimated mean intake of food groups<br>Energy, carbohydrate, protein, fat, fiber, added sugar, vitamin B-12, vitamin D, calcium, iron, zinc, sodium<br>Estimated prevalence of intakes <EAR [b] or >the AI [c]<br>Energy, carbohydrate, protein, fat, fiber, added sugar, vitamin B-12, vitamin D, calcium, iron, zinc, sodium	Yes<br>Estimated mean intake of food groups ± SD [d]<br>Energy, FS [e]: 1722.9 ± 1018.2, FI [f]: 1947.3 ± 1133.3, girls: FS [e]: 1653.9 ± 74.9, FI [f]: 2201.0 ± 139.4, boys: FS [e]: 1801.0 ± 79.7, FI [f]: 1580.1 ± 167.7, protein, FS [e]: 13.0 ± 2.1, FI [f]: 12.5 ± 2.1, fiber, girls: FS [e]: 13.9 ± 0.7, FI [f]: 19.5 ± 1.3, boys: FS [e]: 14.5 ± 0.7, FI [f]: 12.1 ± 1.5, added sugar, FS [e]: 17.1 ± 12.3, FI [f]: 21.1 ± 14.9, vitamin B12, girls: FS [e]: 3.4 ± 0.1, FI [f]: 3.2 ± 0.1, boys: FS [e]: 3.6 ± 0.1, FI [f]: 3.6 ± 0.1, calcium, girls: FS [e]: 655.1 ± 11.1, FI [f]: 641.4 ± 30.8, boys: FS [e]: 713.8 ± 14.7, FI [f]: 712.4 ± 32.4	[a] The Block Kids 2004 Food Frequency Questionnaire (BKFFQ) is a semi-quantitative, validated questionnaire in adolescent populations that ascertains previous week's frequency and consumption amount of 77 common food items (with three to four categories related to food type). It contains foods identified by NHANES II commonly consumed by youth; [b] Estimated Average Requirement (EAR); [c] Adequate Intake (AI); [d] Standard Deviation (SD); [e] Food security (FS); [f] Food insecurity (FI)

Author, year, location (state)	Study design, participant characteristics	Food-security assessment method	Dietary assessment method	Dietary marker measured	Significant main findings: Is food-security associated to dietary intake?	Definition/Description
**Children and Adolescents**						
Trude, 2024, Baltimore City (Maryland) *Continued*					Estimated prevalence of intakes <EAR[b] or >the AI [c], % (n)<br>Fiber, FS [e]: 8.1 (29), FI [f]: 13.9 (13), girls: FS [e]: 8.5 (16), FI [f]: 23.6 (13), boys: FS [e]: 7.7 (13), FI [f]: 0 (0), vitamin B12, girls: FS [e]: 82.6 (157), FI [f]: 90.9 (50), boys: FS [e]: 88.9 (64), FI [f]: 84.2 (48), calcium, girls: FS [e]: 9.5 (18), FI [f]: 21.8 (12), boys: FS [e]: 16.6 (28), FI [f]: 7.9 (3)	
**Adolescents**						
Hammad, 2024, NHANES [a] from the United States	Cross sectional study, n = 2534, Female, Male, 15 y	18-item U.S. Household Food-security Survey Module (HFSSM)	Two 24-hour dietary recalls were averaged to estimate the diet component of the LE8 [b] and HEI [c] –2015 components and total score	Estimated mean LE8 [b]<br>Diet component<br><br>Estimated mean HEI [c] – 2015<br>Total fruit, whole fruit, total vegetables, greens and beans, whole grains, dairy, total protein foods, seafood and plant proteins, fatty acid ratio, sodium, refined grains, saturated fat, added sugar, total score	Yes<br>Multivariate [d] analysis of the association between FS [e] and LE8 [b], β (95% CI), P<0.05<br>Diet component of the LE8 [b]<br>FI [e]: β= -5.4 (−8.9, −1.9)<br>Multivariate [d] analysis of the association between FS [e] and HEI [c] – 2015 total score and food components, Means ± SE [g] (95% CI), P<0.05<br>Whole grains: FS [g]: 3.1 ± 0.1, FI [h]: 2.3 ± 0.1, RD [f]: 0.81 (0.70, 0.94), seafood and plant proteins: FS [g]: 2.0 ± 0.1, FI [h], mean scores: 1.8 ± 0.1, RD [h]: 0.82, CI: 0.71, 0.95, HEI – 2015 total score: FS [g]: 47.4 ± 0.5, FI [h]: 45.2 ± 0.5, RD [h]: 0.95 (0.93, 0.98)	[a] National Health and Nutrition Examination Survey (NHANES) is a continuous, cross-sectional series of surveys of nationally representative samples of the resident, civilian, non-institutionalized US population; [b] The Life's Essential 8 (LE8) measures cardiovascular health including healthy diet; [c] Healthy Eating Index (HEI) measures adherence to the Dietary Guidelines for Americans based on adequacy and moderation components, with higher scores indicating better diet quality; [d] Adjusted for sociodemographic, food secure as the reference category; [e] FS: Food security (FS); [f] Risk difference (RD); [g] Standard Error (SE); [h] FI: Food insecurity (FI)

Author, year, location (state)	Study design, participant characteristics	Food-security assessment method	Dietary assessment method	Dietary marker measured	Significant main findings: Is food-security associated to dietary intake?	Definition/Description
**Adolescents**						
Marshall, 2021, (District of Columbia and Tennessee)	Cross sectional study, n = 627, Female, Male, 14 y	Two- question screening questionnaire [a] from the 18-item U.S. Household Food-security Survey Module (HFSSM)	Open-ended questions were used to query daily meal and snack frequency. Daily beverage intake was calculated from a validated questionnaire asking if the beverage was consumed, weekly frequency of consumption, and quantity consumed at each occurrence	Estimated medians of beverage intakes<br>Daily beverage intakes (oz): milk, 100% juice, sugar-sweetened beverages, water and other sugar-free beverages, total beverage intake	Yes<br>Estimated medians of beverage intakes (25th, 75th percentile), P<0.05<br>Water & other sugar- free beverages, FS [b]: 20.0 oz. (11.4, 45.7), FI [c]: 16.0 oz. (8.6, 31.4)	[a] Two-question screening questionnaire from the 18-item U.S. Household Food-security Survey Module (HFSSM) validated in caregivers of low-income children and in teenagers and young adults. Adolescents were asked both screening questions: “We worried whether our food would run out before we got money to buy more” and “The food that we bought just didn’t last and we didn’t have money to get more.” If adolescents responded “often true” or “sometimes true” to either question, they were classified as experiencing food-insecurity; [b] Food security (FS); [c] Food insecurity (FI); [d] National Health and Nutrition Examination Survey (NHANES) is a continuous, cross-sectional series of surveys of nationally representative samples of the resident, civilian, non-institutionalized US population; [e] National Cancer Institute (NCI) method; [f] Standard Error (SE)
Morales-Juárez, 2024, NHANES [d] from the United States	Cross sectional study, n = 3633, Female, Male, 14-17 y	18-item U.S. Household Food-security Survey Module (HFSSM)	Two 24-hour dietary recalls and the NCI [e] method was used to estimate nutrient usual intake	Estimated mean usual nutrient intake<br>Lutein+ zeaxanthin, choline, vitamin A, potassium, calcium, selenium, magnesium, vitamin D, iron, zinc, vitamin E, vitamin B12, vitamin B2, vitamin B1, vitamin B6, DHA, Vitamin C, vitamin B3, protein DGA	Yes<br>Estimated mean usual nutrient intake by food security status and egg-rich diets, means ± SE [f], P<0.0002<br>Lutein + Zeaxanthin: Non-Egg consumers: FI [c], 886.8 ± 119.6, Eggs as Ingredients in dishes: FS [b], 1084.7±50.7, Primarily egg dishes: FS [b], 1544.1 ± 87.6, choline: Non-Egg consumers: FI [c], 217.4 ± 38.8, FS [b], 268.5±10.0, Eggs as Ingredients in dishes: FI [c], 296.4±12.0, FS [b], 295.0 ± 7.9, Primarily egg dishes: FS [b], 408.4 ± 17.6, selenium, Non-Egg consumers: FS [b], 100.1 ± 3.8, Primarily egg dishes: FS [b], 128.6 ± 3.9, Vitamin D, Non-Egg consumers: FI [c], 3.6 ± 0.4, Primarily egg dishes: FS [b], 6.0 ± 0.2, vitamin B2, Non-Egg consumers: FS [b], 1.9 ± 0.0, Primarily egg dishes: FS [b], 2.3 ± 0.0,	

Author, year, location (state)	Study design, participant characteristics	Food-security assessment method	Dietary assessment method	Dietary marker measured	Significant main findings: Is food-security associated to dietary intake?	Definition/Description
			**Adolescents**			
Morales-Juárez, 2024, NHANES [a] from the United States *Continued*					DHA, Non-Egg consumers: FI [c], 30.0 ± 0.0, FS [b], 30.0 ± 0.0, Eggs as Ingredients in dishes: FI [c], 30.0 ± 0, FS [b], 40.0 ± 0, Primarily egg dishes: FS [b], 70.0 ± 0, protein DGA, Non-Egg consumers: FS [b], 72.8 ± 2.6, Primarily egg dishes: FS [d], 89.1±2.3	
			**Children; Adolescents; Adults**			
Eicher-Miller, 2011, NHANES [a] from the United States	Cross sectional study, n = 5270, Female, Male, Adults; Children, Adolescents, Adult, 8-11, 12-15, 16-19 y	18-item U.S. Household Food-security Survey Module (HFSSM)	One 24-hour dietary recall was recorded for the food and nutrient intake	Estimated mean calcium-related dietary factors<br>Calcium intake <EAR [b] and servings of dairy < recommended	Yes<br>Multivariate [c] analysis of the association between FS [d] and calcium-related dietary factors, OR, (95% CI) P≤0.05<br>Males 8–11 y, FI [f]: calcium intake < EAR [b]: 2.3 (1.3–4.0), servings of dairy < recommended: 2.5 (1.1–5.8)	[a] National Health and Nutrition Examination Survey (NHANES) is a continuous, cross-sectional series of surveys of nationally representative samples of the resident, civilian, non-institutionalized US population; [b] Estimated average requirement (EAR), defined by sex and age; [c] Adjusted for sociodemographic, food secure as the reference category; [d] Food secure (FI); [e] Odds ratios (OR); [f] Food insecure (FI)
			**Children; Adults**			
Tomayko, 2017, from four rural and one urban communities [a] in the United States	Cross sectional study, n = 450, Female, Male, adult caregiver-child dyad, 31.5 ± 8.5 y, 2–5 y	Two items [b] from the 18-item U.S. Household Food-security Survey Module (HFSSM)	A diet screener based on the Dietary Screener Questionnaire used in the National Health and Nutrition Examination Survey (2009–2010) was used for adults, and the validated child dietary screener based on questions contained in the	Estimated median intake of food groups<br>Fruit, vegetables, salad, potatoes, fried potatoes, pizza, 100% juice, soda, other sugar sweetened beverages (e.g., lemonade, sweetened tea, fruit punch , Kool-Aid),	Yes<br>Estimated median intake of food groups and interquartile range (IQR), P<0.05<br>Child: salad: FS [c]: 0.29 (0-0.29), FI [d]: 0.29 (0-0.71), fried potatoes: FS [c]: 0.29 (0.29-0.29), FI [d]: 0.29 (0.29-0.29), soda: FS [c]: 0 (0-0.29), FI [d]: 0.29 (0-0.29), Adult: vegetables: FS [c]: 0.71	[a] As part of the Healthy Children, Strong Families, a healthy lifestyle intervention for American Indian families with young children; [b] The two questions were as follows: "Within the past 12 months we were worried

Author, year, location (state)	Study design, participant characteristics	Food-security assessment method	Dietary assessment method	Dietary marker measured	Significant main findings: Is food-security associated to dietary intake?	Definition/Description
**Children; Adults**						
Tomayko, 2017, from four rural and one urban communities [a] in the United States *Continued*			2010 National Youth Physical Activity and Nutrition Survey was used for children. Questions asked about intake over the previous 7 days	and milk	(0.29-1), FI [d]: 0.29 (0.29-0.71), fried potatoes: FS [c]:0.29 (0.29-0.29), FI [d]: 0.29 (0.29-0.71), 100% juice: FS [c]:0.29 (0-0.71), FI [d]: 0.29 (0.29-0.71), other sugar-sweetened beverages: FS [c]:0.29 (0-1), FI [d]: 0.71 (0.29-1)	whether our food would run out; before we got money to buy more" and "Within the past 12 months the food we bought just didn't last and we didn't have money to get more". An affirmative answer to either of these two questions indicates food-insecurity with 97% sensitivity and 83% specificity; [c] Food security (FS); [d] Food insecurity (FI)
**Adolescents; Adult**						
Larson, 2020, Minneapolis–St Paul (Minnesota)	Population-based, longitudinal study, n = 1518, Female, Male, 12-14 y at EAT [a] 2010 and 20-22 y at EAT [a] 2018	Two items [b] from the 18-item U.S. Household Food-security Survey Module (HFSSM).	Food frequency questionnaire (FFQ)	Estimated mean intake of food groups<br>Fruit and vegetables, whole fruit (excluding juice), fruit juice, vegetables (excluding potatoes), dark green vegetables, red and orange vegetables, dairy, whole grains, sugar-sweetened drinks, potassium, vitamin D, calcium, iron, fiber, added sugars, sodium, and saturated fat	Yes<br>Multivariate [c] analysis of the association between FS [d] and mean intake of food groups, means, P<0.05<br>Fruit and vegetables: FS [d]: 2.2, FI [e]: 1.7, whole fruit (excluding juice): FS [d]: 0.8, FI [e]: 0.7, vegetables (excluding potatoes): FS [d]: 1.4, FI [e]: 1.1, dark green vegetables: FS [d]: 0.3. FI [e]: 0.2, red and orange vegetables: FS [d]: 0.3, FI [e]: 0.2, whole grains, mean: FS [d]: 1.1, FI [e]: 0.9, sugar-sweetened drinks, mean: FS [d]: 0.3, FI [e]: 0.4, potassium, mg: FS [d]: 1505, FI [e]: 1386, vitamin D, mg/1000 kcal: FS [d]: 104, FI [e]: 94, calcium: FS [d]: 452, FI [e]: 424, fiber: FS [d]: 11.2, FI [e]: 9.9, added sugars: FS [d]: 28.9, FI [e]: 33.5, saturated fat as % of total energy: FS [d]: 10.6, FI [e]: 11.1	[a] Eating and Activity over Time 2010–2018 (EAT) is a population-based, longitudinal study of weight-related health behaviors and associated factors; [b] If participants responded "yes" when asked "did you ever eat less than you felt you should" and also "yes" when asked "were you ever hungry but didn't eat" because "there was not enough money for food" in the past year, they were categorized as food insecure; [c] Adjusted for sociodemographic, food secure as the reference category; [d] Food secure (FS); [e] Food insecure (FI)

Author, year, location (state)	Study design, participant characteristics	Food-security assessment method	Dietary assessment method	Dietary marker measured	Significant main findings: Is food-security associated to dietary intake?	Definition/Description
**Adults**						
Allen, 2016, Baltimore City (Maryland)	Cohort study, n = 1,741, Female, Male, 38-57 y	Food-security Module (FSM), a modified version of the USDA's [a]	Two 24-hour dietary recall were averaged	Estimated mean HEI [b] –2010<br>Total HEI [b] –2010	Yes<br>Multivariate [c] analysis of the association between FS [d] and HEI [b] –2010 total score, β ±SE [e], P<0.05<br>β= -1.33 ± 0.26	[a] Food-insecurity was measured using a modified version of the USDA's Food-security Module (FSM) administered through Audio Computer-Assisted Self-Interviewing (ACASI); [b] Healthy Eating Index (HEI) measures adherence to the Dietary Guidelines for Americans based on adequacy and moderation components, with higher scores indicating better diet quality; [c] Adjusted for sociodemographic, food secure as the reference category; [d] Food security (FS); [e] Standard error (SE); [f] The Automated Self-Administered 24-hour Dietary Recall (ASA24™), an internet-based 24-hour dietary recall, with optional staff assistance; [g] Food insecurity (FI); [h] National Health and Nutrition Examination Survey (NHANES) is a continuous, cross-sectional series of surveys of nationally representative samples of the resident, civilian, non-institutionalized US population; [i] National Cancer Institute (NCI) method; [j] Estimated Average Requirement (EAR); [k] Adequate Intake (AI)
Anderson, 2023, Eastern Massachusetts (Massachusetts)	Cross sectional study, n = 846, Female, Male, 41 y	10-item U.S. Household Food-security Survey Model (HFSSM) Adult Food-security Survey module	Two ASA24s [f] at least 24 hours apart were averaged	Estimated Mean HEI [b] – 2015	No	
Butler, 2024, Central North Carolina (North Carolina)	Cross sectional study, n = 100, Female, 26-49 y	10-item U.S. Household Food-security Survey Model (HFSSM) Adult Food-security Survey module	Two 24-hour dietary recall were averaged on nonconsecutive days, including one weekday and one weekend day, over a two-week period	Estimated mean energy intake and diet quality<br>Total energy intake and total HEI [b] –2010	Yes<br>Estimated mean HEI [b] –2010 ± SE [e], P<0.05<br>Total HEI [b]: –2010: FS [d]: 54.4 ± 17.3, FI [g]: 46.8 ± 16.7	
Conrad, 2018, NHANES [h] from the United States	Cross sectional study, n = 34,741, Female, Male, 26-49 y	18-item U.S. Household Food-security Survey Module (HFSSM)	Two 24-hour dietary recalls and the NCI [i] method was used to estimate nutrient usual intake	Estimated mean usual intakes of dietary markers<br>Protein, fiber, eicosapentaenoic acid, docosahexaenoic acid, α-linolenic acid, vitamin K, choline, potassium, α-carotene, β-carotene, β-cryptoxanthin, lutein and zeaxanthin<br>Estimated prevalence of usual intakes <EAR [j] or >the AI [k]<br>Saturated fatty acids, vitamins A,C,D,E; thiamin riboflavin, vitamin B-6, vitamin B-12, calcium, iron, magnesium, sodium, zinc	Yes<br>Estimated mean usual intakes of dietary markers, P<0.05<br>Protein, FI [g]: 81 (79–83), FS [d]: 84 (83-84), fiber, FI [g]: 14 (13-14), FS [d]: 16 (16-16), eicosapentaenoic acid, FI [g]: 0.02 (0.02-0.03), FS [d]: 0.03 (0.03-0.03), docosahexaenoic acid, FI [g]: 0.07 (0.06-0.08), FS [d]: 0.08 (0.08-0.08), α-linolenic acid, FI [g]: 1.40 (1.35-1.44), FS [d]: 1.53 (1.5-1.55), vit K, FI [g]: 76 (72-79), FS [d]: 100 (97-103), choline, FI [g]: 291 (282-300),	

Author, year, location (state)	Study design, participant characteristics	Food-security assessment method	Dietary assessment method	Dietary marker measured	Significant main findings: Is food-security associated to dietary intake?	Definition/Description
**Adults**						
Conrad, 2018, NHANES [a] from the United States *Continued*					FS [d]: 317 (312-322), potassium, FI [g]: 2586 (2555-2618), FS [d]: 2222 (2159-2286), α-carotene, FI [g]: 320 (292-349), FS [d]: 516 (488-544), β-carotene, FI [g]: 1417 (1331-1504), FS [d]: 2136 (2054-2218), β-cryptoxanthin, FI [g]: 84 (78-90), FS [d]: 112 (108-116), lutein and zeaxanthin, FI [g]: 998 (938-1058), FS [d]: 1418 (1362-1473)<br>Estimated prevalence of usual intakes <EAR[c] or >the AI [d], $P<0.05$<br>Saturated fatty acids, FI [g]: 55.3 (52.1–58.5) FS [d]: 62.1 (60.6–63.6), vit A, FI [g]: 67.5 (64.7-70.2) FS [d]: 46.9 (45.4–48.4), vit C, FI [g]: 58.7 (55.9–61.4) FS [d]: 45.9 (44.3–47.5), vit D, FI [g]: 98 (97.8–98.2) FS [d]: 95 (94.8–95.2), vit E, FI [g]: 94.9 (93.9–95.8) FS [d]: 88.1 (87.0–89.2), thiamin, FI [g]: 12 (10.5–13.6) FS [d]: 9.5 (8.8–10.2), riboflavin, FI [g]: 12.5 (10.9–14.0), FS [d]: 9.7 (9.0–10.4), vit B6, FI [g]: 22.6 (20.5–24.6) FS [d]: 18.8 (17.6–19.9), Vit B12, FI [g]: 13.6 (12.0–15.3) FS [d]: 10.8 (10.0–11.6), calcium, FI [g]: 54.7 (52.0–57.4) FS [d]: 47.4 (46.2–48.6), iron, FI [g]: 15.7 (14.1–17.4) FS [d]: 12.7 (11.9–13.5), magnesium, FI [g]: 83.1 (81.3–84.9) FS [d]: 72.1 (70.9–73.3), sodium, FI [g]: 78.7 (76.9–80.5 FS [f]: 81.4 (80.5–82.3), zinc, FI [g]: 47 (44.7–49.3) FS [f]: 39.4 (38.2–40.6)	

Author, year, location (state)	Study design, participant characteristics	Food-security assessment method	Dietary assessment method	Dietary marker measured	Significant main findings: Is food-security associated to dietary intake?	Definition/Description
**Adults**						
Cowan-Pyle, 2024, NHANES [a] from the United States	Cross sectional study, n = 1,040, Female, Male, 18-23 y	18-item U.S. Household Food-security Survey Module (HFSSM)	Two 24-hour dietary recalls were approximated using the NCI [b] method	Estimated mean usual intakes for HEI [c]–2015<br>Total score, total fruits, whole fruits, total vegetables, greens and beans, whole grains, dairy, total protein foods, seafood and plant protein, fatty acids, refined grains, sodium, added sugars, saturated fats, total energy	Yes<br>Estimated mean usual intakes for HEI [c]–2015 (SE [d]), P<0.05<br>Added sugars, FI [e]: 5.9 (0.5), FS [f]: 7.0 (0.2)	[a] National Health and Nutrition Examination Survey (NHANES) is a continuous, cross-sectional series of surveys of nationally representative samples of the resident, civilian, non-institutionalized US population; [b] National Cancer Institute (NCI) method; [c] Healthy Eating Index (HEI) measures adherence to the Dietary Guidelines for Americans based on adequacy and moderation; [d] Standard Error (SE); [e] Food insecurity (FI); [f] Food security (FS); components, with higher scores indicating better diet quality; [g] Fruits and vegetables (FV); [h] Standard Deviation (SD)
Daniels, 2021, A land-grant university in the Midwest region of the U.S.	Cross sectional study, n = 37, Female, Male, 18-33 y	6-item Short Form U.S. Household Food-security Survey Module (HFSSM)	Food Frequency Questionnaire (FFQ) over six months as a measure of usual dietary intake	Estimated mean intake of dietary markers<br>Added sugar, calcium, fiber, folate, iron, saturated fat, sodium, vitamins A, B12, C, E, whole grains, fruit, vegetable	No	
El Zein, 2020, University of Florida (Florida), Auburn University (Alabama), South Dakota State University (South Dakota), University of Maine (Maine), West Virginia University (West Virginia), Kansas State University (Kansas), Syracuse University (New York), and University of Tennesse (Tennesse)	Cross sectional study, n = 683, Female, Male, 18-19, 20-21 y	10-item U.S. Household Food-security Survey Model (HFSSM) Adult Food-security Survey module	The NCI's [b] 26-item validated Dietary Screener Questionnaire was used to assess the frequency of intake in the past month for selected foods/drinks	Estimated mean intake of food groups<br>FV [g], dairy, calcium, whole grains, fiber, added sugars, sugar from sugar-sweetened beverage	Yes<br>Estimated mean intake of food groups ± SD [h]<br>FV [g], men FS [f]: 2.3 ± 0.07, FI [e]: 1.8 ± 0.07, women FS [f]: 2.0 ± 0.04, FI [e]: 1.6 ± 0.06, added sugar, men FS [f]: 14.2 ± 0.5 FI [e]: 16.5 ± 0.8, women FS [f]: 10.7 ± 0.2, FI [e]: 12.4 ± 0.6, sugar, men FS [f]: 5.4 ± 4.6, FI [e]: 8.3 ± 6.4, women FS [f]: 3.6 ± 0.2, FI [e]: 5.5 ± 0.6	

Author, year, location (state)	Study design, participant characteristics	Food-security assessment method	Dietary assessment method	Dietary marker measured	Significant main findings: Is food-security associated to dietary intake?	Definition/Description
**Adults**						
Jia, 2021, NHANES [a] from the United States	Cross sectional study, n = 13,956, Female, Male, 41-48 y	18-item U.S. Household Food-security Survey Module (HFSSM)	Day-1 24-hour dietary recall	Estimated mean HEI [b]–2015<br>HEI [b]–2015 total score for quartile 4, quartile 3, quartile 2, and quartile 1	Yes<br>Estimated Mean HEI [b] –2015 total score quartiles, percentage (SE [c])<br>HEI [b] –2015: quartile 4, FS [d]: 66.0 (1.3) marginally FS [d]:12.7 (0.8) FI [e]: 21.3 (1.1), quartile 3, FS [d]: 58.1 (1.4) marginally FS [d] : 15.2 (1.1) FI [e]: 26.7 (1.2), quartile 2, FS [d]: 55.4 (1.5) marginally FS [d]: 15.2 (0.9) FI [e]: 29.5 (1.2), quartile 1, FS [d]: 50.3 (1.5) marginally FS [d]:16.7 (1.2), FI [e]: 33.0 (1.6)	[a] National Health and Nutrition Examination Survey (NHANES) is a continuous, cross-sectional series of surveys of nationally representative samples of the resident, civilian, non-institutionalized US population; [b] Healthy Eating Index (HEI) measures adherence to the Dietary Guidelines for Americans based on adequacy and moderation components, with higher scores indicating better diet ; [c] Standard Error (SE); [d] Food security (FS); [e] Food insecurity (FI); [f] Defined using the NOVA food classification system; [g] Adjusted for sociodemographic, food secure as the reference category; [h] The Short Healthy Eating Index (sHEI), previously validated in college-aged students to assess diet quality as well as intake of individual food items was used to assess diet quality and healthy eating index measures a diet's consistency with the US Dietary Guidelines for Americans where points are assigned based on compliance with recommended intakes
Leung, 2022, NHANES [a] from the United States	Cross sectional study, n = 9190, Female, Male, 38-40 y	18-item U.S. Household Food-security Survey Module (HFSSM)	Two 24-hour dietary recalls were averaged	Estimated percentage of energy intake from ultra-processed foods [e]	Yes<br>Multivariate [g] analysis of the association between FS [d] and percentage of energy intake from ultra-processed foods [f] , % (95% CI), $P<0.05$<br>High FS [d]: 52.6 (51.6, 53.7), marginal FS [d]: 53.6 (52.2, 55.0), low FS [d]: 53.7 (52.3, 55.1), very low FS [d]: 55.7 (54.1, 57.4)	
Leung, 2019, NHANES [a] from the United States	Cross sectional study, n = 4383, Female, Male, 40 y	18-item U.S. Household Food-security Survey Module (HFSSM)	Two 24-hour dietary recalls were averaged	Estimated mean HEI [b]–2015<br>Total HEI [b]–2015	Yes<br>Estimated mean HEI [b]–2015, Mean ± SE [c]<br>FS [d]: 54.6 ± 0.6, FI [e]: 52.4 ± 0.8<br>Multivariate [g] analysis of the association between FS [d] and HEI [b]–2015, β (95% CI), $P<0.05$<br>FI [e]: β= −2.22(−3.35, −1.08)	
Marshall, 2023, Howard University (District of Columbia) (n = 286) or the	Cross sectional study, n = 189, Female, Male, 22-31 y	10-item U.S. Household Food-security Survey Module (HFSSM) Adult Food-	sHEI [h] survey	Estimated mean sHEI [h]<br>Total fruit and vegetable, total fruit, total vegetable, dairy servings, sugars, added sugars	Yes<br>Estimated mean sHEI [h] $p<0.05$<br>Total fruit and vegetable servings, cup, n (%): 1.81, FS [c]: 22 (21.6) FI [d]: 30 (34.5), 2.22, FS [c]:	

Author, year, location (state)	Study design, participant characteristics	Food-security assessment method	Dietary assessment method	Dietary marker measured	Significant main findings: Is food-security associated to dietary intake?	Definition/Description
			**Adults**			
Marshall, 2023 University of Iowa (Iowa) (n = 326) *Continued*		security Survey module		from sugar-sweetened beverages	41 (40.2) FI [e]: 35 (40.2) 2.53, FS [d]: 21 (20.6) FI [e]: 17 (19.5), 3.21 FS [d]: 18 (17.6) FI [e]: 5 (5.8), total fruit servings, cup, n(%): 0.52 FS [d]: 22 (21.6) FI [e]: 30 (34.5) 0.78, FS [d]: 33 (32.4) FI [e]: 34 (39.1) 0.99, FS [d]: 29 (28.4) FI [e]: 18 (20.7) 1.52 FS [d]: 18 (17.6), FI [e]: 5 (5.7), total vegetables servings, cup, n (%): 1.34, FS [d]: 40 (39.2) FI [e]: 55 (63.2) 1.40, FS [d]: 33 (32.4) FI [e]: 19 (21.8) 1.68, FS [d]: 8 (7.8) FI [e]: 5 (5.8) 1.84 FS [d]: 21 (20.6) FI [e]: 8 (9.2), adding sugars, teaspoon, n(%): 13.26, FS [d]: 63 (61.8) FI [e]: 34 (39.5) 16.00, FS [d]: 29 (28.4) FI [e]: 28 (32.6) 26.87, FS [d]: 10 (9.8) FI [e]: 24 (27.9), added sugars from sugar sweetened beverages, teaspoon n(%): 4.13, FS [d]: 44 (43.1) FI [e]: 22 (25.6) 5.73, FS [d]: 19 (18.6) FI [e]: 12 (13.9) 6.79, FS [d]: 29 (28.4) FI [e]: 28 (32.6) 15.78, FS [d]: 10 (9.8) FI [e]: 24 (27.9) Multivariate [g] analysis of the association between FS [d] and sHEI [h], β ± SE [c] (95% CI), P<0.05 For students who move from FI [e] to FS [d] status: β=3.86 ± 1.53 (0.85 – 6.8)	

Author, year, location (state)	Study design, participant characteristics	Food-security assessment method	Dietary assessment method	Dietary marker measured	Significant main findings: Is food-security associated to dietary intake?	Definition/Description
			**Adults**			
Mei, 2021, (Michigan)	Cross sectional study, n = 1033, Female, Male, ~19 y (college students)	6-item Short Form U.S. Household Food-security Survey Module (HFSSM)	National Cancer Institute 26-item DSQ [a] and BEV-Q [b]	Estimated mean intake of food groups<br>Fruits, vegetables including legumes, whole grains, dairy, added sugars from sugar-sweetened beverages, added sugars total, calcium, fiber, total sugar-sweetened beverages, regular (sweetened) soda, fruit drinks, energy and sports drinks, sweetened teas, sweetened coffees, flavored milk, plain milk, 100% fruit juice, diet soda, plain tea/coffee, water	Yes<br>Multivariate [c] analysis of the association between FS [d] and mean intake of food groups, relative difference (95% CI), P<0.05<br>Fruits, cup: 0.91 (0.84, 0.99), vegetables, cup: 0.91 (0.87, 0.96), dairy, cup: 1.10 (1.04, 1.17), added sugars from sugar-sweetened beverages, teaspoon: 1.10 (1.02, 1.18), added sugar total, teaspoon: 1.06 (1.01, 1.12), calcium, mg: 1.04 (1.01, 1.08), fiber, gr: 0.96 (0.93, 0.99), total sugar-sweetened beverages, oz: 1.56 (1.18, 2.06), energy and sports drinks, oz: 2.85 (1.54, 5.28), sweetened teas, oz: 2.21 (1.37, 3.58)	[a] Dietary Screener Questionnaire (DSQ); daily intakes for each dietary component were estimated using publicly available SAS macros; [b] Beverage Intake Questionnaire-15 (BEV-Q); [c] Adjusted for sociodemographic, food secure as the reference category; [d] Food security (FS); [e] Behavioral Risk Factors Surveillance System. The question asks: "In the past 30 days, have you been concerned about having enough food for you or your family?" Response categories were yes or no. Those who answered yes were deemed food insecure; [f] The Food Habits Questionnaire (FHQ) asks participants 24 introductory questions regarding whether they ate particular foods in the past month; [g] Standard Error (SE); [h] Food Insecurity (FI); [i] National Health and Nutrition Examination Survey (NHANES) is a continuous, cross-sectional series of surveys of nationally representative samples of the resident, civilian, non-institutionalized US population; [j] Healthy Eating Index (HEI) measures adherence to the Dietary Guidelines for Americans based on adequacy and moderation components,
Mello, 2010, (Rhode Island)	Cross sectional study, n = 1874, Female, Male, 40 y	Behavioral Risk Factors Surveillance System [e]	Food Habits Questionnaire (FHQ [f]) and a food frequency questionnaire (FFQ) over the past month	Estimated mean intake of food groups<br>Fruit (no juice), fruit (w/ juice), vegetables, vegetables (including fries), fruit (no juice) and vegetables (no fries), fruit (w/juice) and vegetables (no fries), fruit (w/juice) & vegetables (w/ fries)	Yes<br>Multivariate [c] analysis of the association between FS [d] and dietary outcomes, Means ± SE [g], P<0.05<br>Fruit (w/ juice), FS [d]: 3.06 ± 0.08, FI [h]: 3.27 ± 0.08	
Nguyen, 2015, NHANES [i] from the United States	Cross sectional study, n = 8333, Female, Male, 46 y	18-item U.S. Household Food-security Survey Module (HFSSM)	One 24-hour dietary recall	Estimated mean HEI [j]–2010 and dietary markers<br>HEI [j]-2010 total score, added sugar, solid fat, empty calories, total calories	Yes<br>Estimated mean HEI [j]–2010 and dietary markers P<0.05<br>HEI [j]–2010 total score, FS [d]: 46.6, FI [h]: 43.7, added sugar, FS [d]: 18.7, FI [h]: 22.0, empty	

Author, year, location (state)	Study design, participant characteristics	Food-security assessment method	Dietary assessment method	Dietary marker measured	Significant main findings: Is food-security associated to dietary intake?	Definition/Description
**Adults**						
Nguyen, 2015, NHANES [i] from the United States *Continued*					calories, FS [d]: 731.5, FI [h]: 787.9<br>Multivariable [c] regression of the association between FS [d] and HEI [j]–2010, β ± SE [g], P<0.05<br>Marginal FS [d], HEI [j]–2010 total score: -2.27 ± 0.55, low FS [d], HEI [h]–2010 total score: -1.63 ± 0.53, added sugar: 2.35 ± 0.73, very low FS [d], HEI [h]–2010 total score: -2.59 ± 0.67, added sugar: 4.94 ± 0.94, empty calories: 102.48 ± 24.87	with higher scores indicating better diet quality
Oddo, 2025, (Rhode Island and Connecticut)	Cross sectional study, n =1234, Female, Male, 24-47 y	6-item Short Form U.S. Household Food-security Survey Module (HFSSM)	VioScreen food frequency questionnaire (FFQ), a web-based, self-administered, graphical FFQ, which captured intake over the previous three months across 20 food groups using up to six plate-based graphical portion size options	Estimated mean HEI [a]–2015<br>Total fruits, whole fruits, total vegetables, greens and beans, whole grains, dairy, total protein foods, seafood and plant proteins, fatty acids, refined grains, sodium, added sugars, saturated fats, total score	Yes<br>Multivariable [b] regression of the association between FS [c] and HEI [a]–2015, β (95% CI), P<0.05<br>Total fruit and vegetables (excluding legumes) among FI [d] participants β= -0.18 (-0.34, -0.03)	[a] Healthy Eating Index (HEI) measures adherence to the Dietary Guidelines for Americans based on adequacy and moderation components, with higher scores indicating better diet quality; [b] Adjusted for sociodemographic, food secure the reference category; [c] Food security (FS); [d] Food Insecurity (FI); [e] Alternative Healthy Eating Index (aHEI) was chosen over standard HEI scores because aHEI scores have been found to be better predictors of cardiovascular and other major chronic diseases; [f] Standard Deviation (SD);
Sharpe, 2016, Columbia (South Carolina)	Cross sectional study, n = 202, Female, 25-50 y	6-item Short Form U.S. Household Food-security Survey Module (HFSSM)	Three 24-hour dietary recalls on two weekdays and one weekend day within 15 days	Estimated mean intake of dietary markers<br>Total calories, aHEI [e], total fat, saturated fat, trans fat, protein, carbohydrate, added sugars, fiber, sodium, vegetables, fruit, fruits and vegetables, total grains, whole	Yes<br>Estimated mean intake of dietary markers, mean ± SD [f]<br>Carbohydrate, FS [c]: 47.7± 8.0 FI [d]: 50.1 ± 7.8, lean meat, FS [c]: 2.6 ± 2.1 FI [d]: 2.0 ± 1.7	

**Author, year, location (state)**	**Study design, participant characteristics**	**Food-security assessment method**	**Dietary assessment method**	**Dietary marker measured**	**Significant main findings: Is food-security associated to dietary intake?**	**Definition/Description**
			**Adults**			
Sharpe, 2016, Columbia (South Carolina) *Continued*				grains, whole grains, refined grains, dairy, low-fat dairy, meat, lean meat, beans, meat alternatives, sweetened beverages, alcoholic beverages		[c] Food security (FS); [d] Food Insecurity (FI); [e] Diet ID™ is a validated tool that evaluates dietary patterns using a patented image-based algorithm (Diet Quality Photo Navigation), in which participants select food images that best represent their usual intake through a series of "this-or-that" comparisons; [f] Healthy Eating Index (HEI) measures adherence to the Dietary Guidelines for Americans based on adequacy and moderation components, with higher scores indicating better diet quality; [g] Adjusted for sociodemographic, food secure the reference category; [h] Odds ratio (OR)
Sklar, 2025, (California)	Cross sectional study, n = 166, Female, Male, Non-Binary, ~19 y (college students)	10-item U.S. Household Food-security Survey Model (HFSSM) Adult Food-security Survey module	Participants completed the Diet ID™ dietary assessment [e] during the in-person visit	Estimated mean HEI [f]–2015<br>Total HEI [f]–2015 and adherence to Mediterranean diet score	Yes<br>Estimated Mean HEI [f]–2015, P<0.05<br>High FS [c]:73.18 ± 20.68, marginal FS [c]: 79.03±18.59, low FS [c]: 71.04 ± 21.32<br>HEI [f] score was lower among very low FS [c]: 60.07 ± 24.54	
Wilcox, 2020, (South Carolina)	Cross sectional study, n = 465, Female, Male, 52 y	18-item U.S. Household Food-security Survey Module (HFSSM)	One 24-hour dietary recall	Estimated percentage of the sample meeting the Dietary Guidelines for Americans<br>Fat (% kcals), saturated fat (% kcals), protein (% kcals), carbohydrates (% kcals), fiber, fruits, vegetables, fruits and vegetables, whole grains, dairy, sweetened beverages, sodium	Yes<br>Multivariable [g] regression of the association between FS [c] and dietary markers<br>OR [h] (95% CI), P<0.05<br>Carbohydrates (% kcals), FS [c] vs FI [d]: 0.68 (0.46-0.99), whole grains (% of total grains), FS [c] vs FI [d]: 0.50 (0.28-0.92)	

Author, year, location (state)	Study design, participant characteristics	Food-security assessment method	Dietary assessment method	Dietary marker measured	Significant main findings: Is food-security associated to dietary intake?	Definition/Description
			**Adults, Older Adults**			
Aljahdali, 2024, HRS [a] from the United States	Cross sectional study, n = 6,220, Female n = 3639, Male n = 2581, ≥50 y	6-item Short Form U.S. Household Food-security Survey Module (HFSSM)	A validated Food Frequency Questionnaire (FFQ) was used to quantify consumption over the past year The FFQ contained 163 food and beverage items	Estimated mean of dietary caloric contribution of ultra-processed foods [b]	Yes<br>Estimated mean of dietary caloric contribution of ultra-processed foods, % mean (95% CI), P<0.05<br>Males: FS [c]:53 (52.0, 53.3) very low FS [c]: 57 (54.7, 59.7), females: FS [c]: 50 (49.0, 50·6) very low FS [c]: 54 (51.5, 56.3)	[a] Health and Retirement Study (HRS) from a United States; [b] Defined using the NOVA food classification system; [c] Food security (FS); [d] Foodsmart, is a telehealth provider platform with a large network of registered dietitians across the United States that includes a digital nutrition platform, which provides personalized recipe recommendations and meal planning; [e] The question asked was "How often does the food you buy not last and you don't have money to get more?"." If participants answered "sometimes" or "often" they would be considered food insecure; if they answered "never" to the question, they would be considered food secure; [f] Standard Deviation (SD); [g] Food insecurity (FI); [h] Healthy Eating Index (HEI) measures adherence to the Dietary Guidelines for Americans based on adequacy and moderation components, with higher scores indicating better diet quality; [i] Standard Error (SE); [j] Estimated Average Requirement (EAR); [k] Adequate Intake (AI)
Bakre, 2022, Foodsmart [d] from the United States	Longitudinal study, n = 4595, Female, Male, <40 y, 40-59 y, ≥60 y	One of two questions in a shortened food-security screener validated [e] and that is valid when compared to the 18-item U.S. Household Food-security Survey Module (HFSSM)	A validated Food Frequency Questionnaire (FFQ) called Nutriquiz was used to access the participants diet quality	Estimated mean of the Nutri-Score<br>The total Nutriscore for overall diet quality and components: fruits, vegetables, protein ratio, fat ratio, carbohydrate ratio, hydration, sodium, and final Nutriscore	Yes<br>Estimated mean of the Nutri-Score (SD [f]), P<0.05<br>Vegetable: FI [g]: 3.3 (1.5) FS [c]: 3.5 (1.4), fruit: FI [g]: 2.9 (2.2), FS [c]: 3.2 (2.2), carbohydrate ratio: FI [g]: 7.3 (1.7), FS [c]: 7.7 (1.7), fat ratio: FI [g]: 2.6 (2.6), FS [c]: 3.1 (2.9), protein ratio: FI [g]: 6.8 (3.2) FS [c]: 7.1 (3.2), sodium: FI [g]: 6.6 (4.3), FS [c]: 7.2 (3.9), hydration: FI [g]: 6.3 (2.4), FS [c]: 6.6 (2.2), baseline Nutriscore: FI [g]: 31.9 (8.3), FS [c]: 34.3 (8.6)	
Champagne, 2007, Lower Mississippi Delta region, (Mississippi)	Cross sectional study, n = 1,470, Female, Male, 18-75+ y	18-item U.S. Household Food-security Survey Module (HFSSM)	Two 24-hour dietary recalls, and a series of trailer questions were collected to estimate the usual intake, water consumption.	Estimated mean HEI [h] 1999-2000<br>Dairy, fruit, vegetable, grain, meat, fat, saturated fat, cholesterol, sodium, variety, and no sodium. Energy density was calculated by dividing total energy by total weight of the food consumed in grams, excluding water.	Yes<br>Estimated mean scores of HEI [h] 1999-2000, ± SE [i], P<0.05<br>HEI [h] -Vegetable: FS [c]: 5.64 ± 0.11, FI [g]: 4.36 ± 0.19<br>Estimated prevalence of usual intakes <EAR [j] or >the AI [k]<br>Met EAR [j] vitamin A: FS [c]: 33.37 ± 1.27, FI [g]: 20.10 ± 2.47, Met EAR [j] selenium: FS [c]: 88.23 ± 0.91, FI [g]: 80.56 ± 2.07	

Author, year, location (state)	Study design, participant characteristics	Food-security assessment method	Dietary assessment method	Dietary marker measured	Significant main findings: Is food-security associated to dietary intake?	Definition/Description
			**Adults, Older Adults**			
Champagne, 2007, Lower Mississippi Delta region, (Mississippi) *Continued*				Estimated prevalence of usual intakes <EAR [j] or >the AI [k] Vitamins A, E, B6, B12, C, thiamin, riboflavin, niacin, folate, calcium, magnesium, phosphorus, iron, copper, selenium, zinc, carbohydrates, protein, linoleic acid, fiber and energy density		
Choi, 2022, HRS [a] from the United States	Cross sectional study, n = 6,203, Female, Male, 53-100 y	6-item Short Form U.S. Household Food-security Survey Module (HFSSM)	Food Frequency Questionnaire (FFQ) over the past 12 months	Estimated mean HEI [b]–2015 Total HEI [b]–2015	Yes Multivariable [c] analysis of the association between FS [d] and HEI [b]–2015 (95% CI), P<0.05 FI [e] Coefficient: -1.18 (- 2.29, - 0.06)	[a] Health and Retirement Study (HRS) from the United States; [b] Healthy Eating Index (HEI) measures adherence to the Dietary Guidelines for Americans based on adequacy and moderation components, with higher scores indicating better diet quality; [c] Adjusted for sociodemographic; food secure the reference category; [d] Food security (FS); [e] Food-insecurity (FI); [f] National Health and Nutrition Examination Survey (NHANES) is a continuous, cross-sectional series of surveys of nationally representative samples of the resident, civilian, non-institutionalized US population; [g] National Cancer Institute (NCI) method; [h] Estimated Average Requirement (EAR); [i] Adequate Intake; [j] Standard Error
Cowan, 2019, NHANES [f] from the United States	Cross sectional study, n = 9954, Female, Male, > 19 y	18-item U.S. Household Food-security Survey Module (HFSSM)	Two 24-hour dietary recalls and the NCI [g] method was used to estimate nutrient usual intake	Estimated prevalence of usual intakes <EAR [h] or > the AI [i] Calcium, iron, magnesium, potassium, zinc, choline, folate, vitamin B6, vitamin C, vitamin D	Yes Estimated prevalence of usual intakes <EAR [h] or > the AI [i], ± SE, P<0.0125 Magnesium, men FI [e]: 57.2 ± 1.7 FS [d]: 43.9±1.5, women FI [e]: 56.9±2.6 FS [d]: 40.9±1.4, potassium, men FI [e]: 25.0±2.5 FS [d]: 37.0±1.6, women FI [e]: 24.0±3.1 FS [d]: 35.0±1.7, zinc, men FI [e]: 20.1±2.4 FS [d]: 11.3±1.1, vitamin B6, men FI [e]: 6.1±1.3 FS [d]: 1.4±0.4, women FI [e]: 19.0±1.6 FS [d]: 8.8±0.9, vitamin C, men FI [e]: 49.0±3.8 FI [e]: 37.0±1.7, women FI [e]: 42.0±2.4 FS [d]: 29.01.3, vitamin D: men FI [e]: 79.2±1.5 FS [d]: 64.1±1.2, women FI [e]: 74.7±1.7 FS [d]: 56.2±1.2	

Author, year, location (state)	Study design, participant characteristics	Food-security assessment method	Dietary assessment method	Dietary marker measured	Significant main findings: Is food-security associated to dietary intake?	Definition/Description
			**Adults, Older Adults**			
Gupta, 2020, Two communities in Cleveland and Columbus (Ohio)	Cross sectional study, n = 450, Female, Male, 38-65 y	6-item Short Form U.S. Household Food-security Survey Module (HFSSM)	Three 24-h dietary recalls (two on weekdays and one on the weekend) were averaged	Estimated mean intake of dietary markers<br>HEI [a]–2010, daily fruit and vegetable intake and daily energetic intake	No	[a] Healthy Eating Index (HEI) measures adherence to the Dietary Guidelines for Americans based on adequacy and moderation components, with higher scores indicating better diet quality; [b] Alternative
Koziatek, 2025, Sullivan County (New York)	Cross sectional study, n=1,433, Female, Male, 18-80 y	Questions on food-insecurity and housing insecurity based on validated brief screening tools	Food frequency questionnaire (FFQ). The study did not report the dietary period assessed (e.g., the last seven days, month)	Estimated mean aHEI [b]<br>Vegetables, fruits, whole grains, sugary beverages, nuts and legumes, red meats, trans- fats, fish fatty acids, polyunsaturated fats, sodium intake and alcohol	Yes<br>Estimated mean percentage of FI [c] across tertiles of aHEI [b], P<0.05<br>FI [c]: lowest aHEI [b] tertile: 39.8%, middle aHEI [b] tertile: 27.1%, highest aHEI [b] tertile: 17.7%	Healthy Eating Index (aHEI) was chosen because it has predictors of cardiovascular and other major chronic diseases; [c] Food-insecurity (FI); [d] Health and Retirement Study (HRS) from the United States;
Lee, 2025, HRS [d] from the United States	Cross sectional and longitudinal study, n=6,783 and n=4,923, respectively, Female, Male, ≥ 50 y at initial food-insecurity assessment	6-item Short Form U.S. Household Food-security Survey Module (HFSSM). Long-term food-insecurity was estimated by averaging 2 items from the 18- item U.S. Household Food-security Survey Module (HFSSM)	Dietary intake was assessed using a validated 164-item semi-structured Food frequency questionnaire (FFQ) from the 2013 Health Care and Nutrition Study (HCNS)	Five measures of diet quality: the plant-based diet index (PDI) [e], healthful PDI [e], unhealthful PDI (uPDI) [e], MedDiet score [f], and MIND diet index [g]	Yes<br>Multivariate [h] analysis of the association between FS [i] and diet quality indices, β (95% CI), P<0.05<br>6-item HFSSM:<br>uPDI [b]: low FS [i]: β=0.51 (-0.22, 1.24), very low FS [i]: β=1.46 (0.45, 2.48), MIND [g] diet: low FS [i]: β=-0.15 (-0.37, 0.06), very low FS [i]: β= -0.24 (-0.49, 0.003)<br>Long-term FS [I]<br>Very low FS [i] was associated with lower scores on overall PDI [e]: β= -1.15 (-2.37, 0.08), MedDiet score [f]:β=− 1.33 (-2.24, -0.42), and MIND [g] diet index: β= -0.58 (-1.00, -0.17), and higher scores on the uPDI [e]: β= 1.92 (0.15,3.69)	[e] Plant-based diet index (PDI), healthful PDI (hPDI), unhealthful PDI (uPDI); [f] The Mediterranean diet score (MedDiet) included: no refined grains, vegetables, potatoes, fruit, dairy, red and processed meat, fish, poultry, legumes, olive oil, and alcohol; [g] The MedDiet Approaches to Stop Hypertension Intervention for Neurodegeneration Delay (MIND) diet score was derived from brain-healthy food groups (e.g. whole grains) and brain-unhealthy food groups (e.g. processed meats); [h] Adjusted for sociodemographic;food secure the reference category; [i] Food security (FS)

Author, year, location (state)	Study design, participant characteristics	Food-security assessment method	Dietary assessment method	Dietary marker measured	Significant main findings: Is food-security associated to dietary intake?	Definition/Description
**Adults, Older Adults**						
Lewis, 2024, Baltimore (Maryland)	Cross sectional study, n = 127, Female, Male, 21–75 y	6-item Short Form U.S. Household Food-security Survey Module (HFSSM)	Dietary quality indicators. Beverage intake. Beverage intake was measured using the BEVQ-15 [a]	Estimated mean intake of dietary markers Beverage, fruit and vegetables, and fiber	Yes Estimated mean intake of dietary markers, SD [b], P<0.05 High/Marginal FS [c]: fruits and vegetables: 4.19 ± 2.18, total fiber: 17.16 ± 6.98, low FS [c]: fruits and vegetables: 3.15 ± 2.09, fiber: 12.75 ± 7.18	[a] Beverage Intake Questionnaire (BEVQ-15) asks respondents to indicate approximately 1) how often and 2) how much each time, they drank fifteen researcher-selected beverages, including sugar-sweetened beverages, in the past month. Fruit and vegetable intake. Fruit and vegetable intake was measured using the Block Fruit/Vegetable/Fiber (FVF) Screener. The screener includes 7 questions about fruit and vegetable intake and 3 questions about foods high in fiber. Fiber intake from fruits and vegetables, beans, and whole grains was also measured using the Block FVF Screener, which, in addition to producing a score associated with fruit and vegetable intake, produces point estimates for various daily nutrient intakes including vitamin C, magnesium, potassium, and dietary fiber; [b] Standard deviation (SD); [c] Food-security (FS); [d] National Health and Nutrition Examination Survey (NHANES) is a continuous, cross-sectional series of surveys of nationally
Liu, 2024, NHANES [d] from the United States	Cross sectional study, n = 51,703, Female, Male, 20–44 y, 45–64 y, ≥65 y	10-item U.S. Household Food-security Survey Module (HFSSM) Adult Food-security Survey module	One or up to two 24-hour dietary recalls were averaged	Estimated means of diet quality scores AHA[e] 2020 diet score, overall diet quality and the HEI [f]–2015	Yes Estimated means of diet quality scores by NHANES [d] survey cycle, from 1999 to 2020, 95% CI, P<0.05 Cycle 1999-2000: FI [g]: Participants, n (%): 497 (8.27), Poor Diet Quality (%): 51.3 (39.7–62.8), AHA[e] Score: 31.0 (27.7–34.2), HEI [f]-2015: 47.1 (44.5–49.6), Marginal FS [c]: n (%): 316 (4.84), Poor Diet Quality: 58.2 (49.1–66.7), AHA[e] score: 30.7 (29.0–32.7), HEI [f]-2015: 47.1 (44.8–49.3), Full FS [c]: n (%): 3331 (85.1), Poor Diet Quality: 47.9 (42.9–52.9), AHA [b] score: 33.1 (31.6–34.7), HEI [f]-2015: 49.4 (47.6–51.1) Cycle 2017-2020: FI [g]: n (%): 1647 (16.1), Poor Diet Quality: 48.2 (42.3–54.1), AHA[e] score: 33.2 (31.7–34.8), HEI [f]-2015: 48.9 (47.2–50.5), marginal FS [c]: n (%): 1067 (10.3), Poor Diet Quality: 50.9 (44.4–57.4),	

Author, year, location (state)	Study design, participant characteristics	Food-security assessment method	Dietary assessment method	Dietary marker measured	Significant main findings: Is food-security associated to dietary intake?	Definition/Description
			**Adults, Older Adults**			
Liu, 2024, NHANES [d] from the United States *Continued*					AHA[e] score: 32.8 (31.2–34.5), HEI [f]-2015: 49.2 (47.6–50.8), Full FS [c]: n (%): 4555 (68.7), Poor Diet Quality: 33.0 (30.2–35.9), AHA[e] score: 38.0 (37.2–38.9), HEI [f]-2015: 53.8 (52.8–54.7)	representative samples of the resident, civilian, non-institutionalized US population; [e] American Heart Association (AHA) score reflects higher intake of fruits and vegetables, whole grains, fish, and plant proteins and lower intake of sugar-sweetened beverages, processed meats, saturated fat, and sodium; [f] Healthy Eating Index (HEI) measures adherence to the Dietary Guidelines for Americans based on adequacy and moderation components, with higher scores indicating better diet quality; [g] Food insecurity (FI)
Liu, 2019, Central Indiana	Cross sectional study, n = 270, Female, Male, 21-80 y	18-item U.S. Household Food-security Survey Module (HFSSM)	One 24-hour recall was recorded using the ASA24 [a] version 2014	Estimated mean HEI [b] -2010<br>Total HEI [b] –2010	No	[a] The Automated Self-Administered 24-hour Dietary Recall (ASA24™–2014), an internet-based 24-hour dietary recall, with optional staff assistance; [b] Healthy Eating Index (HEI) measures adherence to the Dietary Guidelines for Americans based on adequacy and moderation components, with higher scores indicating better diet quality; [c] National Health and
Ma, 2021, NHANES [c] from the United States	Cross sectional study, n = 30,251, Female, Male, 18-80+ y	18-item U.S. Household Food-security Survey Module (HFSSM)	Two 24-hour dietary recalls were averaged	Estimated prevalence of macronutrient and micronutrient intakes aligned with the RDAs [d]<br>Protein, carbohydrates, vitamin A, fiber, thiamine B1, riboflavin B2, niacin B3, vitamin B6, folate B9,	Yes<br>Estimated prevalence of macronutrient and micronutrient intakes aligned with the RDAs [d],<br>% (95% CI), $P<0.05$<br>FSW [e]: protein: 83.8 (82.8–84.8), carbohydrates: 88.4	

Author, year, location (state)	Study design, participant characteristics	Food-security assessment method	Dietary assessment method	Dietary marker measured	Significant main findings: Is food-security associated to dietary intake?	Definition/Description
**Adults, Older Adults**						
Ma, 2021, NHANES [c] from the United States *Continued*				vitamin B12, vitamin C, vitamin D, vitamin K, phosphorus, magnesium, calcium, iron, and zinc	(87.5–89.2), vitamin A: 31.5 (29.9–33.2), fiber: 16.1 (14.8–17.4), thiamine B1: 66.7 (65.0–68.3), riboflavin, B2: 85.7 (84.5–86.8), niacin B3: 82.1 (81.0–83.2), vitamin B6: 64.0 (62.4–65.5), folate B9: 32.8 (31.0–34.6), vitamin B12: 74.9 (73.6–76.2), vitamin C: 42.9 (41.2–44.5), vitamin K: 42.2 (40.7–43.8), phosphorus: 89.4 (88.4–90.4), magnesium: 27.6 (26.2–29.1), iron: 46.7 (45.1–48.3) zinc: 61.6 (60.0–63.1), FIW [f]: protein: 78.0 (75.3–80.6), carbohydrates: 85.9 (83.6–87.9), vitamin A: 21.3 (19.0–23.7), fiber: 10.2 (8.5–12.2), thiamine B1: 61.1 (58.3–63.9), riboflavin B2: 77.6 (75.3–79.7), niacin B3: 73.6 (71.1–76.0), vitamin B6: 57.4 (54.8–59.9), folate B9: 26.8 (24.4–29.2), vitamin B12: 69.3 (66.5–72.0), vitamin C: 34.7 (32.2–37.3), vitamin K: 28.7 (26.0–31.5), phosphorus: 84.1 (82.3–85.7), magnesium: 18.8 (16.8–20.9), iron: 33.7 (30.9–36.6), zinc: 53.5 (50.7–56.3) FSM [g]: thiamine B1: 82.0 (80.8–83.1), riboflavin B2: 89.6 (88.4–90.7), phosphorus: 97.0 (96.5–97.5), FIM [h]: thiamine B1: 73.4 (70.4–76.2), riboflavin B2: 77.4 (74.7–79.8), phosphorus: 93.4 (91.5–94.9)	Nutrition Examination Survey (NHANES) is a continuous, cross-sectional series of surveys of nationally representative samples of the resident, civilian, non-institutionalized US population; [d] Recommended Dietary Allowances (RDA); [e] Food secure women (FSW); [f] Food insecure women (FIW); [g] Food secure men (FSM); [h] Food insecure men (FIM)

Author, year, location (state)	Study design, participant characteristics	Food-security assessment method	Dietary assessment method	Dietary marker measured	Significant main findings: Is food-security associated to dietary intake?	Definition/Description
**Adults, Older Adults**						
Marmash, 2021, Windham County (Connecticut)	Cross sectional study, n=83, Female, Male, ≥19 y; 19-30, 31-44, 45-64, ≥65 y	18-item U.S. Household Food-security Survey Module (HFSSM)	The NCI DSQ [a] was used to estimate the daily intake of food groups of the 83 participants. This 26-item questionnaire includes questions about the frequency of food consumption over the past 30 days	Estimated mean intake of food groups<br>Whole grain, added sugars, dairy, fruits and vegetables including legumes and French fries, fruits and vegetables including legumes and excluding French fries, vegetables including legumes and excluding French fries, fruits, sugar-sweetened beverages	No	[a] NCI DSQ: National Cancer Institute's Dietary Screener Questionnaire, a research tool used to estimate a person's dietary intake; [b] National Health and Nutrition Examination Survey (NHANES) is a continuous, cross-sectional series of surveys of nationally representative samples of the resident, civilian, non-institutionalized US population; [c] Healthy Eating Index (HEI) measures adherence to the Dietary Guidelines for Americans based on adequacy and moderation components, with higher scores indicating better diet quality; [d] Food security (FS); [e] Food-insecurity (FI); [f] The Automated Self-Administered 24-hour Dietary Recall (ASA24™), an internet-based 24-hour dietary recall, with optional staff assistance; [g] [b] National Cancer Institute (NCI) method; [h] Adjusted for sociodemographic; food secure the reference category; [i] Standard Error (SE)
Rehm, 2011, NHANES [b] from the United States	Cross sectional study, n = 4744, Female, Male, ≥ 20 y; 20–29, 30–44, 45–64, 65–74, 75 y	18-item U.S. Household Food-security Survey Module (HFSSM)	One 24-hour dietary recall	Estimated mean HEI [c]–2005<br>Total HEI [c]-2005	Yes<br>Estimated mean HEI [c]–2005 (95% CI), $P<0.05$<br>Full FS [d]: 59.7 (57.7, 61.7)<br>Any FI [e]: 54.3 (51.2, 57.3)	
Rivera, 2020, (Indiana)	Cross sectional study, n = 194, Female, 18-64 y	18-item U.S. Household Food-security Survey Module (HFSSM)	Two ASA24 [f] were averaged using the NCI [g] method to estimate nutrient usual intake	Estimated mean HEI [c]–2010<br>Total vegetables, greens and beans, total fruit, whole fruit, total protein foods, seafood and plant proteins, refined grains, whole grains, dairy, fatty acid, sodium, empty calories and total score	Yes<br>Multivariable [h] regression of the association between FS [d] and HEI [c]–2010, mean ± SE [i], $P\leq 0.05$<br>Total HEI [a]–2010, FS [d]: 46.1 ± 1.7 FI [e]: 41.3 ± 1.1, whole grains, FS [d]: 2.9 ± 0.4 FI [e]: 1.8 ± 0.3	
Robaina, 2013, Hartford (Connecticut)	Cross sectional study, n = 212, Female, Male, 18-60+ y	18-item U.S. Household Food-security Survey Module (HFSSM)	Block Food Frequency Screener. Participants were asked 10 questions about their usual intake of fruit, vegetables, and fiber. Possible scores ranged from 0-50	Estimated mean percentage of intake of dietary markers<br>Score for fruit, vegetables, and fiber	Yes<br>Estimated mean percentage of intake of dietary markers, $P<0.05$<br>Fruit, vegetables, fiber scores<br>Low scores: FS [d]: 29.4, FI [e]: 52.2	

Author, year, location (state)	Study design, participant characteristics	Food-security assessment method	Dietary assessment method	Dietary marker measured	Significant main findings: Is food-security associated to dietary intake?	Definition/Description
**Adults, Older Adults**						
Wright, 2020, 24 rural, high poverty counties (Indiana, Michigan, Missouri, Nebraska, Ohio, and South Dakota)	Cross sectional study, n = 579, Female, Male, 18–44, 45–64, ≥65 y	18-item U.S. Household Food-security Survey Module (HFSSM)	Up to three ASA24 [a] -2014 recalls were averaged using the NCI [b] method	Estimated mean HEI [c]–2010<br>Total vegetables, greens and beans, whole grains, dairy, total protein food, seafood and plant proteins, fatty acids, sodium, refined grains, empty calories, total score<br>Estimated mean usual nutrient intake and usual intake of food groups for risk level<br>Energy, potassium, choline, magnesium, calcium, vitamin A, vitamin D, vitamin E, vitamin C, and iron. The variables for usual intake of food groups of “high risk” and “low risk” include total fruits, total vegetables, daily green vegetables, whole grains, dairy.	Yes<br>Estimated mean HEI [c]–2010, mean ± SD [d], P<0.0167<br>Whole grains: FS [e]: 2.8 ± 0.4 Low FS [e]: 1.8 ± 0.3, 95% CI for mean difference: FS [e] vs low FS [e]: 0.11, 1.85<br>Estimated mean usual nutrient intake and usual intake of food groups for “high risk” and “low risk”, Ratios, (95% CI), P<0.0167<br>Dark green vegetables: Ratio of mean usual intake FS [e] /low FS [e] by risk level High risk level: 1.60 (0.24, 0.95), low risk level: 1.61 (0.27, 0.95), ratio of mean usual intake FS [e] /very low FS [e] by risk level, high risk level: 1.58 (0.22, 0.94)<br>Low risk level: 1.59 (0.23, 0.95), Dairy: Ratio of mean usual intake FS [a]/very low FS [e] by risk level, high risk level: 1.30 (0.10, 0.50), low risk level: 1.24 (0.07, 0.41)<br>Iron, mg/d: FS [e]: 10.5 ± 0.6, low FS [e]: 11.8 ± 0.7, very low FS [e]: 9.9 ± 0.6, 95% CI for mean difference: FS [e] vs. very low FS [e]: 0.93 (-0.55, -0.11)	[a] The Automated Self-Administered 24-hour Dietary Recall (ASA24™–2014), an internet-based 24-hour dietary recall, with optional staff assistance; [b] National Cancer Institute (NCI) method; [c] Healthy Eating Index (HEI) measures adherence to the Dietary Guidelines for Americans based on adequacy and moderation components, with higher scores indicating better diet quality; [d] Standard Deviation (SD); [e] Food security (FS)

Author, year, location (state)	Study design, participant characteristics	Food-security assessment method	Dietary assessment method	Dietary marker measured	Significant main findings: Is food-security associated to dietary intake?	Definition/Description
			**Older Adults**			
Fulay, 2024, Pittsburgh and Memphis (Pennsylvania, Tennessee)	Longitudinal cohort study, n = 2468, Female, Male, 72-78 y	Food insufficiency (a screener for food-insecurity): Individuals who indicated that “there is enough of the kinds of food we want to eat” were classified as food insufficient.	Food Frequency Questionnaire (FFQ) and HEI[a] -1995 were used to estimate the dietary quality.	Estimated mean HEI [a] – 1995 score<br>Total HEI[a]-1995	Yes<br>Multivariate [b] analysis of the association between FI [c] and HEI [a]–1995, β (95% CI), $P<0.05$<br>FI [c]: β= -1.5(-2.84, -0.12)	[a] Healthy Eating Index (HEI) measures adherence to the Dietary Guidelines for Americans based on adequacy and moderation components, with higher scores indicating better diet quality; [b] Adjusted for sociodemographic; food sufficiency was used as the reference category; [c] Food insufficiency (FI); [d] National Health and Nutrition Examination Survey (NHANES); [e] Third National Health and Nutrition Examination Survey (NHANES III); [f] Nutrition Survey of the Elderly in New York State (NSENY); [g] Longitudinal Study of Aging (LSOA); [h] Nutritional Screening Initiative Checklist (NSIC) included the question “Do you have enough money to buy the food you need most of the time?”); [i] Food insecure and program participant (FIP); [j] Food insecure and program nonparticipant (FINP); [k] Food secure and program participant (FSP); [l] Food secure and program nonparticipant (FSNP)
Lee, 2001, from the NHANES [d], NHANES III [e], NSENY [f], LSOA[g], (New York)	Longitudinal cohort study, NHANES III [e], n = 6596, NSENY [f] n = 553, LSOA[g], n = 7527, Female, Male, NHANES [d], 60-90 y, NSENY [f], 60-90 y, 60-96 y	In the NHANES [e] III, the family FI [c] question was used to determine food-insecurity status. In the NSENY [a] survey, three items were used to measure food-insecurity status during the past 6 month. In the LSOA, a direct question asking food-insecurity status was not available. They chose the question “Do you have difficulty in preparing your own meals?” to indicate need for food assistance among older adults	One 24-hour dietary recall was used to estimate the nutrient intake. The NSENY [f] included a nutritional risk scale adopted from the 10-item NSIC [h].	Estimated mean nutritional risk<br>Nutritional Risk score	Yes<br>Estimated mean nutritional risk, $P<0.05$<br>Nutritional Risk Score<br>FIP [i]: 5.17, FINP [j]: 4.15<br>FSP [k]: 2.99, FSNP [l]: 2.90	

Author, year, location (state)	Study design, participant characteristics	Food-security assessment method	Dietary assessment method	Dietary marker measured	Significant main findings: Is food-security associated to dietary intake?	Definition/Description
**Older Adults**						
Lee, 2001, from the NHANES III [a], NSENY [b], (New York)	Cross sectional study, n = 6558, Female, Male, 60-90 y	In the NHANES III [a], the family food insufficiency question was used to determine food-insecurity status. In the NSENY [b] survey, three items were used to measure food-insecurity status during the past 6 month.	From NHANES III [a], one 24-hour dietary recall was used to estimate the nutrient intake	Estimated mean nutrient intakes<br>Energy, protein, total fat, saturated fat, carbohydrate, cholesterol, vitamin A, vitamin E, vitamin C, niacin, vitamin B6, thiamin, riboflavin, vitamin B12, folate, calcium, phosphate, magnesium, iron, zinc, sodium	Yes<br>Estimated mean nutrient intakes, P<0.05<br>Energy: FI [c]: 5978.7, FS [d]: 6620.9, protein: FI [c]: 54.3, FS [d]: 63, saturated fat: FI [c]: 15.6, FS [d]: 17.7, carbohydrate: FI [c]: 178, FS [d]: 197.6, niacin: FI [c]: 15.3, FS [d]: 18.2, vitamin B6: FI [c]: 1.3, FS [d]: 1.5, vitamin B12: FI [c]: 2.6 FS [d]: 3.1, magnesium: FI [c]: 220.9 FS [d]: 241, iron: FI [c]: 10.6, FS [d]: 12.5, zinc: FI [c]: 7.5, FS [d]: 8.8	[a] Third National Health and Nutrition Examination Survey (NHANES III); [b] Nutrition Survey of the Elderly in New York State (NSENY); [c] Food insufficient (FI); [d] Food sufficient (FS); [e] National Health and Nutrition Examination Survey (NHANES) is a continuous, cross-sectional series of surveys of nationally representative samples of the resident, civilian, non-institutionalized US population; [f] Adjusted for sociodemographic; food secure the reference category; [g] Healthy Eating Index (HEI) measures adherence to the Dietary Guidelines for Americans based on adequacy and moderation components, with higher scores indicating better diet quality; [h] Alternate HEI-2010 (aHEI-2010) is a measure of diet quality predictive of major chronic disease risk; [i] The Mediterranean diet (MedDiet) score is a measure of adherence to the traditional MedDiet. Scored out of 55 points, higher scores are awarded to high
Leung, 2021, NHANES [e] from the United States	Cross sectional study, n = 5097, Female, Male, ≥60 y	18-item U.S. Household Food-security Survey Module (HFSSM)	Two 24-hour dietary recalls were averaged	Estimated mean diet quality indices<br>HEI [g] -2015, aHEI [h] -2010 and MedDiet score[i]	Yes<br>Multivariate [f] analysis of the association between FS [j] and diet quality indices, β (95% CI), P<0.05<br>HEI [g]-2015: marginally FS [j]: β= -1.43 (-3.18, 0.33), FI [k]: β= -1.90 (-3.70, -0.09)<br>aHEI-2010: marginally FS [j]: β= -2.42 (-3.76, -1.07), FI [k]: β= -1.47 (-2.51, -0.44)<br>MedDiet score [i]: marginally FS [j]: β= -0.62 (-1.11, -0.12), FI [k]: β=-0.54 (-1.06, -1.01)	

Author, year, location (state)	Study design, participant characteristics	Food-security assessment method	Dietary assessment method	Dietary marker measured	Significant main findings: Is food-security associated to dietary intake?	Definition/Description
**Older adults**						
Leung, 2021, NHANES [e] from the United States *Continued*					Quintiles of diet quality indices (comparing top quintile to bottom four quintiles), OR[l] (95% CI), P<0.05<br>aHEI [h] -2010: marginally FS [j]: OR[l] =0.58 (0.41, 0.83), FI [k]: OR[l] =0.58 (0.43, 0.77)	intakes of whole grains, fruits, vegetables, potatoes, legumes, fish, and monounsaturated fats; low intakes of red meat, poultry, and full-fat dairy products; and moderate alcohol consumption; [j] Food insecure (FI); [k] Food secure (FS); [l] Odds ratio (OR)
Vaudin, 2022, NHANES [a] from the United States	Cross sectional study, n = 4820, Female, Male, ≥60 y	18-item U.S. Household Food-security Survey Module HFSSM Physical food-security was measured [b]. Four separate categories: 1) those who were both economically and physically food insecure, 2) those who were physically food insecure only, 3) those who were economically food insecure only, and 4) those who were completely food secure based on these 2 dimensions.	Two 24-hour dietary recalls were averaged	Estimated mean HEI [c] -2015<br>Total fruit, whole fruit, total vegetables, greens and beans, whole grains, dairy, total protein foods, seafood and plant proteins, fatty acids, refined grains, sodium, added sugars, saturated fats, and total score	Yes<br>Mean HEI [c]-2015 scores (SE [d]) by economic and physical food-security status (food secure is the reference group), P<0.05<br>Total HEI [c]-2015 score: Phys [e] and. Econ [f] FI [g]: 51.7 (0.67), Phys [e] FI [g] only: 54.5 (0.55), Econ [f] FI [g] only: 53.1 (1.08); total vegetables: Phys [e] and Econ [f] FI [g]: 3.0 (0.11), Phys [e] FI [g] only: 3.3 (0.09); green and beans: Phys [e] and Econ [f] FI [g]: 1.8 (0.15), Phys [e] FI [g] only: 1.8 (0.10); whole fruit: Phys [e] and Econ [f] FI [g]: 2.4 (0.17); whole grains: Phys [e] and Econ [f] FI [g]: 2.8 (0.16); dairy: Econ [f] FI [g] only: 4.5 (0.25); total protein foods: Phys [e] FI [g] only: 4.4 (0.04); seafood and plant proteins: Phys [e] and Econ [f] FI [g]: 2.4 (0.14), Phys [e] FI [g] only: 2.9 (0.10), Econ [f] FI [g] only: 2.5 (0.18); refined grains: Econ	[a] National Health and Nutrition Examination Survey (NHANES) is a continuous, cross-sectional series of surveys of nationally representative samples of the resident, civilian, non-institutionalized US population; [b] The ability to shop was assessed with the question "By yourself and without using any special equipment, how much difficulty do you have going out to things like shopping, movies, or sporting events?" and the question "By yourself and without using any special equipment, how much difficulty do you have lifting or carrying something as heavy as 10 pounds?" Ability to cook was assessed with the question "By yourself and without using any special

**Author, year, location (state)**	**Study design, participant characteristics**	**Food-security assessment method**	**Dietary assessment method**	**Dietary marker measured**	**Significant main findings: Is food-security associated to dietary intake?**	**Definition/Description**
			**Older adults**			
Vaudin, 2022, NHANES [a] from the United States *Continued*					[f] FI [g] only: 6.0 (0.30); added sugars: Phys [e] and Econ [f] FI [g]: 6.7 (0.26), Phys [e] FI [g] only: 7.0 (0.11) Multivariate [h] analysis of the association between FS and HEI [c]-2015, β (95% CI), P<0.05 Phys [e] FI [g] only: β= -2.83 (-4.18, -1.47), Econ [f] FI [g] only: β= -3.64 (-5.85, -1.42), Phys [e] and Econ [f] FI [g]: β= -4.65 (-5.93, -3.38)	equipment, how much difficulty do you have preparing your own meals?" Those who had at least some difficulty with ≥1 of these activities were considered physically food insecure, and everyone else was considered physically food secure; [c] Healthy Eating Index (HEI) measures adherence to the Dietary Guidelines for Americans based on adequacy and mod-
Wang, 2025, NHANES [a] from the United States	Cross sectional study, n = 2592, Female, Male, ≥60 y	18-item U.S. Household Food-security Survey Module (HFSSM)	Two 24-hour dietary recalls were averaged	Estimated mean HEI [c] -2020 Whole fruits, total fruits, greens and beans, total vegetables, dairy, whole grains, total protein foods, seafood and plant proteins, refined grains, fatty acids, saturated fats, added sugars, sodium, and total score Estimated mean MedDiet score [i] Adherence to the MedDiet[i] was assessed based on intakes of alcohol, red and processed meat, seafood, whole grains, legumes, nuts, fruits, vegetables (excluding potatoes), and the monounsaturated-to-saturated fatty acid ratio	Yes Multivariate [h] analysis of the association between FS and adherence to the and HEI [c]-2020 and MedDiet [i] scores indices, OR [j] (95% CI), P<0.05 MedDiet[i]: OR= 0.48 (0.31-0.67), HEI-2020 [d]: OR=0.61 (0.37, 0.84)	eration components, with higher scores indicating better diet quality; [d] Standard Error (SE); [e] Physical (Phys); [f] Economic (Econ); [g] Food insecure (FI); [h] Adjusted for sociodemographic; food secure the reference category; [i] Mediterranean Diet (MedDiet); participants received one point for each component if their intake exceeded the study population median, except for alcohol and red/processed meat. Component scores were summed to yield a maximum Mediterranean diet score of 9, with higher scores indicating greater adherence; [j] Odds ratios (OR)

Author, year, location (state)	Study design, participant characteristics	Food-security assessment method	Dietary assessment method	Dietary marker measured	Significant main findings: Is food-security associated to dietary intake?	Definition/Description
			**Pregnant Women**			
Gamba, 2016, NHANES [a] from the United States	Cross sectional study, n = 688, Female, Pregnant women, ≤20- >30 y	18-item U.S. Household Food-security Survey Module (HFSSM)	One to two 24-hour dietary recalls were averaged	Estimated mean aHEI–P [b] Vegetables, fruit, ratio of white to red meat, fiber, polyunsaturated to saturated fat ratio, calcium, folate, iron, total score	Yes Multivariate [c] analysis of FS [d] and aHEI–P [b], mean (95% CI), P<0.01 FI [e]: calcium: 2.3 (1.3, 4.1)	[a] National Health and Nutrition Examination Survey (NHANES) is a continuous, cross-sectional series of surveys of nationally representative samples of the resident, civilian, non-institutionalized US population; [b] Alternate health eating index for pregnancy (AHEI-P); [c] Adjusted for sociodemographic; food secure the reference category; [d] Food security (FS); [e] Food insecurity (FI); [f] Healthy Eating Index (HEI) measures adherence to the Dietary Guidelines for Americans based on adequacy and moderation components, with higher scores indicating better diet quality; [g] Behavioral Risk Factor Surveillance System (BRFSS)
Gonzalez-Nahm, 2022, (Central North Carolina)	Cross sectional study, n = 468, Female, Pregnant women, 22-33 y	10-item Household Food-security Survey Module (HFSSM) Adult Food-security Survey	Food Frequency Questionnaire (FFQ)	Estimated mean intake of food groups Vegetables, red and processed meat, dairy, fruit, whole grains, nuts, beans and legumes	Yes Multivariate [c] analysis of FS [d] and intake of food groups, β (95% CI), P<0.05 Vegetables: very low FS [d]: β= -0.43 (-0.81 to -0.05), red and processed meat: Marginal FS [d]: β=2.20 (0.20 to 4.19), low FS [d]: β=2.28 (0.17 to 4.39)	
Hill, 2020, A north-central county (North Carolina)	Cross sectional study, n = 198, Female, Pregnant women, 18-26+ y	18-item U.S. Household Food-security Survey Module (HFSSM)	Two 24-hour dietary recalls were averaged	Estimated mean difference in HEI [f]–2010 Total HEI [f] –2010	No	
Nunnery, 2017, Southeast region of the United States	Cross sectional study, n = 198, Female, Pregnant women, 26 y	18-item U.S. Household Food-security Survey Module (HFSSM)	The 2013 BRFSS [g] Fruit and Vegetable Food Frequency Questionnaire (FFQ) was used to assess the frequency of (daily, weekly or monthly) frequency of fruit and vegetable intake	Estimated mean intake of food groups	No	

Author, year, location (state)	Study design, participant characteristics	Food-security assessment method	Dietary assessment method	Dietary marker measured	Significant main findings: Is food-security associated to dietary intake?	Definition/Description
			**Pregnant Women**			
Shriver, 2023, (North Carolina)	Cross sectional study, N = 299, Female, Pregnant women, 24-35 y	6-item Short Form U.S. Household Food-security Survey Module (HFSSM)	The (DSQ) [a] was recorded to estimate daily intake of food groups/nutrients over the past month	Estimated mean intake of dietary markers<br>Total added sugar, added sugar from sugar-sweetened beverages, percentage energy from fat, and fruit/vegetables	Yes<br>Estimated mean intake of dietary markers, mean ± SD [b], P<0.05<br>FS [c]: total added sugars: 15.53 ± 4.27, added sugars from sugar-sweetened beverages: 6.18 ± 3.12, percentage energy from fat: 32.61 ± 4.13, fruit and vegetables: 2.55 ± 0.70<br>FI [d]: total added sugars: 17.28 ± 5.85, added sugars from sugar-sweetened beverages: 8.07 ± 5.03, percentage energy from fat: 34.34 ± 4.50, fruit and vegetables: 2.25 ± 0.60	[a] The Dietary Screener Questionnaire (DSQ) is a 26-item self-report screener developed by the National Cancer Institute (NCI); [b] Standard Deviation (SD); [c] Food security (FS); [d] Food-insecurity (FI); [e] The National Children's Study (NCS) Initial Vanguard Study (IVS), was designed as a large, longitudinal study to examine environmental influences on the health and development of U.S. children. Conducted between 2009 and 2010, the NCS-IVS evaluated the feasibility and yield of household-based sampling methods to recruit a diverse, nationally representative cohort of pregnant women; [f] NCI DHQ: The National Cancer Institute Diet History Questionnaire is a food frequency questionnaire that consists of 124 food items and includes both portion size and dietary supplement questions; [g] Healthy Eating Index (HEI) measures adherence to the Dietary Guidelines for Americans based on adequacy and moderation components, with higher scores indicating better diet quality
Sparks, 2024, NCS-IVS [e] from the United States	Cross sectional study, n = 679, Female, Pregnant women, 18-49 y	6-item Short Form U.S. Household Food-security Survey Module (HFSSM)	The NCI DHQ [f] Version 1 at trimester 1 and/or trimester 3 was used to measure dietary intake	Estimated mean HEI [g] -2015<br>Trimester 3 HEI[c]-2015 and energy	Yes<br>Estimated mean HEI [g] -2015, mean ± SD [b], P<0.05<br>FS [c]: Trimester 3 HEI [g]-2015: 62.6 ± 8.9<br>FI [d]: Trimester 3 HEI [g]-2015: 58.9 ± 8.7	

Author, year, location (state)	Study design, participant characteristics	Food-security assessment method	Dietary assessment method	Dietary marker measured	Significant main findings: Is food-security associated to dietary intake?	Definition/Description
**Multiple age groups (Household level)**						
Bhargava, 2007, NFSPS [a] from the United States	Cross sectional study, n=913 households, Female; Male; <18, >60 y	Households' food-security status	One week of food records	Estimated mean intake of dietary markers<br>Energy from added sugars (EAS), ratio: EAS/total energy use, protein use, ratio: protein/energy, calcium, ratio: calcium/energy, iron, ratio: iron/energy, ratio: beta-carotene/energy, ratio: vitamin A/energy, ratio: vitamin C/energy, ratio: fiber/energy, vitamin E, potassium, ratio: folic acid/energy, vitamin B6, ratio: vitamin B12/energy	Yes<br>Multivariate [b] analysis of the association between FS [c] and dietary markers, β ± SE [d], P<0.05<br>FI [e]: Protein: β = -0.041 ± 0.015, ratio: protein/energy: β = -0.046 ± 0.015, iron: β = -0.052 ± 0.022, ratio: iron/energy: β = -0.054 ± 0.022	[a] National Food Stamp Program Survey (NFSPS) from a United States national sample of 2142 food stamp participants; [b] Adjusted for sociodemographic, food secure the reference category; [c] Food secure (FS); [d] Standard Error (SE); [e] Food insecure (FI)
